# COMPILE: a GWAS computational pipeline for gene discovery in complex genomes

**DOI:** 10.1186/s12870-022-03668-9

**Published:** 2022-07-02

**Authors:** Matthew J. Hill, Bryan W. Penning, Maureen C. McCann, Nicholas C. Carpita

**Affiliations:** 1 Department of Botany and Plant Pathology, Purdue University, West Lafayette, Indiana, 47907 USA; 2 Present address: Whitehead Institute for Biomedical Research, 455 Main Street, Cambridge, MA 02142 USA; 3 Present address: Department of Biology, Massachusetts Institute of Technology, Cambridge, MA 02139 USA; 4 USDA-ARS Corn, Soybean and Wheat Quality Research Unit, Wooster, OH 44691 USA; 5 Department of Biological Sciences, Purdue University, West Lafayette, Indiana, 47907 USA; 6 Present address: Biosciences Center, National Renewable Energy Laboratory, 15013 Denver West Parkway, Golden, CO 80401 USA

**Keywords:** *Zea mays*, Maize, Genome, Computational biology, GWAS, QTL, γ-Tocopherol synthesis, Flowering time, *Ostrinia nubilalis*, European corn borer

## Abstract

**Background:**

Genome-Wide Association Studies (GWAS) are used to identify genes and alleles that contribute to quantitative traits in large and genetically diverse populations. However, traits with complex genetic architectures create an enormous computational load for discovery of candidate genes with acceptable statistical certainty. We developed a streamlined computational pipeline for GWAS (COMPILE) to accelerate identification and annotation of candidate maize genes associated with a quantitative trait, and then matches maize genes to their closest rice and Arabidopsis homologs by sequence similarity.

**Results:**

COMPILE executed GWAS using a Mixed Linear Model that incorporated, without compression, recent advancements in population structure control, then linked significant Quantitative Trait Loci (QTL) to candidate genes and RNA regulatory elements contained in any genome. COMPILE was validated using published data to identify QTL associated with the traits of α-tocopherol biosynthesis and flowering time, and identified published candidate genes as well as additional genes and non-coding RNAs. We then applied COMPILE to 274 genotypes of the maize Goodman Association Panel to identify candidate loci contributing to resistance of maize stems to penetration by larvae of the European Corn Borer (*Ostrinia nubilalis*). Candidate genes included those that encode a gene of unknown function, WRKY and MYB-like transcriptional factors, receptor-kinase signaling, riboflavin synthesis, nucleotide-sugar interconversion, and prolyl hydroxylation. Expression of the gene of unknown function has been associated with pathogen stress in maize and in rice homologs closest in sequence identity.

**Conclusions:**

The relative speed of data analysis using COMPILE allowed comparison of population size and compression. Limitations in population size and diversity are major constraints for a trait and are not overcome by increasing marker density. COMPILE is customizable and is readily adaptable for application to species with robust genomic and proteome databases.

**Supplementary Information:**

The online version contains supplementary material available at 10.1186/s12870-022-03668-9.

## Background

Association mapping is commonly used to mine genetic diversity in large populations for identification of the genes and alleles underlying complex traits [1, 2]. Genome-wide association studies (GWAS) offer the advantage of high-resolution mapping without the requirement to create mapping populations [3]. Using GWAS for candidate gene identification in maize (*Zea mays*) presents several challenges because of a complex and dynamic genome containing numerous transposable elements [4, 5]. However, the richness of maize genetic diversity, exemplified by single-nucleotide polymorphisms identified across thousands of maize genomes, has allowed high-resolution mapping of traits to candidate genes by GWAS [6–8]. Sequenced polymorphisms are captured in several genotyped populations, including the Goodman Association Panel of 282 inbred lines and landraces (Goodman AP) [9], the Nested Association Mapping (NAM) Recombinant Inbred Line populations of 5600 lines derived from 26 parental lines intermated with B73 [10], and the 2815-member U.S. Department of Agriculture-Agricultural Research Service (USDA-ARS) North Central Regional Plant Introduction Station (NCRPIS) populations [8].

Robust identification of genes involved in complex traits is generally improved by using larger populations [8, 11]. However, the use of large numbers of lines densely populated with sequence markers magnifies the computational demand for GWAS data analysis. Several data reduction techniques have been applied to GWAS, such as the use of compression in the Mixed Linear Model (MLM) method [2], collapsing individuals of similar genotypes into clusters treated as individual taxa [12]. A significant additional effort is required to annotate candidate genes within QTL intervals and to determine sequence variation in both coding and non-coding sequences.

Here, we demonstrate a semi-automated computational tool we call ‘COMPILE’, by which GWAS, executed using a more advanced statistical approach pioneered by Rincent et al. [13], revealed significant markers associated with maize candidate gene sequences. These markers were associated with their adjacent genes, and these genes aligned with rice (*Oryza sativa*) and Arabidopsis (*Arabidopsis thaliana*) public proteome databases to gain context from sequence-similar proteins in those species. This approach is generalizable to any species with lists of genetic features and proteomes in GFF and FASTA formats, respectively. We also developed several scripting tools useful in generating annotated figures displaying GWAS results in the common “Manhattan” plot format, both genome-wide and in smaller chromosome regions at high-resolution.

The COMPILE automated pipeline dramatically increases the speed of data analysis for researchers interested in GWAS. We validated COMPILE using published data from Chen and Lipka [14] on genes involved in γ-tocopherol synthesis in the developing embryo of maize caryopses, and from Romay et al. [8], on flowering time in maize. In addition to the candidate genes identified in each of these studies, additional genes associated with each trait were identified. Based on transcript profiling, a long non-coding RNA expressed for only a few hours in the nucellus of the developing kernel is associated with tocopherol synthesis. As proof-of-concept, COMPILE identified new candidate genes in the Goodman AP that might contribute to resistance of maize stems to penetration by larvae of the European corn borer (*Ostrinia nubilalis*). We show here that population size and diversity are major limitations in defining QTL and candidate genes contributing to this resistance. These limitations cannot be overcome by increasing marker density.

## Results

### COMPILE executes GWAS in an efficient one-step process

The COMPILE program executes GWAS by integration of phenotypic data with the K_chr Mixed Linear Model (MLM) without compression to generate a Manhattan plot, and then automatically searches for significant loci, matches those loci to adjacent maize genes, and finds the most similar sequences to those genes in rice and Arabidopsis (Dataset S1). This computational pipeline is adaptable to any species with structured genetically diverse populations that have been fully sequenced or well-populated with genetic markers. We built COMPILE on the GAPIT platform [15] using well-structured populations of maize as a test system. Use of COMPILE with established populations and published trait data confirmed the K_chr model [14], to be more robust for those populations. Use of the maize B73_RefGen_v4 genome assembly also allows non-coding RNA (ncRNA) features to be incorporated in the outputs.

Because COMPILE is built on the GAPIT platform, the parameters used within the GWAS are customizable. The system is a self-contained and self-constructing structure, including portable software installations and pre-installed R packages. COMPILE is made available on Github for ease of use and further development (https://github.com/mjacksonhill/COMPILE_Hill_et_al._2022_BMC_Plant_Biology). The speed of the analysis is improved in several ways: by pre-generation of the kinship matrices and annotation databases, by parallel execution of the MLM for all chromosomes, and by automated interpretation of significant results (Datasets S1-S3). The genotype files used in COMPILE are easily replaced with those produced or filtered by different parameters (different kinship algorithms, different marker filtering approaches, etc.), and we include scripts useful for processing these genotype files (Dataset S1). The parameters of the GAPIT execution are also modifiable within the COMPILE script (Dataset S2). Although we executed GWAS using a Mixed Linear Model without compression, the compression function can be customized into the analysis as described in Dataset S1. Association of identified significant markers is also possible either by selecting the nearest n genes or by specifying an LD window in base pairs, because LD can differ widely between different organisms. Different protein similarity databases could also be added in order to identify sequence-similar genes from other species.

Using a 7th generation Intel processor and 32GB of RAM, the approximate time to complete each analysis for a complete phenotype dataset (using the parallel chromosome processing feature of COMPILE when applicable) is approximately 5 min for the Goodman 2.7 version of COMPILE and 50 minutes for the NCRPIS 2.7 version of COMPILE.

To visualize chromosomal regions harboring QTL at high resolution takes 1 min per megabase using the FOCUS script. We observed a total compute time of ~ 2 days for full GWAS of the Goodman AP using the Goodman 3.2.1 marker set.

### COMPILE identifies novel genes for γ-tocopherol synthesis in the caryopsis

**Fig. 1:**
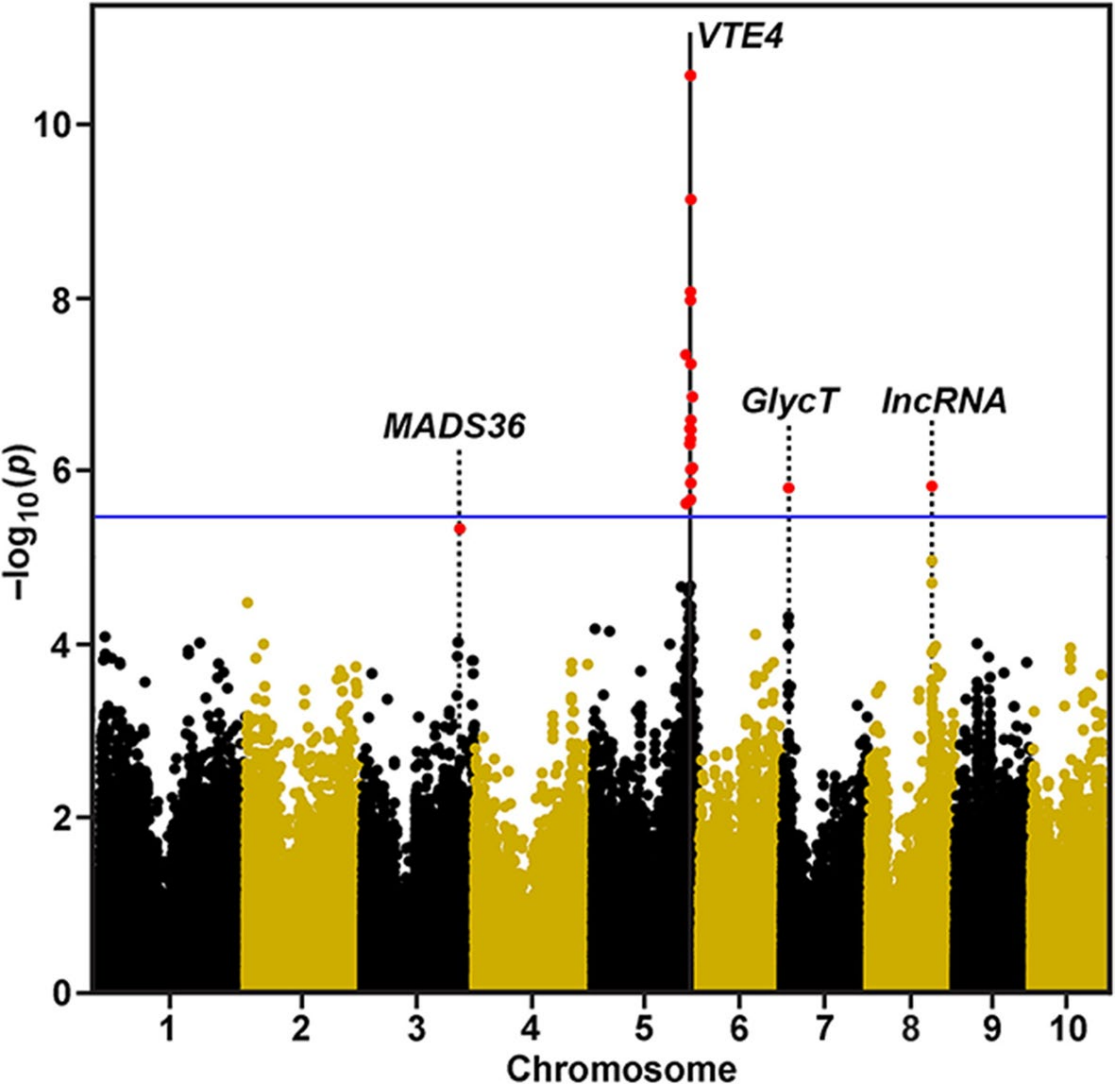
Manhattan Plot showing GWAS results for ratio of α-tocopherol to γ-tocopherol conversion. Data from Lipka et al. [16] were input into COMPILE for GWAS analysis and gene discovery. Negative log_10 _*p*-values are plotted against physical position (B73_RefGen_v4) on all 10 chromosomes. Values in red are significant at Benjamini-Hochberg false discovery rate of 5%. A visual marker for the Bonferroni threshold (averaged from the individual threshold y-values of each chromosome) at (α = 0.1) is indicated by the blue horizontal line. The vertical line marks the position of the maize tocopherol *O*-methyltransferase (*ZmVTE4*). Dotted lines indicate the positions of genes identified by COMPILE not identified in the original study: a MADS-box 36 transcription factor (Zm00001d043589), a putative glycosyl transferase gene (Zm00001d019057), and a the long non-coding RNA occurred very early in embryo development, and only between 84 and 96 h post-pollination in the nucellus [17]

We tested the analytical power and accuracy of COMPILE with published GWAS data obtained from the Goodman AP used to quantify the ratio of α- to γ-tocopherol in maize grain [16], as an example of a trait involving a small number of genes (Fig. 1, Table 1). This trait was among those revisited in Chen and Lipka [14], in their introduction of the K_chr model. We used a marker set similar to that of Chen and Lipka [14], but they used a random subset of 10% of their marker data to generate kinships, whereas we used the full set to generate kinships and did not include population structure covariates. Chen and Lipka [14] used the Benjamini-Hochberg protocol to control at 5% for significance. Consistent with the data from Lipka et al. [16], a SNP locus at about 205.8 Mbp on chromosome 5 lies within a window containing *ZmVTE4*, which COMPILE auto-annotated as a gene encoding a γ-tocopherol methyltransferase (Table 1). Three other genes were identified by Chen and Lipka [14] that were significant at a Benjamini-Hochberg FDR of 10%, an apparent cis-NAT pair of a transposase-like DUF659-containing protein and an EMBRYONIC FLOWER 1-like protein on Chromosome 8, and a gene of unknown function on Chromosome 7 (Table 1). However, COMPILE identified two additional strong QTL at an FDR of 5%, a MADS box-containing transcription factor gene (*MADS36*), homologous to those of Arabidopsis and rice related to floral identity and fruit development, and a long intergenic non-coding (linc) RNA that is the true *cis*-NAT pair with *EMF1* on Chromosome 8 (Fig. S1).

**Table 1:** Genes associated with QTL for α/γ-tocopherol ratio in maize kernels by COMPILE^a^

Chrom.	Marker	MLM	Distance	Maize Gene	Gene	BLAST	BLAST	BLAST	e-Value
	Position	***p***-Value	to Gene	Number	Name	Match	Description	Score	
3	204,491,509	4.76E–06*	− 2050	Zm00001d043589	MADS36	Os01g0726400	MADS box floral identity	299	1.93E–103
						At5g60910	AGAMOUS-like 8	107	6.29E–28
**5**	**205,827,506**	2.28E–12***	**−105**	**Zm00001d017746**	**VTE4**	**Os02g0701600**	**Tocopherol** ***O*-methyltransferase**	**543**	**0.0**
						**At1g64970**	**γ-tocopherol methyltransferase**	**420**	**2.31E–147**
**7**	**14,373,377**	1.61E–06**	**785**	**Zm00001d019057**	**Unknown**	**Os07g0189700**	**Similar to JHL07K02.7 protein**	**180**	**1.31E–56**
						**At3g23760**	**Glycosyl group transferase**	**129**	**2.87E–37**
**8**	**132,442,986**	1.54E–06**	**− 3544**	**Zm00001d010894**	**None**	**Os01g0229300**	**(EMF1)-like protein**	**624**	**0.0**
						**AT5G11530**	**Embryonic flower 1**	**43.1**	**0.013**
8	132,442,986	1.54E–06*	−231	Zm00001d008091	None	[lncRNA]	[Long non-coding RNA]	–	–
**8**	**132,442,986**	1.54E–06**	**867**	**Zm00001d010895**	**None**	**AT5G33406**	**hAT domain protein / transposase-like**	**115**	**6.17E–028**
						**Os09g0499600**	**DUF659 domain containing protein**	**114**	**3.32E–027**

^a^Phenotype data are from Lipka et al. [16]. All genes are identified by COMPILE as significant at a Benjamini-Hochberg FDR of 5%. Entries in **bold** indicate genes identified as significant by Lipka et al. [16] at a Benjamini-Hochberg FDR of 5% (***) or of 10% (**). Manual annotation of the long non-coding RNA is in brackets. *Not identified as significant in Lipka et al. [16]

As the four genes identified by COMPILE were not indicated in the original study to be involved in tocopherol synthesis [16], we examined a compendium of metadata on Maize Expression Atlas available through ePlant (bar.utoronto.ca), which was assembled from published data for caryopsis development [18]. *VTE4* expression in the developing caryopsis is largely confined to the embryo, scutellum and scutellar aleurone layer. All of the genes identified by COMPILE showed strong expression in tissues of the developing caryopsis (Fig. S2). Homologs closest in sequence in rice and Arabidopsis were annotated as floral identity genes, and the expression atlas showed high expression during kernel development. Expression of *EMF1-like* gene was highest in ear primordia and embryo, and during caryopsis development, but like *VTE4*, highest expression within the caryopsis was observed in the embryo, scutellum and scutellar aleurone layer, with moderate expression in the pericarp (Fig. S2). A gene encoding a DUF659-containing protein showed strong expression in the anthers, radicle and coleoptile, and embryo expression was confined to the aleurone adjacent to the scutellum. The putative glycosyl transferase gene displayed exceptionally high expression specifically in the scutellar aleurone layer.

### COMPILE identifies known and novel genes associated with flowering time

**Fig. 2:**
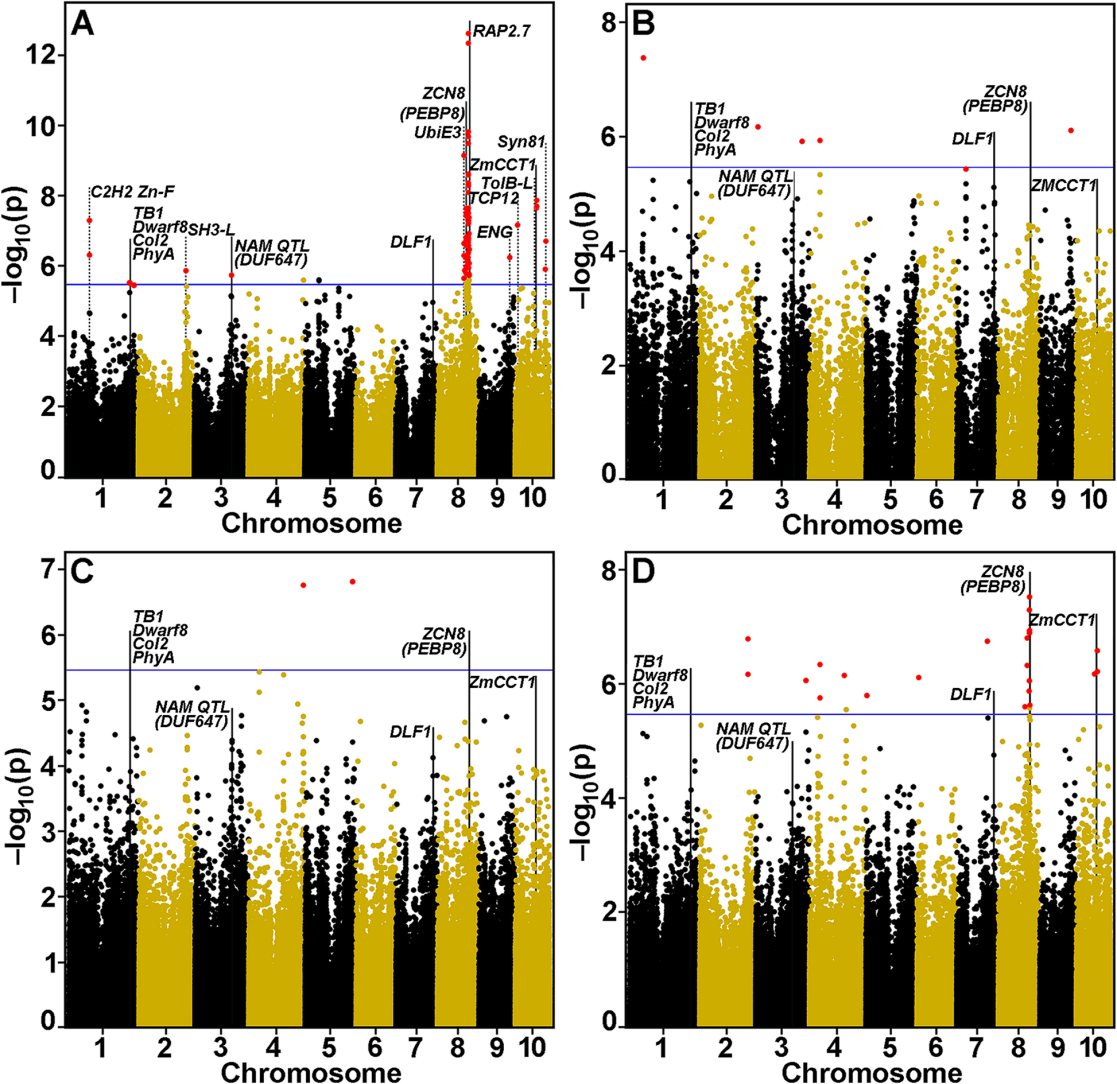
Manhattan Plot showing NCRPIS 2.7 and Goodman 2.7 GWAS results for flowering time. Data from Romay et al. [8] were used in GWAS analysis and gene discovery. Negative log_10 _*p*-values are plotted against physical position (B73_RefGen_v4) on all 10 chromosomes. Markers significant at a Benjamini-Hochberg false discovery rate of 10% are shown in red. A visual marker for the Bonferroni threshold (averaged from the individual threshold y-values of each chromosome) at (α = 0.1) is indicated by the blue horizontal line. Vertical solid lines indicate positions of significant QTL and gene annotations for flowering time, as shown by Romay et al. [8]. Dotted lines indicate positions of novel genes identified. Gene identities are described in Romay et al. [8]. **a** Results for GWAS conducted using the 2279-member NCRPIS population and NCRPIS 2.7 marker data. **b** Results for GWAS conducted using the 282-member Goodman AP collection within the NCRPIS population and Goodman 2.7 marker data. **c** Results for GWAS conducted using the 282-member Goodman AP collection within in the NCRPIS population and the nearest neighbor of similar phenotype (564 lines) and NCRPIS 2.7 marker data. **d** Results for GWAS conducted using the 282-member Goodman AP collection within in the NCRPIS population and three nearest neighbors of similar phenotype (1128 lines) and NCPRIS 2.7 marker data

We then applied COMPILE to published GWAS data obtained from the NCRPIS population used to identify loci that control the more complex trait of days-to-silking [8]. Using publicly available Best Linear Unbiased Polymorphisms (BLUPs) for the days-to-silking phenotype [8], COMPILE identified both known and novel QTL for this trait (Fig. 2a; Table 2). COMPILE recapitulated the findings of strong QTL attributed to known negative regulators of flowering time (Table 2). These include, on Chromosome 8, a *Phosphatidyl-ethanolamine binding protein8* (*PEBP8*) gene (formerly called *ZCN8* [19]), and the flowering repressor *ZmRap2.7*, and, on Chromosome 10, a *Circadian-Clock Time, ZmCCT1*, a gene that confers late-flowering (Fig. 2a). Also identified were, on Chromosome 3 coincident with a QTL identified in a maize NAM population, a gene encoding a DUF647-containing protein. The QTL cluster on chromosome 1, consisting of the sex- and internode-determinant *Teosinte Branched1*, flowering time-related *Dwarf8*, second photoperiodic response regulator *Col2*, and *Phytochrome A1*, and on chromosome 7 a QTLthe leucine-zipper transcription factor *Delayed Flowering1* (*DFL1*) gene, were also identified, but at *p*-values above the Bonferroni cutoff.

**Table 2:** Genes associated with QTL for growing-degree-day-adjusted days to flowering identified by COMPILE^a^

Chrom.	Marker	MLM	Distance	Maize Gene	Gene	BLAST	BLAST	BLAST	e-Value
	Position	***p***-Value	to Gene	Number	Name	Match	Description	Score	
1	94,342,987	5.19E–08	− 3107	Zm00001d029918	None	Os10g0324600	Zinc finger, C2H2 domain	48.9	4.31E–06
						At5g61190	C2H2-type zinc finger domain	41.6	2.00E–03
**1**	**[270554226]**	**–**	**–**	**Zm00001d033673**	**TB1**	**Os03g0706500**	**TCP Negative reg. lat. branch.**	**327**	**4.11E–110**
						**At1g67260**	**TCP family transcription factor**	**102**	**4.11E–110**
**1**	**[270919031]**	**–**	**–**	**Zm00001d033680**	**DWARF8**	**Os03g0707600**	**DELLA Gibberellin repressor**	**966**	**0.0**
						**At2g01570**	**GRAS transcription factor**	**648**	**0.0**
**1**	**[272191275]**	**–**	**–**	**Zm00001d033719**	**COL2**	**Os03g0711100**	**CONSTANS-like (COL)**	**454**	**5.95E–158**
						**At1g25440**	**B-box type Zn-finger/CCT domain**	**233**	**7.05E–72**
**1**	**[274082082]**	**–**	**–**	**Zm00001d033799**	**PHYA1**	**Os03g0719800**	**Phytochrome A, Photoreceptor**	**2064**	**0.0**
						**AT1G09570**	**Phytochrome A**	**1518**	**0.0**
2	208,886,674	1.41E–06	2175	Zm00001d006461	None	Os07g0508300	SH3 domain-containing protein like	642	0.0
						AT4G18060	SH3 domain-containing protein	462	3.30E–164
**3**	**162,993,079**	**1.85E–06**	**− 1834**	**Zm00001d042355**	**None**	**Os04g0290800**	**Predicted protein**	**813**	**0.0**
						**At3g45890**	**RUS1 UVB sensitive-like (DUF647)**	**596**	**0.0**
7*	**[181090142]**	**–**	**–**	**Zm00001d022613**	**DLF1**	**Os09g0540800**	**Leucine zipper (β-ZIP)**	**125**	**2.73E–36**
						**At2g17770**	**Leucine zipper motif 27**	**79.7**	**1.90E–18**
8	116,806,871	7.35E–10	1222	Zm00001d010470	UbiE3	AT5G13530	E3 Ubiquitin-protein ligase	2198	0.0
						Os05g0392050	UbiE3 Ubiquitin-ligase	349	4.91E–108
**8**	**126,885,610**	**2.34E–08**	**− 4150**	**Zm00001d010752**	**PEBP8**	**Os05g0518000**	**Hd3a, Promotor flowering**	**279**	**2.17E–97**
						**At1g65480**	**PEBP family protein**	**219**	**5.26E–73**
**8**	**136,010,257**	**1.79E–07**	**393**	**Zm00001d010987**	**RAP2.7**	**Os05g0121600**	**AP2/EREBP**	**396**	**2.34E–135**
						**At4g36920**	**Integrase-type DNA-binding**	**283**	**3.55E–92**
9	135,980,380	5.87E–07	−242	Zm00001d047573	F-box	Os03g0321300	Cyclin-like F-box domain protein	533	0.0
						AT5G46170	F-box family protein	308	1.47E–101
10	10,315,088	7.02E–08	− 914	Zm00001d023565	TCP	AT1G58100	TCP family transcription factor	204	9.25E–62
						Os12g0173300	Transcription factor, TCP protein	366	2.12E–124
10	92,331,033	1.41E–08	361	Zm00001d024885	WD40-like	Os03g0403400	TolB-like domain β-propeller	1065	0.0
						At1g21680	DPP6 N-terminal domain-like	351	1.23E–110
**10**	**94,277,277**	**2.04E–08**	**154,896**	**Zm00001d024909**	**CCT1**	**Os07g0261200**	**CCT (CONSTANS)**	**138**	**4.40E–40**
						**At5g24930**	**Zn-finger CONSTANS-like**	**78.2**	**1.54E–16**
10	133,736,251	2.03E–07	244	Zm00001d025915	Syntaxin81	At1g51740	Syntaxin81	185	6.42E–59
						Os04g0530400	t-Snare domain containing protein	255	1.34E–86

^a^Phenotype data are from Romay et al. [8]. All genes are identified as significant by COMPILE at a Benjamini-Hochberg FDR of 5%. Entries in **bold** are those identified as significant by Romay et al. [8]. Genes without *p*-value or gene distance annotations represent genes not identified by COMPILE. For these genes, “Marker Position” annotations in brackets represent gene midpoint coordinates. Entries in normal text were identified as significant by COMPILE but not by Romay et al. [8]

Six additional strong QTL were identified using COMPILE that were not observed by Romay et al. [8]. These included genes encoding on Chromosome 1, a C2H2 Zinc-finger domain protein, on Chromosome 2, a gene encoding a tyrosine kinase SH3 (Src-domain) protein involved in uncoating clathrin [20], on Chromosome 9, a gene encoding a cyclin-like F-box domain protein, and, on Chromosome 10, genes encoding transcription factor Teosinte branched1/Cincinnata/Proliferating cell factor (TCP), a TolB-like protein, and a Syntaxin81 (SYP81) t-SNARE-type protein (Fig. 2a; Table 2).

We then compared published relative expression data across plant organs for genes reported by Romay et al. [8], as contributing to the flowering time trait (Fig. S3) from a compendium of metadata for whole plant expression [21, 22], in the Maize Expression Atlas (bar.utoronto.ca). The *Dwarf8*, *PhyA1*, and *DLF1* were more broadly expressed, with significant internode and ear primordia expression, whereas *RAP2.7* was expressed in germinating grains and roots. *Dwarf8* and *PhyA1* were expressed in the silk tissues. *TB1* was only expressed in ear primordia and female spikelets, whereas the *CONSTANS-like Col2* and *PEBP8* genes were expressed almost exclusively in leaves (Fig. S3). In addition to the genes contributing to flowering time identified by GWAS [8], COMPILE identified eight additional genes that might contribute to the trait. All but one gene was expressed in ear primordia and female spikelets, indicating an association with flowering organs. A gene encoding a C2H2-Zn finger-containing domain was expressed in the female spikelet, in ear primordia and embryo, while genes encoding an SH3-domain protein, a Syntaxin81 and a UbiE3 had highest relative expression in ear primordia (Fig. S4). The *TolB-L* gene had moderate expression in young leaves but was the only gene without significant expression in ear primordia or embryo.

### Larval penetration by the European corn borer (*Ostrinia nubilalis*) is a quantitative trait within the Goodman AP

**Fig. 3:**
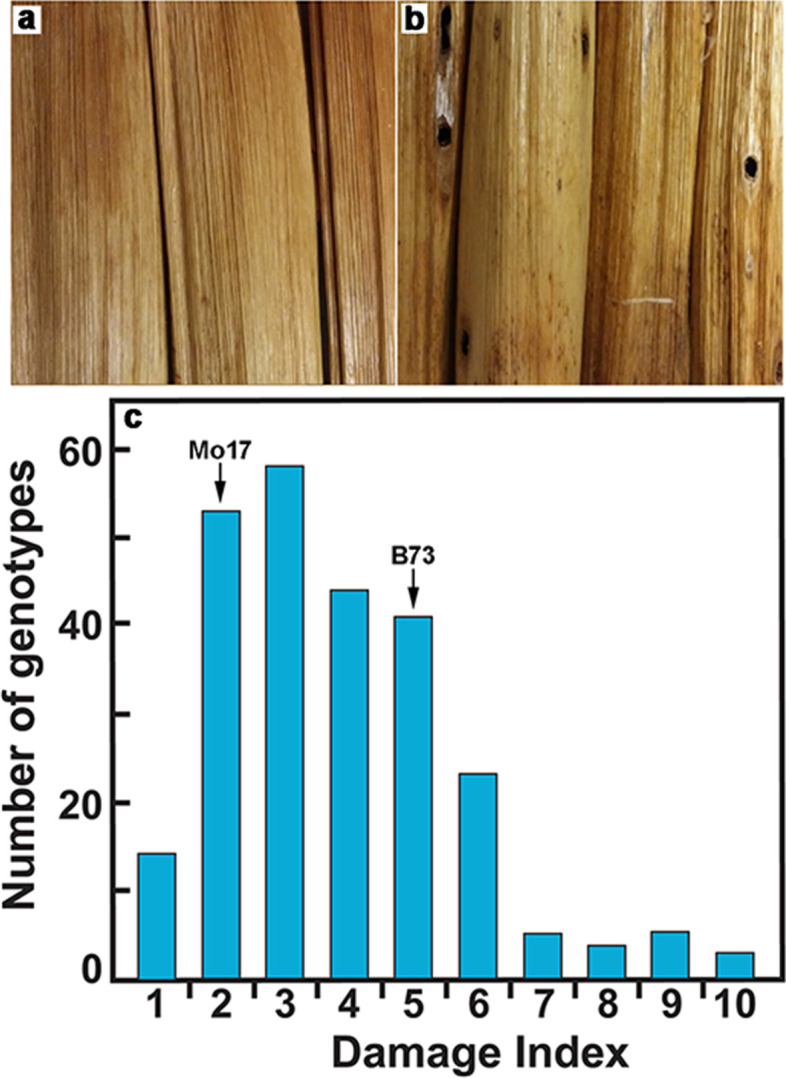
Micrographs of internodes of maize and histogram of insect damage. **a** Maize internodes without European corn borer damage. **b** Maize internodes with holes remaining after penetration of the corn borer larvae. **c**. Frequency distributions for insect damage in the Goodman AP. The two populations were normalized and assigned a damage index from 1, with zero damage, to 10, in increments of 0.032 holes/cm of internode length, with an average of 0.11 holes/cm of internode length, corresponding to an average index of 3.28. Mo17 with an index of 0.05 falls in bin 2, and B73 with an index of 0.15 falls into bin 5

During the course of studies of maize stem development in the Goodman AP population, we observed during collection of the stover that wide variation existed in the extent of damage to the pith by the European corn borer (*Ostrinia nubilalis*). Stem damage during penetration of the larvae of European corn borer (*Ostrinia nubilalis*) at senescence varied widely among 274 field-grown genotypes of the Goodman AP collection (Fig. 3, a and b). The amount of internal damage in the pith correlated with the number of entry holes made by larvae along the internodes. We counted the number of entry holes created by larvae per unit of internode length and plotted the frequency of borer damage (Fig. 3c). Two-dimensional heat maps of field planting position against insect damage showed little to no correlation between damage and position within the field (Fig. S5), and quantile-quantile plots of the distribution of damage were generally linear except at extremes, which showed positive skewing (Fig. S6). Senescent internodes 4 and 5 were milled, and cell walls were isolated for analyses of cellulose and lignin composition. Pearson’s correlation coefficients measured for each trait showed insignificant correlation between insect damage and lignin composition (G-lignin *R*^2^ = 0.006; S-lignin *R*^2^ = 0.001), cellulose abundance (*R*^2^ = 0.003), and stover density (*R*^2^ = 0.000) (Fig. S7).

### Larval penetrance QTL contain candidate resistance genes

**Fig. 4:**
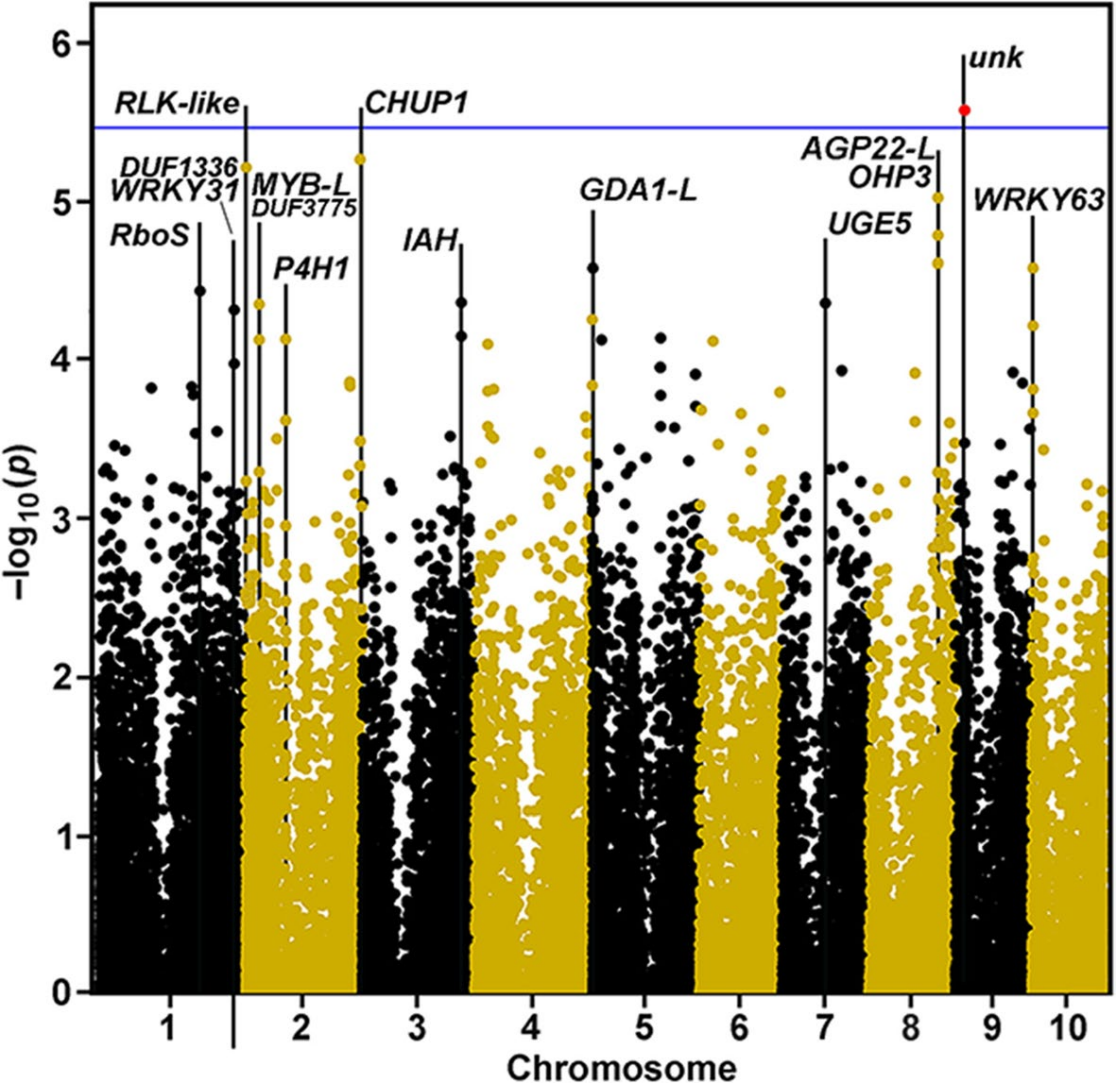
Manhattan Plot showing GWAS results for insect damage index in the Goodman AP using Goodman 2.7 data. Negative log_10 _*p*-values are plotted against physical position (B73 RefGen_v4). A visual marker for the Bonferroni threshold (averaged from the individual threshold y-values of each chromosome at (α = 0.1) is indicated by the blue horizontal line. One location, red circle, was significant. Gene identities are described in Table 3

Several candidate genes for entry hole data were identified at the *p*-value (−log_10_* p*) ≤ 1 × 10^− 4^ level in the Goodman AP (Fig. 4). The QTL with the lowest *p*-value, the only one significant with a Bonferroni threshold at α = 0.1, corresponded to a gene of unknown function located on Chromosome 9. The unknown protein of 123 amino acids and molecular weight of 12.6 kDa has a predicted signal peptide, and Kyte-Doolittle analysis across an 11-amino-acid window predicted that in addition to the signal peptide, one small hydrophobic domain is located upstream and one broad domain is located downstream of a central Arg- and Pro-rich cytoplasmic domain (Fig. S8a). Owing to the abundance of Arg residues throughout the protein, the pI is 10.3. Two rice homologs with over 90% sequence identity displayed similar hydropathy patterns and a search at NCBI using BLAST (https://blast.ncbi.nlm.nih.gov/Blast.cgi?PAGE=Proteins) revealed homologs with high similarity mostly in grass species (Fig. S8, a-f). The rice sequences were nearly identical except for an extended *N*-terminus in the japonicum cultivar (Fig. S8, b and d). A shorter cytoplasmic domain occupies the *N*-terminal domain after the signal peptide, and the *C*-terminus contains a strongly hydrophilic SSRDDS common to a leucine-rich repeat Receptor-Like Kinase (RLK), but the proteins share no other sequence that would indicate homologous function. Neither maize nor rice sequences match any known protein. BLAST alignments show short sequences in the middle cytoplasmic regions align to a *Ribonuclease Regulating Protein* gene (*RraA*). A putative sugar transporter and a gene encoding a choline *O*-acetyltransferase (CHAT) domain-containing protein also contain short alignments in a strongly hydrophilic domain sandwiched by large hydrophobic domains, but overall these proteins are unrelated.

**Table 3:** Genes associated with QTL for European corn borer stem penetration

Chrom.	Marker	MLM	Distance	Maize Gene	Gene	BLAST	BLAST	BLAST	e-Value
	Position	***p***-Value	to Gene	Number	Name	Match	Description	Score	
1	211,798,518	3.67E-05	1079	Zm00001d032079	RboS-L	Os09g0438000	Riboflavin synthase-like	1418	0.0
						At1g19230	Riboflavin synthase-like	1093	0.0
1	283,651,183	4.84E-05	− 1304	Zm00001d034084	WRKY31^a^	At3g01080	WRKY DNA-binding (WRKY58)	218	1.52E-66
						Os12g0507300	WRKY DNA binding (WRKY96)	231	4.72E-72
1	283,651,183	4.84E-05	1803	Zm00001d034085	DUF1336^a^	At3g29180	DUF1336	427	4.06E-147
						Os03g55180	DUF1336	577	0.0
2	1,413,891	6.11E-06	852	Zm00001d001813	RLK-L^a^	Os04g42620	Ser/Thr RLK-like	634	0.0
						Os03g0759000	LysM RLK-like	407	3.26E-141
2	28,958,597	4.46E-05	− 2883	Zm00001d002991	MYB-L^a^	Os06g0190900	MYB-SANT-like protein	164	1.65E-502
						[At3g11290]	Unknown	38	0.007
2	84,078,171	7.44E-05	− 4593	Zm00001d004120	P4H	At2g43080	P4H isoform 1	313	6.39E-109
						Os04g0346000	P4H1	118	1.18E-34
2	239,762,997	5.45E-06	− 644	Zm00001d007788	CHUP1	Os07g0188266	FKBP-type peptidyl-prolyl isomerase	285	6.45E-93
						At3g25690	CHUP1	42	6e-04
3	207,872,378	4.36E-05	2565	Zm00001d043701	IAH	Os01g0706900	Similar to Auxin amidohydrolase.	639	0.0
						At1g51760	IAA-Ala peptidase	488	1.71E-171
5	981,132	2.63E-05	418	Zm00001d012838	GDA1-L	Os05g0498700	GDA1-like	461	6.23E-164
						At3g27090	DCD domain protein	328	1.25E-111
7	91,467,624	4.37E-05	− 5168	Zm00001d020093	UGE5	At4g10960	UDP-D-Glc epimerase5 (UGE5)	228	5.79E-75
						Os09g0323000	UDP-D-Glc/UDP-D-Gal 4-epimerase	261	3.36E-88
8	144,702,617	9.44E-06	− 4196	Zm00001d011256	OHP3	At1g34000	One-helix LHC protein2 (OHP2)	160	3.13E-50
						Os01g0589800	High-light inducible protein	145	5.29E-46
8	144,702,617	9.44E-06	16,216	Zm00001d011257	AGP22-L^a^	At5g53250	Arabinogalactan protein22	45.1	4.50E-8
						Os01g0592500	DUF1070 family protein	39.3	6.46E-6
9	19,551,832	2.67E-06	109	Zm00001d045360	None	Os06g0147300	Unknown	107	3.55E-31
						At5g13000	Unknown	27	5.5
10	3,599,714	6.09E-05	881	Zm00001d023332	WRKY63^a^	Os09t0334500	WRKY74	86	1E-17
						At2g40750	WRKY54	76	3E-15

^a^Gene identity defined manually by alignment with rice and Arabidopsis homologs closest in sequence

Two QTL with near-significant *p*-values ≤1 × 10^− 5^ were located on Chromosome 2, including a Ser/Thr receptor kinase-like protein and a spectrin repeat-containing *CHUP1* homolog involved in chloroplast movement and anchoring to the plasma membrane. On Chromosome 8 was a one-helix protein gene (*OHP3*) involved in photoprotection in the light-harvesting complex during light stress. Although significant to *p*-values of only ≤1 × 10^− 4^, on Chromosomes 1 and 2 were located *WRKY31* and *MYB-L* transcription factor genes, respectively. Other genes of interest near significance included, on Chromosome 1, a *Riboflavin synthase-like* gene (*RboS-L*), on Chromosome 2, a *Prolyl 4-hydroxylase* (*P4H1*) gene involved in hydroxyproline synthesis, on Chromosome 3, an *IAA-Ala Amino Hydrolase* (*IAH*) gene involved in yielding IAA from amino acid conjugates, on Chromosome 5, a *Guanosine Diphosphatase1* gene (*GDA1*) involved in transport of GDP-Mannose into the lumen of the Golgi apparatus, on Chromosome 7, a *UDP-Glu:UDP-Gal 4-epimerase* gene (*UGE5*), and, on Chromosome 10, a *WRKY63-like* transcription factor gene (Fig. 4, Table 3).

The incorporation of the high-density Goodman 3.2.1 marker set was particularly useful in discriminating between genes close to markers. As some of these QTL were not easily assignable to a single gene due to local genome architecture, we used results from both the 2.7 and 3.2.1 Goodman versions of the analysis. Examination of GWAS results at higher resolution enabled us to confidently map several QTL to genes, including, on Chromosome 9, the unknown gene (Fig. S9a), on Chromosome 10, a *WRKY63* (Fig. S9b), and on Chromosome 8 a locus 3′ of the *OHP3* gene and upstream of an *Arabinogalactan-protein22-like* gene (*AGP22-L*) (Fig. S9c). One near- significant location on Chromosome 1 is positioned 3′ of both a *WRKY31* and a gene encoding a DUF1336-containing protein forming a *cis*-NAT pair (Fig. S9d).

### Expression of candidate genes during maize stem development

**Fig. 5:**
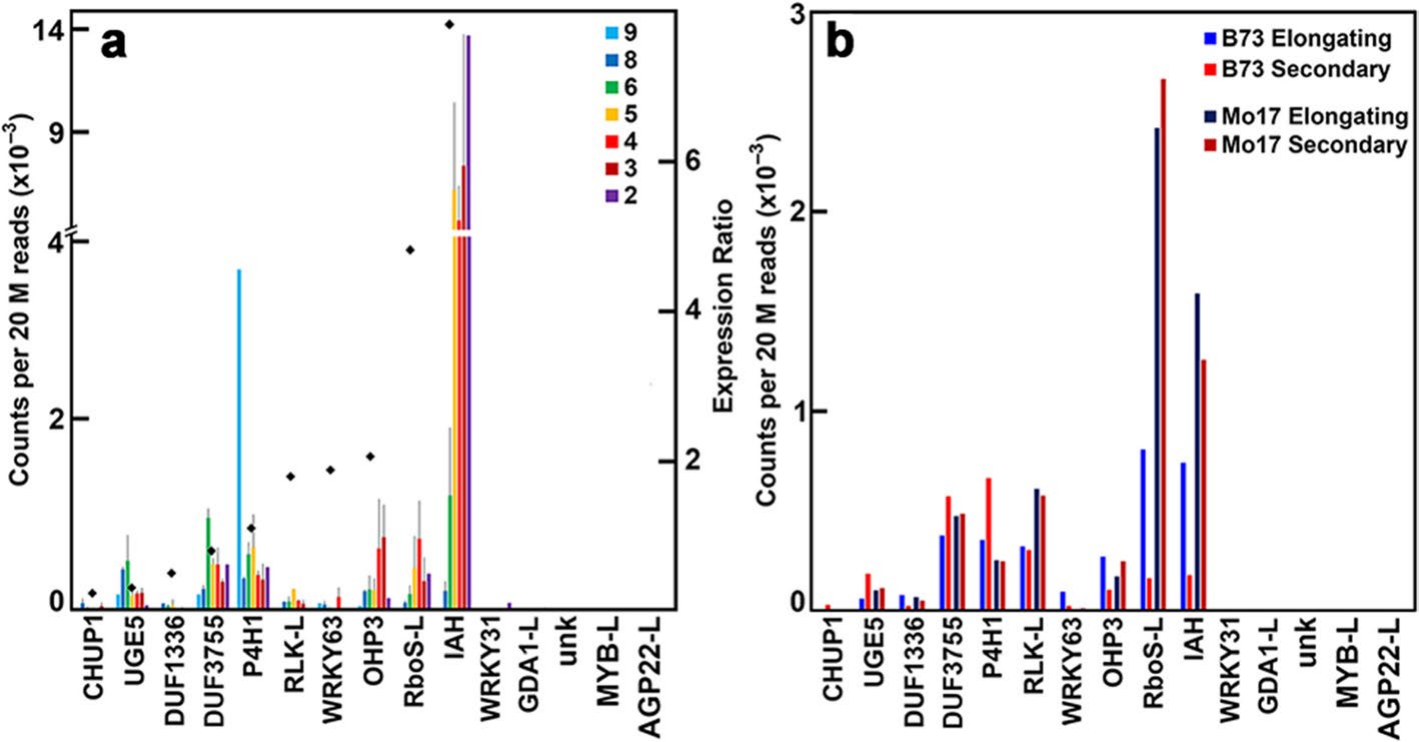
Expression of genes associated with insect damage in developing stems of field-grown and greenhouse-grown maize. **a** Expression in maize B73 of genes associated with larval penetrations during stem development. Transcript levels in rind tissues from Internodes 9 through 2 from field-grown plants were normalized and compared as counts per 20 M reads. Values are the mean ± variance or S.D. of two or three independent rind collections, respectively. Genes with expression greater than 500 reads per 20 M were ordered by their ratio of expression (black diamonds) in secondary cell-wall-forming tissues (Internodes 5 and 4) to elongating tissue (Internodes 8 and 6). **b** Differential expression in maize B73 and Mo17 of genes associated with larval penetrations during stem development. Transcript levels in rind tissues of greenhouse-grown plants taken at elongation stages (Internodes 8 and 6) and secondary wall synthesis stages (Internodes 5 and 4) of each inbred were pooled and normalized and compared as counts per 20 M reads

In a previous study, we provided a comprehensive inventory of the cell-wall genes differentially expressed in rind tissues of individual internodes during the development of the maize B73 stem [23]. We defined several complex expression patterns associated primarily with elongation growth or secondary wall synthesis. Of the candidate genes identified by GWAS as contributing to larval penetration, *IAH* was more highly expressed in the lower internodes (Fig. 5a). The *OHP3*and *RboS-L* genes, although much lower in expression, were expressed in a secondary wall-related pattern. By contrast, *P4H1* and *UGE5* were expressed with greater relative expression during primary wall formation. The *RLK-L* and *WRKY63* genes were weakly expressed in all internodes (Fig. 5a). The *WRKY31*, *GDA1-L*, *MYB-L*, *AGP22-L*, and the unknown gene were not expressed above 100 reads in any internode during the time-course of stem development.

We also compared expression between Mo17, which had a low borer damage index of 0.05 (bin 2), with that of B73, which gave an index of 0.15 (bin 5) (Fig. 3c). Greenhouse-grown plants free of insect pests were sampled at 49 days post-planting, where internodes 6 and 8 represented the elongation stage, and internodes 4 and 5 represented peak secondary wall formation [23]. Slight differences in expression between Mo17 and B73 were observed at either developmental stage with two exceptions. Transcripts of the *RboS-L* and *IAH* genes in B73 were less abundant at the secondary wall stage in greenhouse-grown plants than those sampled in the field, and the IAH, in particular, was expressed at over 10-fold higher levels in the field-grown plants (Fig. 5b). Both genes were much more highly expressed at both developmental stages in the greenhouse-grown Mo17 plants. Genes that were not expressed in B73 in field- or greenhouse-grown plants, were also not expressed in Mo17 plants (Fig. 5b).

As described for the genes identified in the Romay et al. [8] dataset, we probed the Maize Gene Expression Atlas for genes associated with larval stem damage. Five of the genes indicated by GWAS, including the unknown with the strongest *p*-value, exhibited insignificant expression in any internode of either B73 or Mo17 (Fig. 5). However, probing for whole plant organ expression, we found that all of them are expressed in other organs. Eight of the genes associated with larval damage exhibited significant expression in the internode, and five of these also showed strong expression in ear primordia and embryonal tissues. (Fig. S10). Seven other genes without significant internode expression displayed more specialized expression (Fig. S11). *WRKY31* was also expressed only in roots, and expression is enhanced by drought stress but not pathogen stress. The *AGP22-L* gene was strongly expressed in mature leaves. Although we detected no expression in any internode, *GDA1-L* was broadly expressed across all organs. The Maize Gene Expression Atlas showed the unknown gene to exhibit solely highest relative expression in roots.

Although the Maize Gene Expression Atlas does not evaluate differential expression induced by insect damage, we probed the atlas for responses to abiotic and biotic stress as contributed by Hoopes et al. [22], Opitz et al. [24], Makarevitch et al. [25]. The unknown gene was unresponsive to abiotic drought, salt or temperature stress, but strongly upregulated in leaves upon infection with *Colletotrichum* or *Cercospora* fungi (Fig. S12a). Cell-wall related genes *P4H* and *UGE5* also exhibited enhanced leaf expression upon fungal inoculation (Fig. S12, b and d). Enhanced expression of AGP22-L, IAH, and RLK-L was also recorded in young and old leaves, but expression of AGP22-L and IAH were also enhanced by salt stress (Fig. S12, c, e, and f).

### Limitations of population size and marker density in establishing significant QTL

Candidate loci containing genes that might contribute to resistance to corn borer penetration achieved *p*-values of only ≤10^− 5^. Although the Goodman AP was sufficient to identify traits governed by a small number of genes (Fig. 4), we tested the impact of population size and structure on candidate gene identification using the complex trait of days-to-silking from Romay et al. [8]. When GWAS was applied to the Goodman AP alone represented in the 2815-member NCRPIS collection, five major loci were identified with *p*-values of ≥10^− 6^. To test the minimum population size needed for the major QTL to appear significant within the results, we created populations that paired the 282 Goodman AP lines with their nearest one and three neighboring lines from the NCRPIS population in trait value (564 lines and 1128 lines, respectively), and performed GWAS using the same NCRPIS 2.7 marker set [8]. The 564-line population was insufficient to improve the LOD values to below *p* = 10^− 5^, but the 1128-line population captured the *PEBP8*, *RAP2.7*, and *CCT1* loci above the Bonferroni cut-off (Fig. 2). However, both subpopulations indicated other loci of significance not seen in GWAS of the full population.

We next determined if the LOD values could be improved for the *PEBP8* and *ZmRAP2.7* loci in the Goodman AP if marker density was increased. For this, we analyzed the region containing both QTL using the Goodman marker sets 2.7 and 3.2.1. The results using marker set Goodman 3.2.1 defined both *PEBP8* and *RAP2.7* within the 110 to 150 Mbp window of Chromosome 8, but with no improvement of LOD value (Fig. S13).

## Discussion

### Validation of COMPILE

By integrating the K_chr model into the advanced GAPIT platform, we aimed to streamline GWAS with methods and scripts that rapidly generate the marker and kinship files (Dataset S1). We made these tools adaptable for any species with diverse populations. The introduction of the K_chr approach also presents the benefit of parallel processing, which reduces GAPIT run time by nearly an order of magnitude given sufficient memory and processing power. In addition, it alleviates the need for compression, which is another approach designed to avoid the confounding effect of testing a marker using a kinship structure including that marker. The construction of COMPILE also presents a major resource for rapid gene annotation, reducing a manual process to a simple automated process which executes within a few seconds for all candidate markers below a user-defined *p*-value. (Dataset S1). Our tools to plot GWAS results against genome architecture facilitate data visualization (Dataset S3). We found that all versions of COMPILE execute rapidly, even for large populations and marker sets.

We tested several parameters to probe the limitations of population size and marker density. Our reproduction of published GWAS highlights the improvement by larger populations that capture the broadest maize genetic diversity. Inclusion of additional markers did not increase statistical power in small populations, but they did increase resolution for identification of genes when using the FOCUS script. Our approach also highlights how small differences in protocols, such as marker set preparation and filtering approaches, kinship generation, and the inclusion of population structure covariates, can lead to different results with the same data. Although it is difficult to account for the best data preparation practices for every quantitative trait, the flexible framework of COMPILE and the methods made available here should allow fully customizable dataset preparation and GWAS execution.

We also used published expression data and our own RNAseq data during maize stem development at the time of corn borer infestation as additional validation that candidate genes identified were expressed at this time of development. In discriminating lignin abundance and saccharification yield in stover in two maize inbred lines, we performed whole genome differential expression to validate candidate genes within a QTL [26]. By coupling QTL mapping with transcriptome analysis, maize hypersensitive defense response genes that cluster in associated pathways were identified [27]. Differential expression of phenylpropanoid-related genes showed which were associated with lignin-abundance QTL [28], and combining QTL mapping with transcriptome sequencing revealed candidate genes for flowering time in Brassica species [29].

### Novel genes associated with tocopherol synthesis and flowering time

COMPILE iterated identification of the *VTE4* gene discovered by Chen and Lipka [14], as the major contributor to γ-tocopherol synthesis in the developing embryo of maize caryopses. According to the Maize Gene Expression Atlas (bar.utoronto.edu), additional genes associated with this trait, and identified by COMPILE were expressed in developing caryopses but with different patterns. Based on transcript profiling, the long non-coding RNA is expressed for only a few hours in the nucellus [17].

We also examined the expression profiles of the genes indicated by Romay et al. [8] to gain insight into their potential complexity of interaction. The two strongest candidates, *RAP2.7* and *PEBP8*, had distinct expression patterns. Whereas *RAP2.7* expression was primarily in root and germinating grains, *PEBP8* was almost exclusively expressed in mature leaves (Fig. S3). Of the four genes clustered on Chromosome 1, *DWARF8* and *PhyA1* were broadly expressed, but *Col2* expression was essentially confined to mature leaf and the female spikelet and *TB1* had almost exclusive expression in the ear primordia and female spikelet (Fig. S3). By contrast, seven of the eight additional genes identified by COMPILE exhibited broad expression, including expression in ear primordia and female spikelet, indicating that they at least expressed in floral organs (Fig. S4). The exception was the *TolB-like* factor that had more broad expression, but was not expressed in ear primordia and young embryos.

### Novel resistance genes for larval penetration

The European corn borer is a major maize pest in the US and Europe. Borers have a bivoltine growth habit, with the first generation feeding primarily on leaves and the second feeding on leaf sheath and stalk tissues [30]. Damage caused by stalk tunneling can result in broken stalks and lodging, but yield loss is attributed primarily to poor ear development. The challenge has been to identify the genetic basis for the second-generation larval damage [31]. Resistance to tunneling damage has been reported in inbred lines [32], and QTL have been established in recombinant inbred populations derived from resistant and susceptible parents [33, 34]. A laccase and a lignin-related cinnamoyl-3-hydroxylase were associated with tunneling resistance in a Multi-parent Advanced Generation InterCrosses (MAGIC) population [35].

Larvae must first penetrate the outer rind tissues to gain access to the pith, and differences in stalk strength and penetration resistance have also been evaluated as quantitative traits independent from pith tunneling [31, 36, 37]. The diversity of maize cell-wall hemicellulose, cellulose, and lignin composition [38], and hydroxycinnamate ester content [39], have also been targeted for QTL analysis for correlations with insect damage. When penetration resistance was assayed across Intermated B73 x Mo17 (IBM), NAM and NCRPIS populations, genes encoding a *4-Coumarate-CoA Ligase* and a *Caffeoyl-CoA O-methyltransferase* were linked to loci for penetrometer resistance, whereas only a single *Cellulose Synthase9* gene was identified [36]. We found little or no correlation between number of larval entrance holes and the bulk property of cellulose content or lignin content or composition (Fig. S7) and, thus, resistance to larval penetration must rely on more subtle alterations in wall composition and architecture. Nevertheless, rind penetrometer resistance, as a measure of stalk strength, indicate genes encoding enzymes of wall metabolism, such as a laccase, a UDP-GlcA decarboxylase, which generates UDP-Xyl, a pectin methylesterase, and a pectate lyase [40]. We found association with two cell wall-related genes, including prolyl 4-hydrolase1 (P4H1), which converts proline to hydroxyproline, and a UDP-Glc:UDP-Gal epimerase (UGE5) that interconverts these two nucleotide sugars (Fig. 4). The P4Hs are required for substrate synthesis for hydroxyproline-rich glycoproteins (HGRPs), which are known to play important roles in plant defense by insolubilizing and oxidatively cross-linking under stress, strengthening the cell wall [41]. Hydroxyprolines, along with Ara and Gal components of the glycans, are components of arabinogalactan-proteins (AGPs), which also play roles in defense [42]. UGE5 is essential in this synthesis [43].

Tunneling and stem damage resistance have been associated with genes of signaling pathways rather than those of wall synthesis. Among QTL for tunneling resistance were genes encoding a hexokinase1, a phospholipase A2, a histidine kinase, and a Ca^2+^/calmodulin-dependent protein kinase, whereas the stem damage was associated with an actin depolymerizing factor, an LRR receptor-like kinase, and two uncharacterized proteins [37]. However, lack of overlap between QTL defined in this study, and those by several others [33, 35, 36, 44], underscores the strong gene × environment variation in genes that contribute to resistance and the difficulties in comparisons between different populations, methods of data analysis, and trait proxies used as assays.

An *RLK*, *CHUP1*, and an *OHP3* were significant at a *p*-value of ≤10^− 5^ (Fig. 4). The Arabidopsis and rice proteins closest in sequence to the *RLK* is *De-Etiolated1* (*DET1*), whose products mediate cross-talk in abscisic-acid signaling pathways during growth and development [45]. *Chloroplast Unusual Positioning 1* (*CHUP1*) encodes a chloroplast outer envelope protein that mediates anchorage to the plasma membrane and mediates chloroplast intracellular redistribution in response to light intensity [46], and one-helix proteins (OHPs) are part of the light-harvesting complex, where they participate in sensing of light intensity and energy dissipation, as well as triggering of photomorphogenesis [47]. If expression of these genes in the rind tissues in response to larval penetration, a broader role of stem chloroplast function beyond photosynthesis is inferred. Other significant hits with *p*-values ≤10^− 4^ include genes that encode WRKY and MYB regulators, enzymes for a member of the riboflavin synthase pathway, a hydrolase of IAA-amino acid conjugates, and GDA1 involved in GDP-Mannose transport. A wheat homolog closest in sequence to the Arabidopsis IAH gene (IAR3), TalAR3, was shown to have much stronger substrate specificity for the long side chain auxins indole butyric acid (IBA) and indole propionic acid (IPA) [48]. Nehela et al. [49] showed that these auxins were increased during herbivory by *Diaphorina citri*, especially when the insect vector transmitted a *‘Candidatus Liberibacter asiaticus’* (CLas) pathogen. They proposed a model where auxins increase the defense response in *Citrus* against both CLas and its insect vector, through cross talk between the salicylic and jasmonic acid pathways. The WRKY gene families of maize and their Arabidopsis and rice homologs play critical roles in biotic responses to prokaryotic and eukaryotic invasions, including those from insects [50], and specifically corn rootworm [51]. MYB factors are also implicated in abiotic and biotic resistance, including synthesis of metabolites toxic to insects [52], and MYB80 specifically in reactive-oxygen species (ROS) related to environmental stress [53].

Nitric oxide (NO) and ROS are induced upon attack by pathogens and mediate cell-wall cross-linking and the hypersensitive response [54]. Levels of riboflavin are linked with changes in NO [55], and ROS involved in pathogen defense [56]. However, the association of riboflavin with these responses is complex. Although synthesis of riboflavin is required for NO and ROS synthesis [57], down-regulation of free riboflavin induces ROS and pathogen defense [56]. In greenhouse-grown plants in the absence of insect challenge, *RboS-L* expression is over 10-fold higher in Mo17 than in B73 during secondary wall formation (Fig. 5b), and Mo17 stalks show about 3 times less insect damage in field-grown plants (Fig. 3c). Although some of the candidate genes might be false positives, others, including the unknown, P4H, UGE5, RLK-L, IAH, and AGP22-L, have been associated with responses to pathogen stress (Fig. S12).

Our most significant GWAS insect damage location was an unknown protein found to be a member of a family of genes mainly in grass species and not found in dicots by a BLASTp analysis of the entire plant protein sequence collection at NCBI. The closest by sequence rice homolog was found to be upregulated 4-fold when inoculated with the fungal pathogen *Magnaporthe grisea* isolate FR13 in rice leaves [58]. http://systbio.cau.edu.cn/plad/rice_expressionprofile.php?series=GSE7256&group=GSE7256_mock_3d-FR13_3d). The same rice gene was found to be upregulated in rice mutants with an overexpressed calcium-dependent protein kinase that are salt and drought tolerant [59]. Thus, evidence exists for a role in pathogen biotic stress for the unknown gene that could be related to resistance to insect damage. Unlike *RboS-L* and *IAH* gene transcripts, which have a more constitutive expression as part of the larger salicylic acid and jasmonate pathways, the unknown gene may only be expressed at detectable levels during and at the location of the specific stress event.

Any candidate gene or cluster of genes identified by GWAS must eventually be validated by genetic functional analysis, for example, by over-expression or knockdown experiments or mutant analysis. In the absence of these experiments, identification of QTL that are in common with other independent GWAS studies offers some degree of confidence for further study. A meta-analysis across twenty-eight QTL studies in maize revealed 86 potential QTL involved in insect resistance [60]. Subsets of candidate genes were specific to tissue type or to one or more insect species. Ten of the 15 candidate penetration-resistance genes identified by COMPILE match genome locations from the meta-analysis, including our strongest candidate, the gene of unknown function on Chromosome 9 (Table S1).

## Conclusions

The relative speed of data analysis using COMPILE allowed in-depth comparison of population size on trait analysis. Population size and diversity are major constraints for a trait and are not overcome by increasing marker density. COMPILE is customizable and is readily adaptable for application to species with robust, genotyped diversity panels and proteome databases. Even with the constraint of a small population size, novel candidate genes involved in tocopherol synthesis were identified. Application of COMPILE to a much larger maize population found novel candidate genes potentially contributing to flowering time. Using the Goodman AP, novel candidate genes were identified for potential resistance against penetration of the stem by the European corn borer. Analysis of candidate genes exhibiting different expression and levels of resistance both in silico using alternate pathogen systems and comparing inbred lines with varied stem damage gave greater confidence in their effect on corn borer damage. Stem penetration is a marker for one type of resistance to borer damage and identifies different sets of genes from those identified by resistance to pith damage.

## Methods

### Marker set construction and kinship matrix generation

Versions of our GWAS pipeline were generated for both the Goodman AP of 282 maize (*Zea mays***)** inbreds and landraces [9], and the NCRPIS AP of 2815 maize lines [8]. For both the Goodman and NCRPIS APs, fully imputed data from the ZeaGBSv2.7 dataset [61], were converted to B73_RefGen_v4 (henceforth “v4”) coordinates and combined with markers from previous genotyping assays [2, 62–64], as described in Chen and Lipka [14]. All taxa names were standardized to the formats used in the phenotype data of Romay et al. [8], and a Perl script was developed to standardize the phenotype data format used (Dataset S1, Script 1). An additional version of the pipeline for the Goodman AP was generated using the HapMap 3.2.1 dataset (using the final marker set “Goodman 3.2.1”), which consisted of a much denser array of 83 million markers generated by Illumina paired-end sequencing [65]. Fully imputed marker data files using v4 coordinates were downloaded from Panzea (panzea.org) and converted from VCF to HapMap format using a Perl script (Dataset S1, Script 2). All marker sets in this study were filtered to remove sites not anchored to the v4 reference genome, with > 75% missing genotype data, or with minor allele frequency (MAF) < 0.05.

Our Goodman 2.7 dataset contained ~ 310,000 markers, our NCRPIS 2.7 dataset ~ 231,000 markers, and our Goodman 3.2.1 dataset ~ 20.1 million markers. Final HapMap files for each of these three datasets were converted to the numerical format used by GAPIT (maizegenetics.net/gapit [15];) using a Perl script, which conservatively imputed missing data with the major allele (Dataset S1, Script 3), and used to generate per-chromosome kinship matrices. The matrices were created using a separate Perl script (Dataset S1, Script 4) implementing the K_chr model [65], and the Loiselle pairwise kinship algorithm [66].

### Generation of annotation databases

The maize gene list (Gramene 62, ftp.gramene.org) was filtered to contain only the chromosome, start/stop coordinates, and gene symbol for all transcripts and RNA features using a Perl script, which also converted transcript names to their corresponding protein names (Dataset S1, Script 5). A Perl script was used to create a list of maize genes and their midpoint coordinates from the maize gene list (Dataset 1, Script 6), and another Perl script was used to generate a file relating the position of each marker in the dataset to its nearest ten genes from the gene list (Dataset 1, Script 7). To generate a database relating each maize protein to its most sequence-similar protein in rice and Arabidopsis, the maize (B73_RefGen_V4, gramene.org), rice (RAP-DB, rapdb.dna.affrc.go.jp), and Arabidopsis proteomes (Araport 11, arabidopsis.org) were used. These FASTA files were each converted to BLAST databases using the NCBI standalone BLAST+ toolkit [67]. The BLAST+ toolkit and a pair of Perl scripts (Dataset S1, Scripts 8–9) were then used to obtain a database containing, for each maize gene, the gene name, the name and protein description of the closest BLAST matches, and their respective e-value and alignment scores.

### Interpretation of GWAS results

Two methods were used for choosing significant SNPs from the results: 1) Bonferroni threshold [68], where the threshold *p*-value was defined as α/n, where α is the experiment-wise desired *p*-value and n is the number of statistical tests, i.e. the number of markers on a given chromosome, 2) a Benjamini-Hochberg false discovery rate (FDR) correction with a user-specified *p*-value, where each *p*-value was corrected according to the formula Q(i/m), where Q is the specified false discovery rate, i is the rank of the *p*-value (the smallest having rank 1), and m is the total number of tests [69]. Additional Perl scripts assembled the final reports, which contained the name, chromosome, and position of the original marker; the original and Benjamini-Hochberg FDR-corrected *p*-values of the marker; the distance to the midpoint of the closest gene, tRNA, or noncoding RNA; the identity of the genetic feature; the names of rice and Arabidopsis genes closest in sequence to the maize genes; and the description, alignment score, and e-value for each rice and Arabidopsis gene (Dataset S2, Scripts 1–3). For these analyses, significant markers were associated to genes by selecting the nearest 10 genes to the given marker, though the option also exists of specifying an LD window. Scripts were modified to perform the analysis upon the Goodman 2.7 dataset, the NCRPIS 2.7 dataset, and defined regions within the Goodman 3.2.1 and Goodman 2.7 datasets simultaneously (Dataset S2, Scripts 1–3).

### Supplementary scripts and tools

Several additional Perl scripts and tools were developed to streamline the generation of figures and the analysis of GWAS results for this study. The first tool was developed to re-annotate the results according to different available significance thresholds of Bonferroni threshold, Benjamini-Hochberg correction, or manually-defined cutoff, as well as associate genes with significant markers either by LD window or nearest n genes (Dataset S3, Scripts 1). The second tool generates Manhattan plots with accurately placed vertical lines at genes and/or markers of interest for figure generation and annotation purposes (Dataset S3, Script 2). The third tool (Dataset S3, Script 3) aligns cognate Manhattan plots from low- and high-marker-density Goodman results with a customizable display of the genome architecture in a narrowly defined region, showing genes, non-coding RNA features, and exons. The fourth tool (Dataset S3, Script 4) is a generic script for genome architecture visualization easily generalizable for a given species. All scripts are available online on github at https://github.com/mjacksonhill/COMPILE_Hill_et_al._2022_BMC_Plant_Biology.

### Maize Stover collection and sample preparation

Genotypes comprising 274 lines of the Goodman Association Panel (AP) of 282 maize inbreds and landraces, including B73 and Mo17 [9], were originally obtained without restrictions from the Maize Genetics Cooperation Stock Center, at the University of Illinois, Urbana/Champaign (http://maizecoop.cropsci.uiuc.edu/), and grown at the Purdue University Agricultural Center for Research Education. Lines in duplicate were distributed randomly across a rectangular plot, ten plants per 17-ft row with 30-in. spacing between rows and bordered by rows of B73 not included in the sampling. Plants received 200 kg hectare^− 1^ supplemental nitrogen. The lower 70 cm of senescent stems encompassing parts of internodes 3 through 6 from five field-dried plants per line, were harvested and air-dried at 50 °C. After scoring internodes 4 and 5 for the number of larval entry points, they were pooled and ground to 40-mesh in a Model 3 Wiley Mill (Thomas Wiley, Swedesboro, NJ). Milled samples were washed with warm 50% ethanol (v/v in water) and then warm water to remove soluble sugars and other metabolites, suspended in water, flash-frozen in liquid N_2_, and lyophilized.

### Trait analyses

The number of holes generated by the corn borer larvae per unit length of internodes 4 and 5 were recorded as the quantitative trait in each of the stem stover samples for each genotype. The two populations were normalized and assigned a damage index from 1, with zero damage, to 10, in increments of 0.032 holes/cm of internode length, with an average of 0.11 holes/cm of internode length, corresponding to an average index of 3.28. Density, as packed volume of the milled stover after centrifugation, was determined before washing. One to 2 mL of milled stover were placed into pre-weighed 15-mL conical plastic centrifuge tubes (Corning) and the tubes with sample were weighed to the nearest 0.1 mg of sample and centrifuged for 2 min at 2500 rpm (1500 g_max_) in a swinging-bucket rotor. Density was determined as mg mL^− 1^ packed volume.

Cellulose abundance was determined twice in triplicate as described previously [26]. Briefly, 5 ± 0.2 mg of cell wall material was hydrolyzed in 2 M trifluoroacetic acid at 120 °C for 90 min in borosilicate glass vials sealed with Teflon®-lined caps. The pellet was washed three times with water and adjusted to 1 mL for sampling. Cellulose was determined as Glc equivalents by phenol-sulfuric assay [70]. Technical replicates with variance greater than 5% were rerun.

Relative lignin abundance was determined by PyMBMS using a modified protocol from Sykes et al. [71], as described in Penning et al. [72]. Briefly, about 4 mg of freeze-dried cell wall material was added to an 80-μL stainless steel cup and pyrolyzed at 500 °C with a helium flow of 0.9 L min^− 1^ at standard temperature and pressure. Data acquisition time was 90 sec, but product evolution was essentially complete by 30 sec. A Merlin data acquisition system with 17-eV electron ionization was used to gather mass spectral data from *m/z* of 30–450 using a scan rate of 0.5 sec. Total ion counts were compiled from the 60 spectra obtained during the first 30 sec of product evolution. Relative abundance of fragments *m/z* 124, *m/z* 137, *m/z* 138, and *m/z* 151 (G-lignin) and *m/z* 154, *m/z* 167, *m/z* 168, and *m/z* 194 (S-lignin) were used estimate the proportion of total lignin [72].

### RNA isolation and expression analysis

Maize (*Zea mays*) B73 and Mo17 lines were grown at the Purdue University Agricultural Center for Research and Education in West Lafayette, IN, and in campus greenhouses, as described by Penning et al. [23]. Rind tissues were harvested from internodes 2 through 9 of field-grown B73 plants grown for 35 to 63 days, at stages representing secondary cell wall biomass deposited in lower internodes to early and late elongation in upper internodes. A separate collection of rind tissues from B73 and Mo17 internodes 4 through 7, represented the same developmental stages of elongation through secondary wall development in greenhouse-grown plants 49 d after planting [23]. Rind tissues were flash-frozen in liquid N_2_, pulverized by mortar and pestle in liquid N_2_, and RNA extracted from approximately 2 mg of ground tissue in 1 mL of ice-cold TRIzol reagent (Invitrogen, Life Technologies).

Expression analysis was carried out as previously described by Penning et al. [23]. Briefly, cDNA libraries were constructed from pooled total RNA from three biological replicates and clustered on a HiSeq 2000 to produce paired-end 100 base sequences. High-quality trimmed sequences were mapped to the B73_RefGen_v2/3 from PlantGDB (plantgdb.org) using Bowtie2 [73]. Because the high degree of gene duplication in maize, a custom Perl script was used was used to split duplicate reads between the two loci when they mapped together [26]. One count per million or greater was used as a threshold for the detection of transcript [74]. The RNAseq data are available at https://www.ncbi.nlm.nih.gov/sra/PRJNA522448. Expression of specific genes for specific internodes were found using Perl scripts described previously [26]. The averages and standard deviation or variance for each internode were calculated in Excel (microsoft.com).

Additional published data on maize caryopsis and organ expression, and the degree of induction of expression by abiotic and pathogen stress, were obtained from the Maize Gene Expression Atlas at ePlant (bar.utoronto.org).

## Supplementary Information

**Additional file 1: Figure S1.** Association of a long intergenic non-coding RNA among QTL associated with α/γ tocopherol ratio in maize kernels. **Figure S2.** Expression of genes associated with α-tocopherol synthesis in developing caryopses. **Figure S3.** Expression profiles of genes associated with days-to-silking of genes identified by GWAS. **Figure S4.** Expression profiles of genes associated with days to silking identified by COMPILE. **Figure S5.** Heat map for larval penetrance in holes per cm internode length across field range and row. **Figure S6**. Quantile-Quantile plots for larval damage index in the Goodman AP population. **Figure S7**. Scatter plots comparing European corn borer damage index to cellulose abundance, G- and S-lignin abundance, and milled stover density. **Figure S8.** Hydropathy plots of maize and other grass proteins encoded by genes homologous to the candidate maize gene associated with resistance to larval penetrance. **Figure S9.** FOCUS plots of candidate genes among QTL associated with European Corn Borer damage in maize stalks. **Figure S10.** Expression profiles of genes associated with larval penetration with significant internode expression. **Figure S11.** Expression profiles of genes associated with larval penetration with little or no internode expression. **Figure S12.** Expression profiles of genes associated with larval penetration that are induced by abiotic and pathogen stress. **Figure S13.** Manhattan plots showing GWAS results for the 20Mbp Chromosome 8 region using both low- and high-density markers. **Table S1.** QTL associated with resistance to European corn borer penetration in maize stems compared with a meta-analysis of QTL associated with multiple stem and insect resistance and storage pests.

**Additional file 2: Dataset S1.** Scripts 1-9, **Dataset S2.** Scripts 1-3. **Dataset S3.** Scripts 1-4.

## Data Availability

All scripts used to generate COMPILE are in the three datasets included in this published article and its supplementary information files. The RNAseq data are available at NCBI with the following link: https://www.ncbi.nlm.nih.gov/sra/PRJNA522448. Complete scripts for COMPILE are available online on github at https://github.com/mjacksonhill/COMPILE_Hill_et_al._2022_BMC_Plant_Biology.

## Abbreviations

AGP22-L: Arabinogalactan-protein22-like
AP: Goodman Association Panel
CCT1: CONSTANS C-terminus-like transcriptional co-factor
CHAT: choline *O*-acetyltransferase
CHUP1: Chloroplast unusual positioning1
CLas pathogen: *Candidatus Liberibacter asiaticus* pathogen
DET1: De-Etiolated1
DUF: Domain of unknown function
GDA: Guanosine Diphosphatase
GWAS: Genome-Wide Association Studies
IAH: IAA-Ala Amino Hydrolase
IBA: Indole butyric acid
IPA: Indole propionic acid
LOD score: Logarithm of the odds score
MLM: Mixed Linear Model
MYB: Proto-oncogene1-like transcription factor
NAM: Nested association mapping
NCRPIS: North Central Regional Plant Introduction Station
NO: nitric oxide
OHP3: One-helix protein gene
P4H: Proline-4-hydroxylase
PEBP: Phosphatidylethanolamine-binding
PyMBMS: Pyrolysis Molecular Beam-Mass Spectrometry
QTL: Quantitative trait locus (loci)
RAP2.7: Apetala2-like transcription factor
RboS-L: Riboflavin synthase-like
RLK: Receptor-like kinase
RraA: Ribonuclease Regulating Protein
ROS: Reactive oxygen species
UGE5: UDP-Glu:UDP-Gal 4-epimerase
WRKY: WRKY sequence-containing transcription factor
