# Supplementary material for: COMPILE: a GWAS computational pipeline for gene discovery in complex genomes
Source: BMC Plant Biol. 2022 Jul 2;22:315. doi: 10.1186/s12870-022-03668-9 (PMC9250234; doi:10.1186/s12870-022-03668-9)
Supplement: Supplementary file 1 — Additional file 1: Figure S1. Association of a long intergenic non-coding RNA among QTL associated with α/γ tocopherol ratio in maize kernels. Figure S2. Expression of genes associated with α-tocopherol synthesis in developing caryopses. Figure S3. Expression profiles of genes associated with days-to-silking of genes identified by GWAS. Figure S4. Expression profiles of genes associated with days to silking identified by COMPILE. Figure S5. Heat map for larval penetrance in holes per cm internode length across field range and row. Figure S6. Quantile-Quantile plots for larval damage index in the Goodman AP population. Figure S7. Scatter plots comparing European corn borer damage index to cellulose abundance, G- and S-lignin abundance, and milled stover density. Figure S8. Hydropathy plots of maize and other grass proteins encoded by genes homologous to the candidate maize gene associated with resistance to larval penetrance. Figure S9. FOCUS plots of candidate genes among QTL associated with European Corn Borer damage in maize stalks. Figure S10. Expression profiles of genes associated with larval penetration with significant internode expression. Figure S11. Expression profiles of genes associated with larval penetration with little or no internode expression. Figure S12. Expression profiles of genes associated with larval penetration that are induced by abiotic and pathogen stress. Figure S13. Manhattan plots showing GWAS results for the 20Mbp Chromosome 8 region using both low- and high-density markers. Table S1. QTL associated with resistance to European corn borer penetration in maize stems compared with a meta-analysis of QTL associated with multiple stem and insect resistance and storage pests. [file 12870_2022_3668_MOESM1_ESM.pdf]

Supplemental Material

Title: COMPILE: A GWAS computational pipeline for gene discovery in complex genomes

Authors: Matthew J. Hill[*,1], Bryan W. Penning[†], Maureen C. McCann[‡,§], Nicholas C. Carpita[*,‡]

[*]Department of Botany and Plant Pathology, Purdue University, West Lafayette, Indiana 47907 USA
[†]USDA-ARS Corn, Soybean and Wheat Quality Research Unit, Wooster, Ohio 44691 USA
[‡]Department of Biological Sciences, Purdue University, West Lafayette, Indiana 49707 USA
[§]Purdue Center for Plant Biology, Purdue University, West Lafayette, Indiana 47907 USA

Present address:
[1] Whitehead Institute for Biomedical Research, 455 Main Street, Cambridge, MA 02142, USA, and the Department of Biology, Massachusetts Institute of Technology, Cambridge, MA 02139, USA

Supplemental Datasets:
SD1. Scripts used to generate databases within COMPILE
SD2. COMPILE and FOCUS programs
SD3. Accessory scripts useful for data visualization

Table S1. QTL associated with resistance to European corn borer penetration in maize stems compared with a meta-analysis of QTL associated with multiple stem and insect resistance and storage pests.

					QTL for insect resistance from meta-analysis[1]			
Chrom	Gene Number	Name	Position (Mbp)	MLM *p*-value	Bin	Range	Markers (Mbp)	Type
1	Zm00001d032079	RboS-L	211.80	3.67E-05	1.07	210.52-219.19	idp4855-rz698a(ppy)	SIR
1	Zm00001d034084	WRKY31	283.65	4.84E-05	1.10-11	279.67-291.89	idp2395-umc2514	LIR
1	Zm00001d034085	DUF1336	283.65	4.84E-05	1.10-11	279.67-291.89	idp2395-umc2514	LIR
2	Zm00001d001813	RLK-L	1.41	6.11E-06	(None)			
2	Zm00001d002991	MYB-L	28.96	4.46E-05	2.03-04	27.74-32.51	bnlg381-ay103944	SIR
2	Zm00001d002992	DUF3755	28.96	4.46E-05	2.03-04	27.74-32.51	bnlg381-ay103944	SIR
2	Zm00001d004120	P4H	84.08	7.44E-05	(None)			
2	Zm00001d007788	CHUP1	239.76	5.45E-06	(None)			
3	Zm00001d043701	IAH	207.87	4.36E-05	3.08-09	206.61-218.85	umc231-idp8203	LIR
5	Zm00001d012838	GDA1-L	0.98	2.63E-05	(None)			
7	Zm00001d020093	UGE5	91.47	4.37E-05	7.02	40.24-109.48	idp8247-tidp3642	SIR
8	Zm00001d011256	OHP3	144.70	9.44E-06	8.05-06	141.95-160.44	umc1287-pza03182	KIR
8	Zm00001d011257	AGP22-L	144.70	9.44E-06	8.05-06	141.95-160.44	umc1287-pza03182	KIR
9	Zm00001d045360	Unknown	19.55	2.67E-06	9.02-03	16.97-26.90	umc1037-w11	LIR
10	Zm00001d023332	WRKY63	3.60	6.09E-05	(None)			

[1]Meta-analysis of QTL of associated responses to stem borers and storage pests feeding on leaves (LIR), stems (SIR), and kernels (KIR) of maize from diverse genetic and geographical background (Badji et al., 2018).

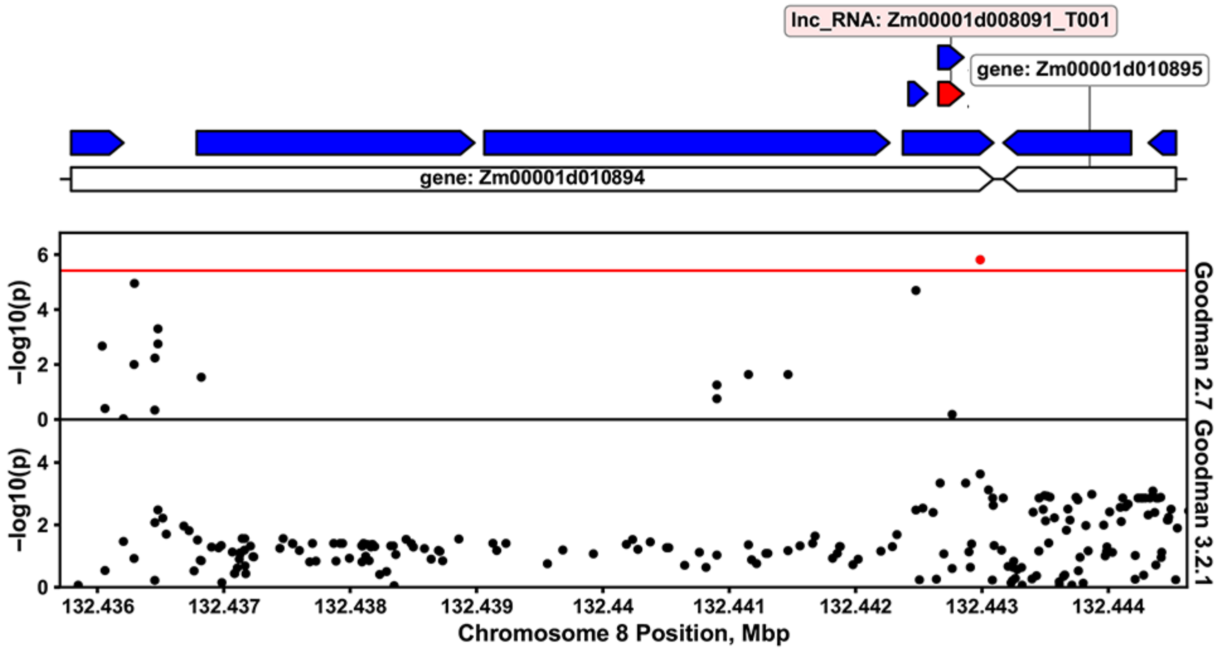

Figure S1 Association of a long intergenic non-coding RNA among QTL associated with α/γ tocopherol ratio in maize kernels. Negative $\log_{10}$ *p*-values are plotted against physical position (B73_RefGen_v4). Genome architecture is indicated above the Manhattan plots, with genes shown in white, exons shown in blue, and non-coding RNA features shown in red. The upper Manhattan plot shows FOCUS results with the sparse 2.7 markers; the lower Manhattan plot shows results with dense 3.2.1 markers. The red horizontal line indicates the Bonferroni significance threshold for Chromosome 8 at alpha = 0.1 (not visible for the Goodman 3.2.1 results). The lincRNA resides in a *cis*-NAT pair of a homolog (Zm00001d010894) to rice *Embryonic flower1* (*EMF1*) gene involved in floral development and a gene of unknown function encoding a DUF 659 (Zm00001d010895). Data from Chen and Lipka *et al.* [14].

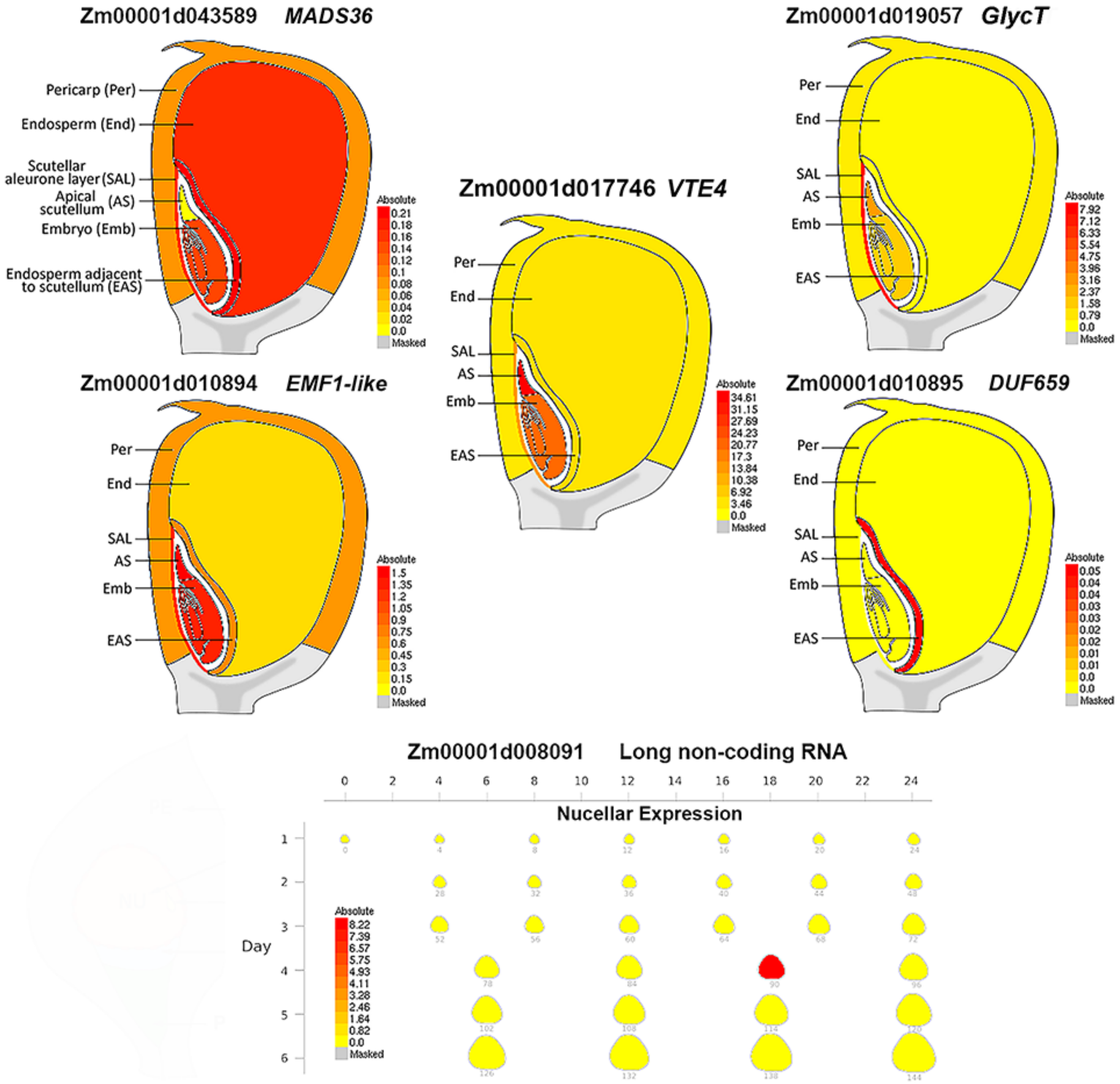

Figure S2 Expression of genes associated with α-tocopherol synthesis in developing caryopses. Comparison plots adapted from visualization tools at ePlant (bar.utoronto.ca; from the data of Doll *et al.* [17]). Caryopsis maps of 13-day-old of maize B73 were generated from transcripts extracted from dissection of the sub-compartments. Maps of nucellar expression from 0 to 6 days post-pollination were from the data of Yi *et al.* [18].

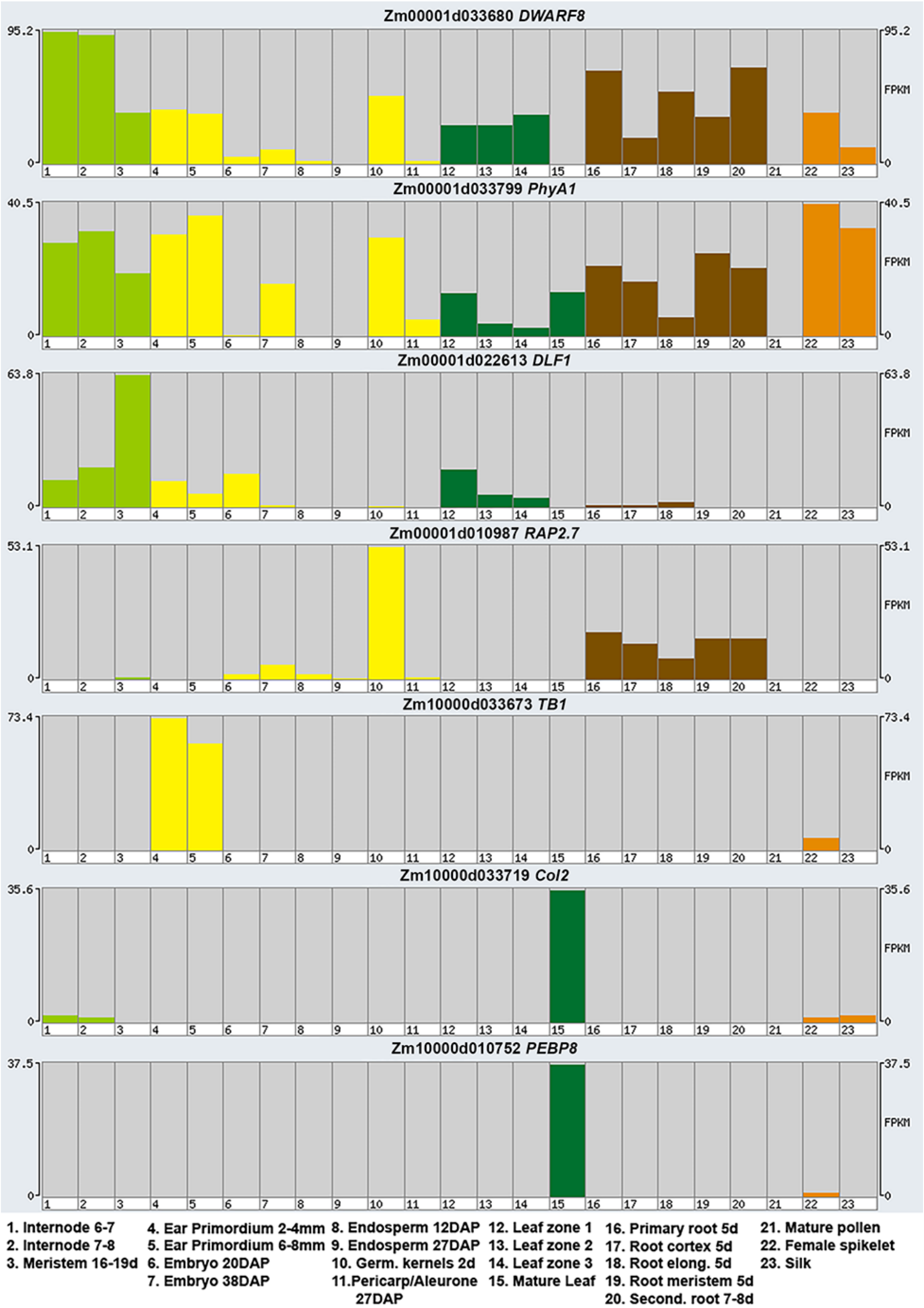

Figure S3 Expression profiles of genes associated with days-to-silking of genes identified by GWAS. Genes identified by Romay et al. [8]. Comparison plots of B73 tissue expression were generated by the visualization tools at ePlant (bar.utoronto.ca) from the data of Stelpflug *et al.* [21] and Hoopes *et al.* [22].

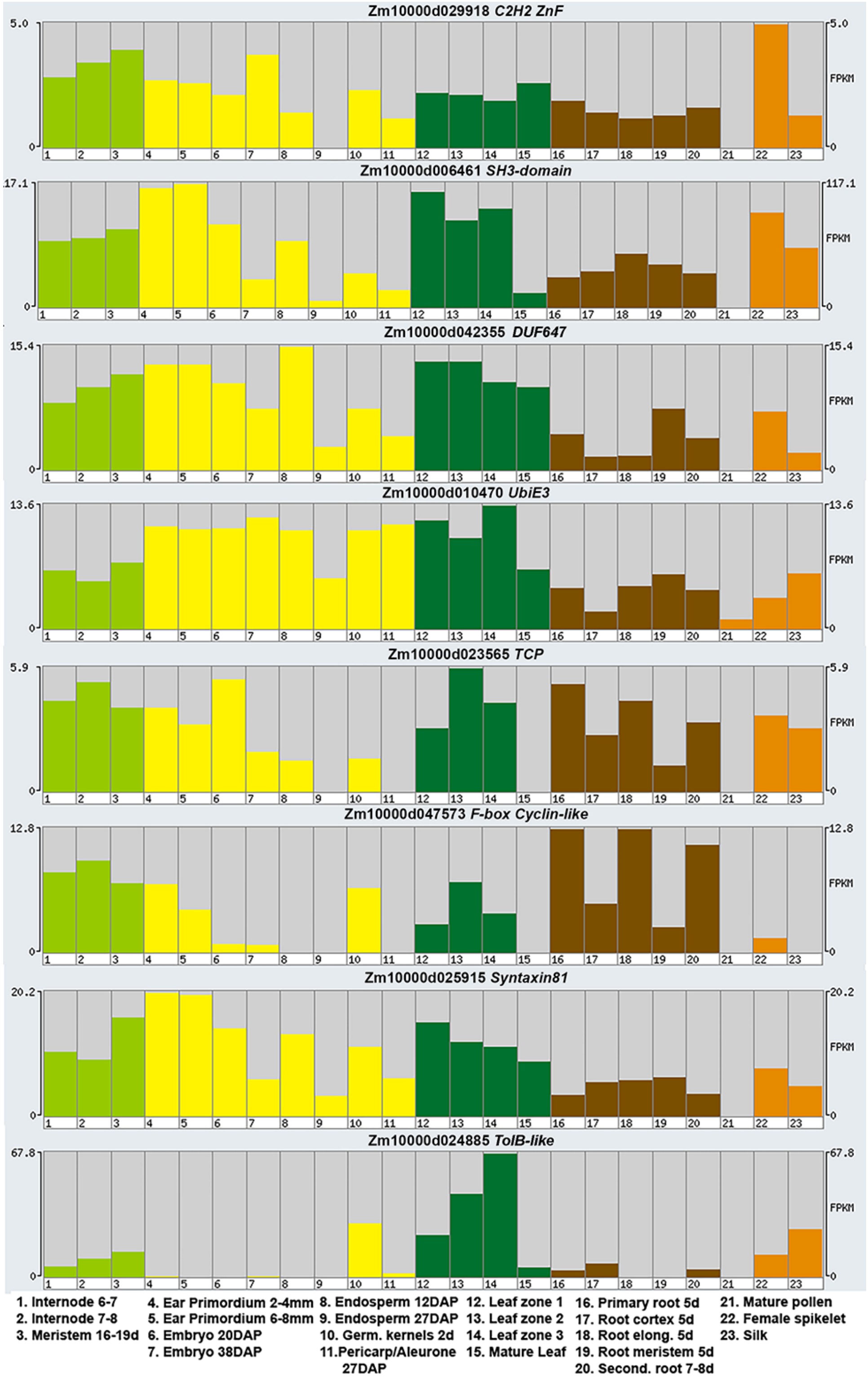

Figure S4 Expression profiles of genes associated with days to silking by COMPILE. Genes identified by COMPILE are in addition to those identified by Romay *et al.* [8] in Figure S3. Comparison plots of B73 tissue expression were generated by the visualization tools at ePlant (bar.utoronto.ca) from the data of Stelpflug *et al.* [21] and Hoopes *et al.* [22].

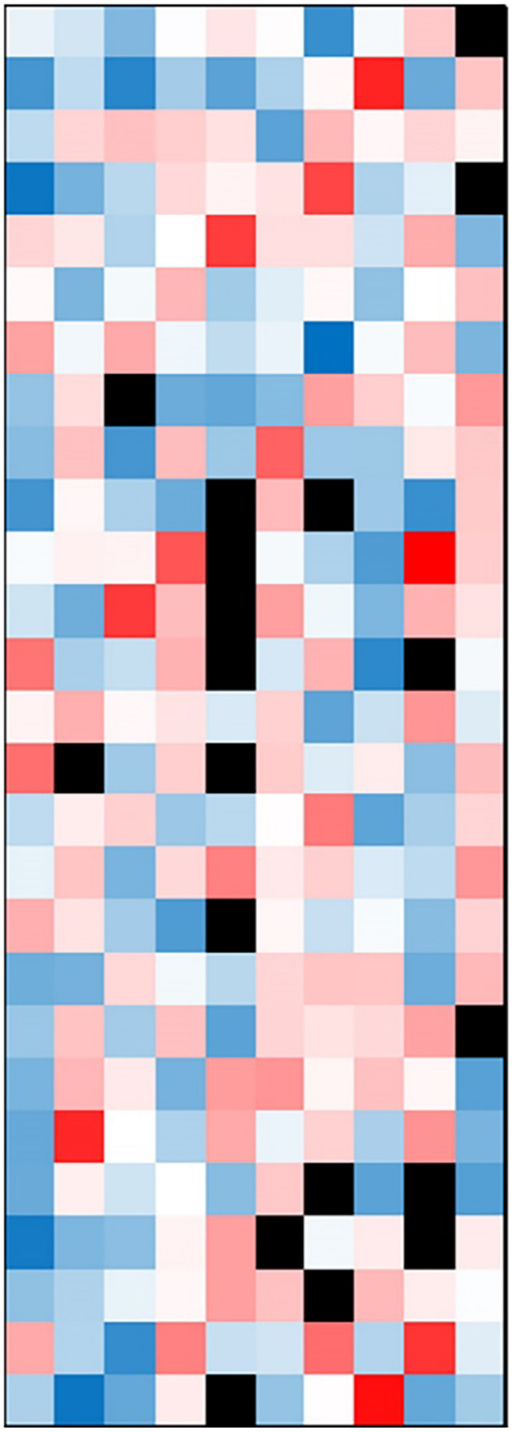

Figure S5 Heat map for larval penetrance in holes per cm internode length across field range and row. Damage indicated in a gradient from blue (low) to white (median) to red (high). Black squares indicate missing data.

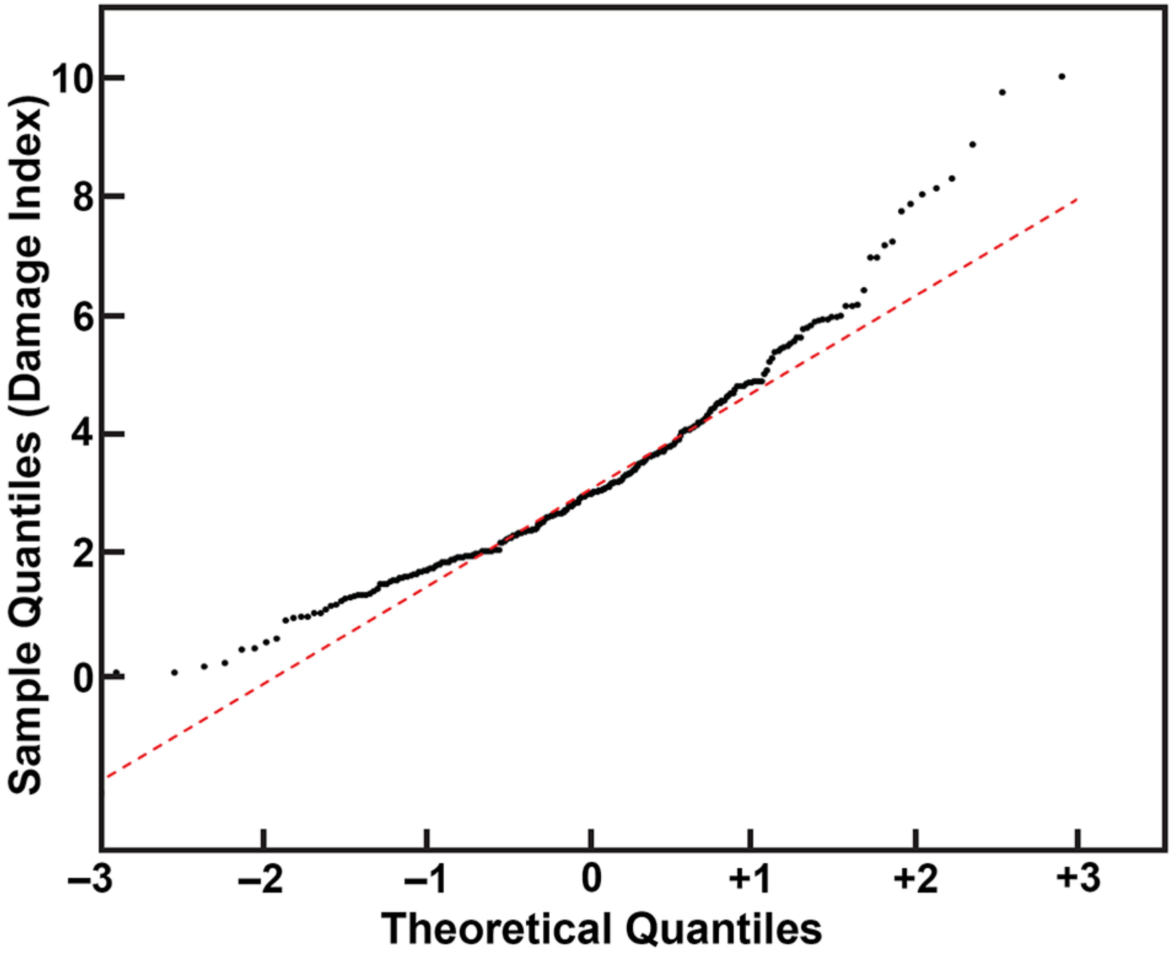

Figure S6 Quantile-Quantile plots for larval damage index in the Goodman AP population. The theoretical quantiles of a normal distribution (X-axis) are plotted against the actual quantiles of the data set (Y-axis). The red dashed line connects the 25th and 75th percentiles of the theoretical and sample distributions.

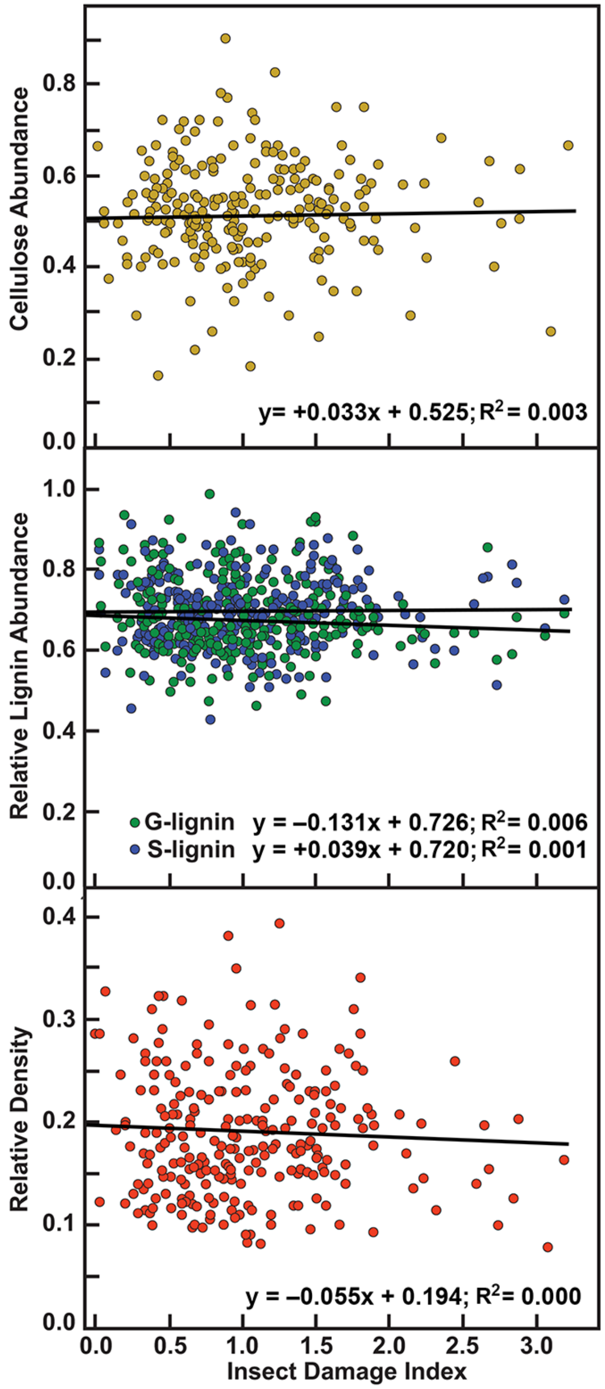

Figure S7 Scatter plots comparing European corn borer damage index to cellulose abundance, G- and S-lignin abundance, and milled stover density. Pearson's correlation coefficients (R^2) and linear regressions are shown for traits between individual lines across the field-grown Goodman AP.

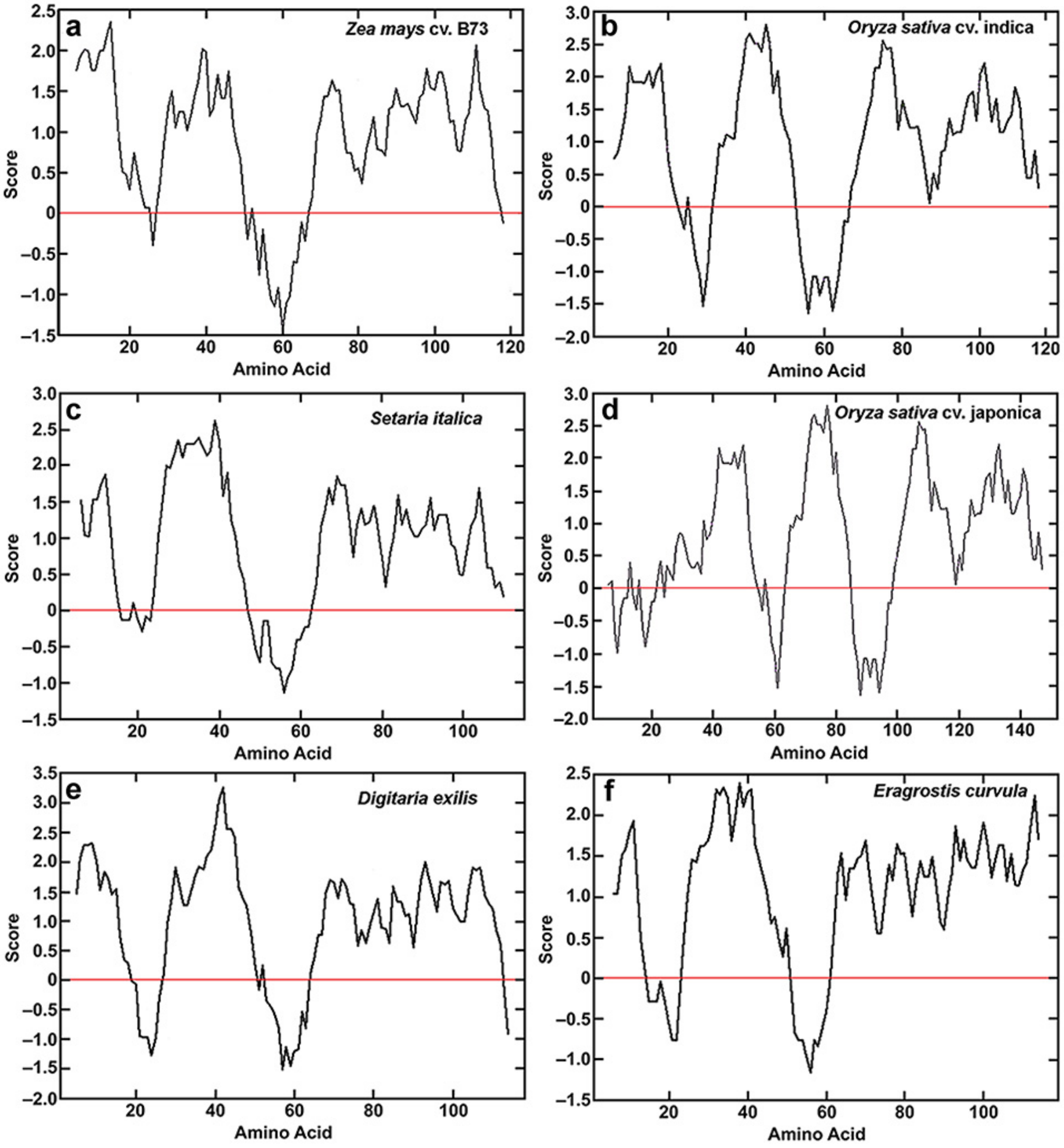

Figure S8 Hydropathy plots of maize and other grass proteins encoded by genes homologous to the candidate maize gene associated with resistance to larval penetrance. The maize sequences are compared to those of other grass species with closest in sequence. Kyte-Doolittle plots were generated using 11-amino acid averaging (https://web.expasy.org/protscale/). **a**. Maize (*Zea mays* cv. B73; PWZ05131.1). **b.** Rice (*Oryza sativa* cv. indica; EEC79995.1). **c.** Foxtail millet (*Setaria italica*; XP_004964557.1). **d**. (*Oryza sativa* cv. Japonica; XP_015642145.1) Unknown protein sequence. **e.** Fonio millet (*Digitaria exilis*; CAB3469495.1) **f.** Weeping lovegrass (*Eragrostis curvula*; TVU01005.1)

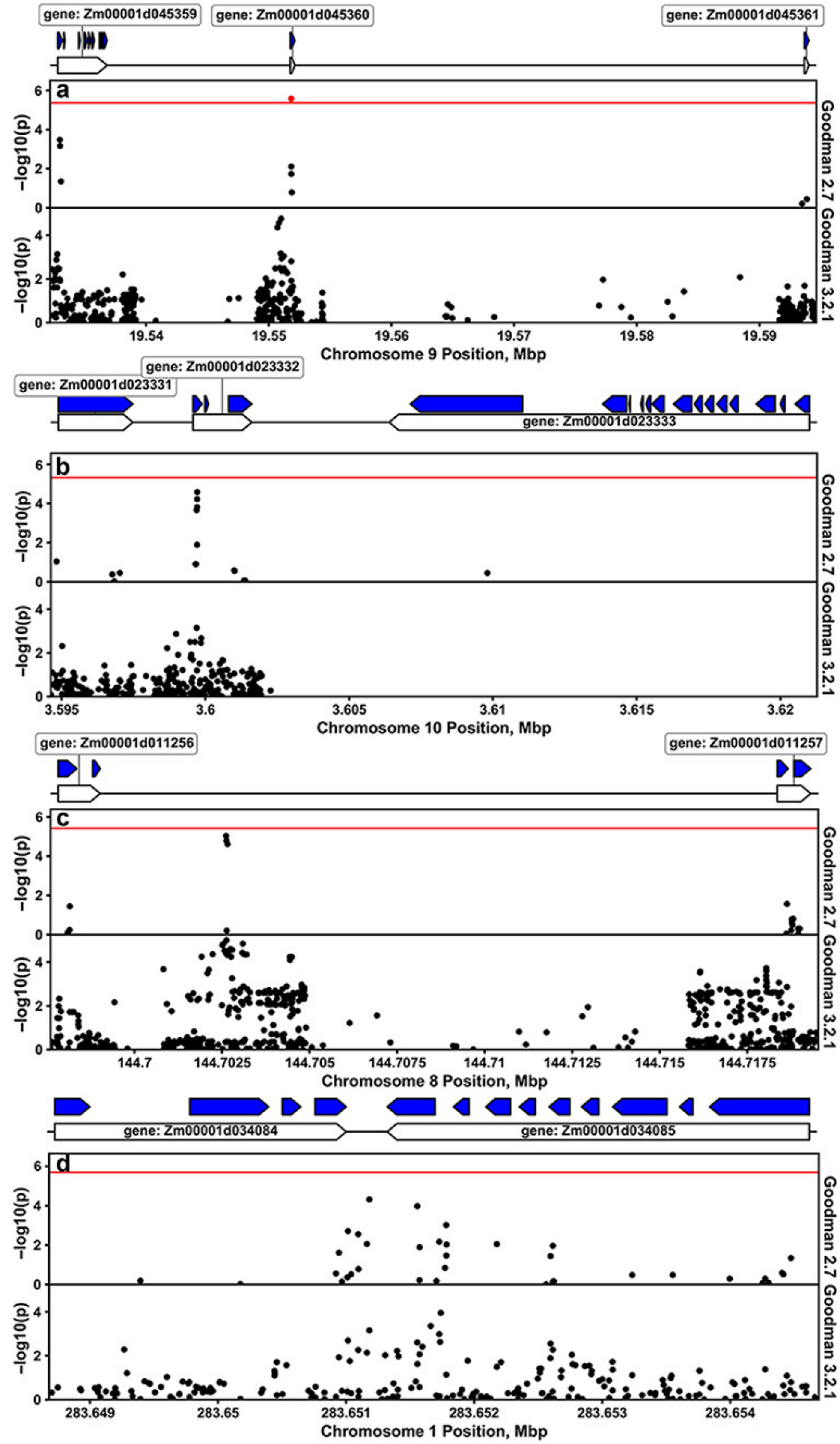

Figure S9 [Legend on next page]

Figure S9 FOCUS plots of candidate genes among QTL associated with European Corn Borer damage in maize stalks. Negative $\log_{10}$ *p*-values are plotted against physical position (B73_RefGen_v4). Genome architecture is indicated atop the Manhattan plots, with genes shown in white and exons shown in blue. The top Manhattan plot panel shows FOCUS results with the sparse 2.7 markers; bottom Manhattan plot panel shows results with dense 3.2.1 markers. The red horizontal line indicates the Bonferroni significance threshold at alpha = 0.1 for the Goodman 2.7 results. **a**. Significant markers associated with a gene of unknown function (Zm00001d045360) on Chromosome 9. **b**. Significant markers associated with *WRKY63* gene (Zm00001d023332) on Chromosome 10. **c**. Significant markers associated with the intervening sequences of a *One-helix Protein3* (*OHP3*) gene (Zm00001d011256) and distal from an *Arabinogalactan22-like* (*AGP22-like*) gene (Zm00001d011257) on Chromosome 8. **d**. Significant markers associated with the *C*-termini and intergenic sequences of a *cis*-NAT pair of a WRKY 31 (Zm00001d034084) and a DUF 1336-containing protein (Zm00001d034085) on Chromosome 1.

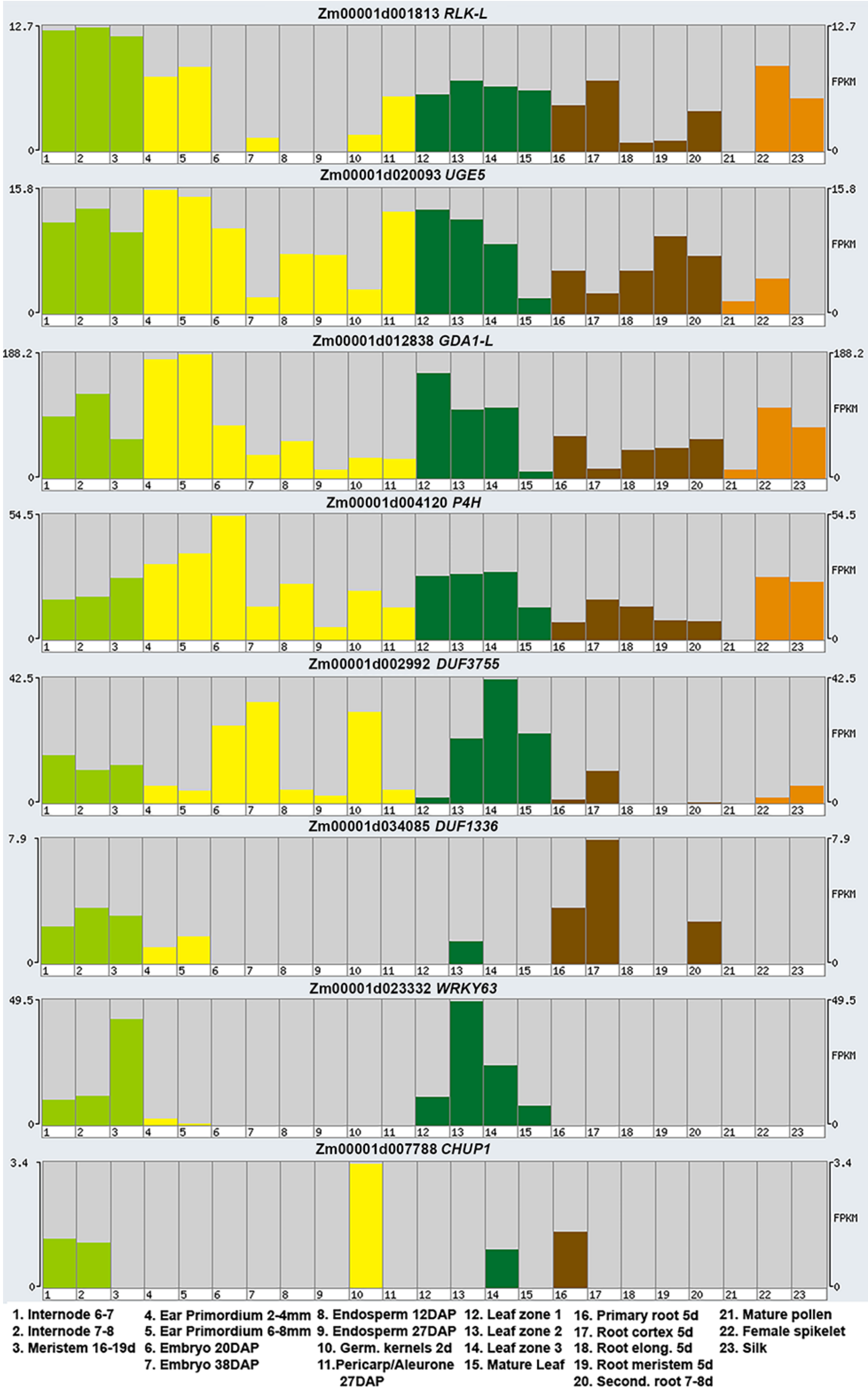

Figure S10 Expression profiles of genes associated with larval penetration with significant internode expression. Comparison plots of B73 tissue expression were generated by the visualization tools at ePlant (bar.utoronto.ca) from the data of Stelpflug *et al.* [21] and Hoopes *et al.* [22].

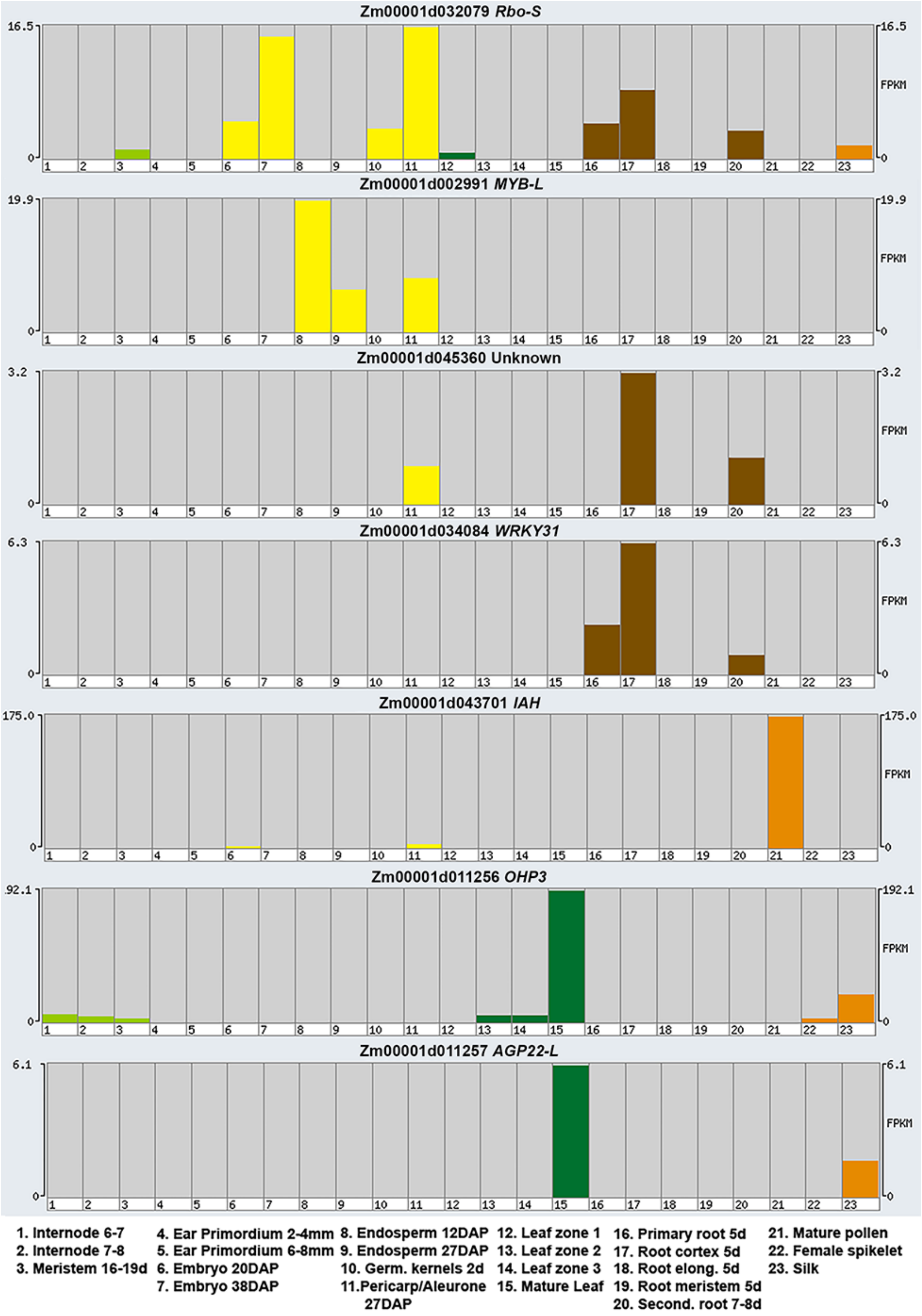

Figure S11 Expression profiles of genes associated with larval penetration with little or no internode expression. Comparison plots of B73 tissue expression were generated by the visualization tools at ePlant (bar.utoronto.ca) from the data of Stelpflug *et al.* [21] and Hoopes *et al.* [22].

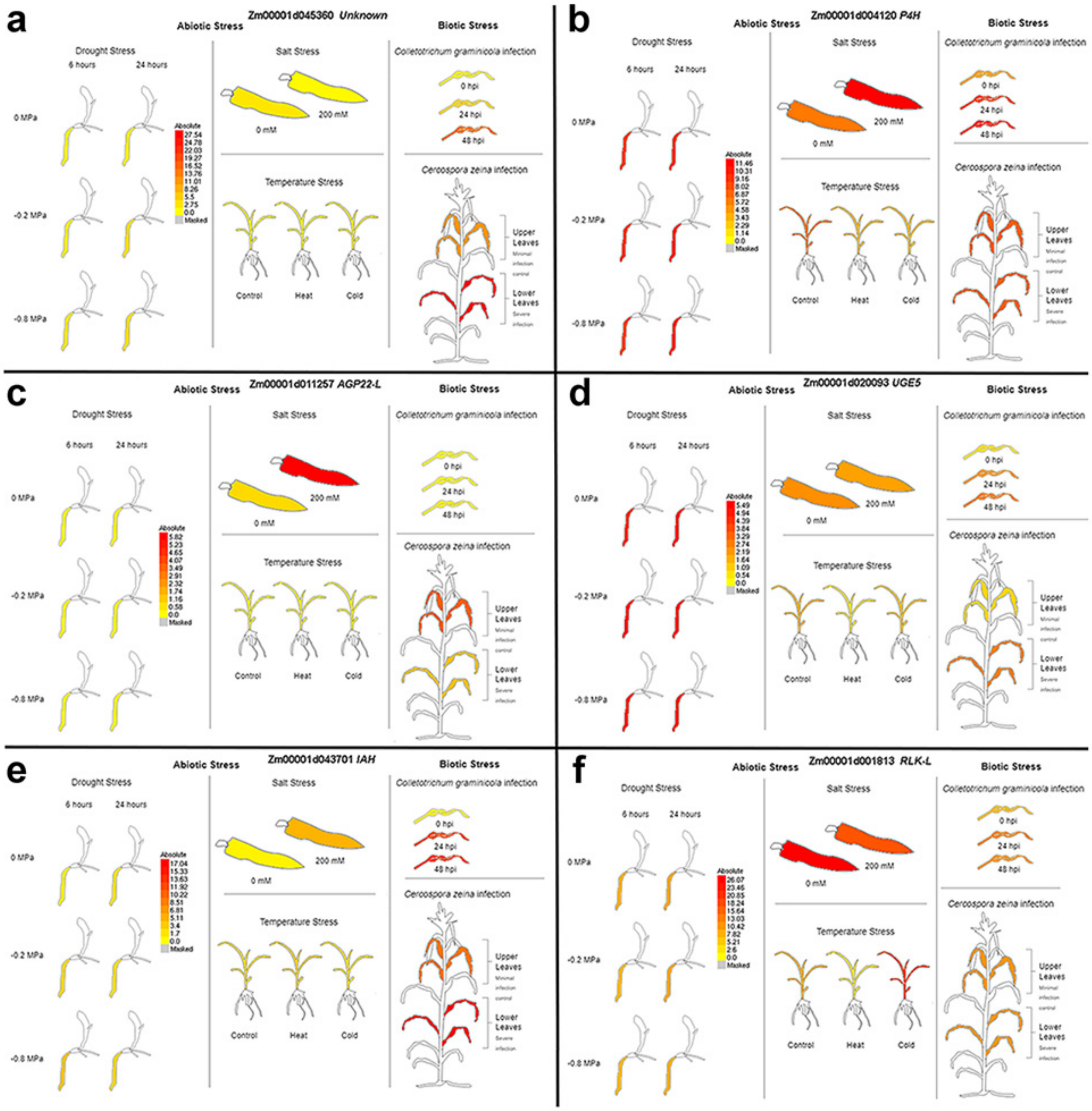

Figure S12 Expression profiles of genes associated with larval penetration that are induced by abiotic and pathogen stress. Comparison plots of B73 organ expression were generated by the visualization tools at ePlant (bar.utoronto.ca) from the data of responses to abiotic and biotic stress as contributed by Opitz *et al.* (2014), Makarevitch *et al.* (2015), and Hoopes *et al.* (2019). **a**. Unknown (Zm00001d045360). **b**. *P4H* (Zm00001d004120). **c**. *AGP22-L* (Zm00001d011257). **d**. UGE5 (Zm00001d020093). **e**. *IAH* (Zm00001d043701). **f**. *RLK-L* (Zm00001d001813).

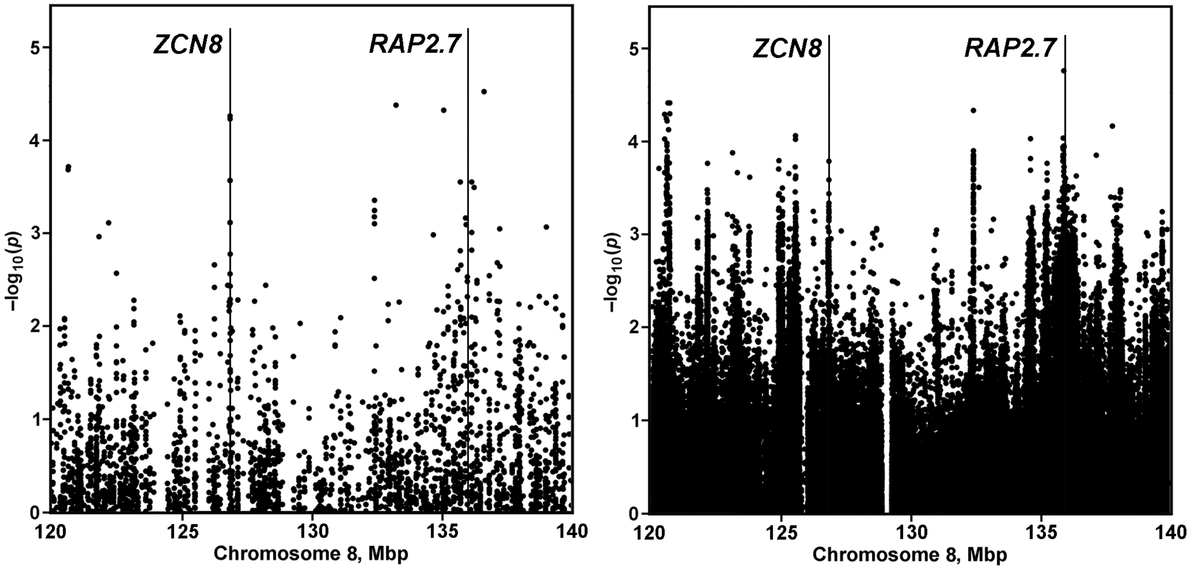

Figure S13 Manhattan plots showing GWAS results for the 20Mbp Chromosome 8 region containing the *PEBP* (*ZCN8*) and *RAP2.7* loci. Data from Lipka *et al.* [14] were input, specifying the region on Chromosome 8 between 120 and 140Mbp. Negative $\log_{10}$ *p*-values are plotted against physical position (B73_RefGen_v4). The left plot displays the results of the analysis using the sparse Goodman 2.7 marker set, while the right plot displays the results of the analysis using the dense Goodman 3.2.1 marker set. The positions of the two genes are indicated with vertical lines.
