# Supplementary material for: COMPILE: a GWAS computational pipeline for gene discovery in complex genomes
Source: BMC Plant Biol. 2022 Jul 2;22:315. doi: 10.1186/s12870-022-03668-9 (PMC9250234; doi:10.1186/s12870-022-03668-9)
Supplement: Supplementary file 2 — Additional file 2: Dataset S1. Scripts 1-9, Dataset S2. Scripts 1-3. Dataset S3. Scripts 1-4. [file 12870_2022_3668_MOESM2_ESM.pdf]

Dataset 1, Script 1

```
#standardize maize phenotype data names to those of Romay et al., 2013

#STRUCTURE
#SD1/
#├── SD1-S1.pl #script to perform renaming
#├── Input X.txt file(s) #phenotype data with tab-separated data with
genotype names in first column
#├── Output X-named.txt file(s) #renamed phenotype data
#└── SD1-Files/
#    └── Romay.txt #contains standardized line names according to
Romay et al. 2013

use strict;
use warnings;

open my $Romay, '<', 'SD1-Files/Romay.txt';
chomp(my @Romay = <$Romay>);
close $Romay;
my $total = $#Romay+1;

my @files = glob "*.txt";
for my $file (@files) {

      my %queries;

      open my $in, '<', "$file";
      my $header = <$in>;

      while (<$in>) {

            #certain renames specific to these studies
            chomp $_;
            my @terms = split("\t", $_);
            $terms[0] =~ s/:.*?$//;
            $terms[0] =~ s/B2-good/B2/;
            $terms[0] =~ s/Il677A/Il677a/;
            $terms[0] =~ s/Ky226/KY226/;
            $terms[0] =~ s/Ky228/KY228/;
            $terms[0] =~ s/MO17/Mo17/;
            $terms[0] =~ s/Mo1W/MO1W/;
            $terms[0] =~ s/OH43/Oh43/;
            $terms[0] =~ s/PA91/Pa91/;
            $terms[0] =~ s/TX601/Tx601/;
            $terms[0] =~ s/TzI10/Tzi10/;
            $terms[0] =~ s/TzI18/Tzi18/;
            $terms[0] =~ s/TzI8/Tzi8/;
            $terms[0] =~ s/Va102/VA102/;
```

```
        $terms[0] =~ s/VA26/Va26/;
        $terms[0] =~ s/W117HT/W117Ht/;
        $terms[0] =~ s/WF9/Wf9/;
        $terms[0] =~ s/W22R-r-rstd/W22R-r-std/;
        $terms[0] =~ s/W22R-r-std_CS-2909-1/W22R-r-std/;
        $terms[0] =~ s/trisacum/tripsacum/;
        $queries{$terms[0]} = $terms[1];

    }

    close $in;

    open my $out, '>', "$file-named.txt";
    print $out $header;

    my $i = 0;

    for my $reference (@Romay) {

        my $data = $queries{$reference} // "NaN";
        if ($data eq "NaN") { $i++; }
        print $out "$reference\t$data\n";

    }

    close $out;
    print "Missing taxa were $i of $total for $file\n"

}
```

Dataset 1, Script 2
#convert all VCF files in script folder to HapMap files

#lines 78 and 79 refer to specific taxa for these studies; to edit the entire VCF file without changing the contents:
#change the range of positions in line 77 to read (0..n-1), where n is the total number of taxa
#change the list of names in line 78 to a double-quoted, comma-separated, ordered list of the taxa in the VCF file
#total number of files to convert is specified on line 63 (e.g. for one VCF file per chromosome)
#line 118 filters marker data based on missing and minor allele proportions; edit or comment out this line as needed

#STRUCTURE
#SD1/
#├── SD1-S2.pl #script to perform renaming
#├── Input .vcf file(s) named 1.vcf, 2.vcf, etc.
#└── Output .hmp.txt file(s)

```
use strict;
use warnings;

my %IUPAC = (
	'AC' => 'M',
	'CA' => 'M',
	'AG' => 'R',
	'GA' => 'R',
	'AT' => 'W',
	'TA' => 'W',
	'CG' => 'S',
	'GC' => 'S',
	'CT' => 'Y',
	'TC' => 'Y',
	'GT' => 'K',
	'TG' => 'K',
	'-A' => '0',
	'A-' => '0',
	'-C' => '0',
	'C-' => '0',
	'-G' => '0',
	'G-' => '0',
	'-T' => '0',
	'T-' => '0',
	'+A' => '0',
	'A+' => '0',
	'+C' => '0',
	'C+' => '0',
```

```
      '+G' => '0',
      'G+' => '0',
      '+T' => '0',
      'T+' => '0',
      '+-' => '0',
      '-+' => '0',
      'AN' => 'N',
      'NA' => 'N',
      'CN' => 'N',
      'NC' => 'N',
      'GN' => 'N',
      'NG' => 'N',
      'TN' => 'N',
      'NT' => 'N',
      '+N' => 'N',
      'N+' => 'N',
      '-N' => 'N',
      'N-' => 'N',
);

for my $i (1..10) {

open my $in, '<', "$i.vcf";
open my $out, '>', "$i.hmp.txt";

while (<$in>) {

      if ($_ =~ m/^#[^#]/) {

            last;

      }

}

my @positions =
(23,916,917,918,919,920,921,922,123,923,924,925,926,927,928,929,930,93
1,932,933,934,935,936,937,938,939,940,941,942,943,944,945,946,947,948,
949,950,951,952,953,954,955,956,957,958,146,959,960,961,962,963,971,96
4,965,966,967,968,969,970,972,973,974,975,976,977,212,978,979,980,981,
982,983,218,984,985,986,987,988,989,990,991,992,993,994,995,996,997,99
8,999,1000,1001,224,1002,1003,1004,1005,1006,228,1007,1008,1009,1010,1
011,1012,1013,1014,1015,1016,1017,1018,1019,1020,1021,1022,1023,1024,1
025,1026,1027,1028,1029,1030,1031,1032,1033,1034,1035,1036,1037,1049,1
038,1040,1041,1042,1043,1044,1045,1046,1047,1048,1039,1050,1051,1052,1
053,1054,1055,1056,1057,407,1058,1059,1060,1061,1062,1063,1064,430,461
,462,1065,1066,1067,1068,1069,1070,1071,1072,1073,1074,1075,1076,1077,
1078,1079,1080,517,1081,1082,1083,523,1084,1085,1086,1087,1088,1089,10
```

```
90,1091,1092,1093,1094,1095,1096,1097,1098,1099,1100,1101,1102,1103,11
04,1105,1106,1107,1108,1109,1110,1111,1112,1113,1114,1115,1116,1117,11
18,1119,1120,1121,1122,1123,1124,1125,1126,1127,1128,1129,541,1130,113
1,1132,1133,1134,1135,1136,1137,1138,1139,1140,1141,1142,1143,1144,114
5,1146,1147,1148,1149,1150,1151,1152,1153,1154,1155,1156,1157,1158,115
9,1160,1161,1162,1163,1164,1165,1166,1167,1168,1169,1170,1171,1172,117
3,1174,1175,1176,725,732,1177,1178);
my @reference_taxa = ("207","33-16","38-
11","4226","4722","A188","A214N","A239","A272","A441-
5","A554","A556","A6","A619","A632","A634","A635","A641","A654","A659"
,"A661","A679","A680","A682","Ab28A","B10","B103","B104","B105","B109"
,"B14A","B164","B2","B37","B46","B52","B57","B64","B68","B73","B75","B
76","B77","B79","B84","B97","C103","C123","C49A","CH701-
30","CH9","CI.7","CI187-
2","CI21E","CI28A","CI31A","CI3A","CI64","CI66","CI90C","CI91B","CM105
","CM37","CM7","CML10","CML103","CML108","CML11","CML14","CML154Q","CM
L157Q","CML158Q","CML206","CML218","CML220","CML228","CML238","CML247"
,"CML254","CML258","CML261","CML264","CML277","CML281","CML287","CML31
1","CML314","CML321","CML322","CML323","CML328","CML330","CML331","CML
332","CML333","CML341","CML38","CML418","CML45","CML5","CML52","CML61"
,"CML69","CML77","CML91","CML92","CMV3","CO106","CO125","CO255","D940Y
","DE_2","DE1","DE811","E2558W","EP1","F2834T","F6","F7","GA209","GT11
2","H105W","H49","H84","H91","H95","H99","Hi27","HP301","Hy","I137TN",
"I205","I29","IA2132","Ia5125","IDS28","IDS69","IDS91","Il101","Il14H"
,"Il677a","K148","K4","K55","K64","Ki11","Ki14","Ki2021","Ki21","Ki3",
"Ki43","Ki44","Ky21","KY226","KY228","L317","L578","LH132","LH74","LH8
2","M14","M162W","M37W","MEF156-55-
2","Mo17","Mo18W","MO1W","Mo24W","Mo44","Mo45","Mo46","Mo47","MoG","Mp
339","MS1334","MS153","MS71","Mt42","N192","N28Ht","N6","NC222","NC230
","NC232","NC236","NC238","NC250","NC258","NC260","NC262","NC264","NC2
90A","NC294","NC296","NC296A","NC298","NC300","NC302","NC304","NC306",
"NC310","NC314","NC318","NC320","NC324","NC326","NC328","NC33","NC336"
,"NC338","NC340","NC342","NC344","NC346","NC348","NC350","NC352","NC35
4","NC356","NC358","NC360","NC362","NC364","NC366","NC368","ND246","Oh
40B","Oh43","Oh43E","Oh603","OH7B","Os420","P39","Pa762","Pa875","Pa88
0","Pa91","R168","R177","R229","R4","SA24","SC213R","SC357","SC55","SD
40","SD44","Sg1533","Sg18","T232","T234","T8","Tx303","Tx601","Tzi10",
"Tzi11","Tzi16","Tzi18","Tzi25","Tzi8","Tzi9","U267Y","VA102","Va14","
Va17","Va22","Va26","Va35","Va59","Va85","Va99","VaW6","W117Ht","W153R
","W182B","W22","W64A","Wf9","Yu796_NS");

my $hmp_taxa = join("\t", @reference_taxa);
print $out
"rs#\talleles\tchrom\tpos\tstrand\tassembly#\tcenter\tprotLSID\tassayL
SID\tpanelLSID\tQCcode\t$hmp_taxa\n";

while (<$in>) {
```

```
	chomp $_;
	my @fields = split("\t", $_);

	my $chrom = $fields[0];
	my $pos = $fields[1];
	my $id = $fields[2];
	my $ref_allele = $fields[3];

	my $alt_allele_field = $fields[4];
	$alt_allele_field =~ s/<INS>/\+/;
	$alt_allele_field =~ s/<DEL>/-/;
	my @alt_alleles = split(',', $alt_allele_field);
	my $alt1 = $alt_alleles[0];
	my $alt2 = $alt_alleles[1] // undef;
	my $alt3 = $alt_alleles[2] // undef;
	my $alleles = join('/', $ref_allele, @alt_alleles);

	@fields = @fields[9..$#fields];

	my @data;
	for (@positions) {

		push (@data, $fields[$_]);

	}

	my $data_field = join("\t", @data[0..$#data]);
	$data_field =~ s|\./\.|N|g;

	my $missing = () = $data_field =~ m|\tN\t|g;
	my $minor = () = $data_field =~ m`(/1|1/|/2|2/|3/|/3)`g;

	if (($missing > 69) || (($minor/(558-(2*$missing)) < 0.05))) {
next; }

	$data_field =~ s`:.*?(\t|\n)`$1`g;
	$data_field =~ s|:.*?$||g;
	$data_field =~ s|0/0|$ref_allele|g;
	$data_field =~ s|1/1|$alt1|g;

	my $zeroone = $IUPAC{"$ref_allele"."$alt1"};
	$data_field =~ s`(0/1|1/0)`$zeroone`g;

	if (defined($alt2)) {

		$data_field =~ s|2/2|$alt2|g;
		my $zerotwo = $IUPAC{"$ref_allele"."$alt2"};
		my $onetwo = $IUPAC{"$alt1"."$alt2"};
```

```
            $data_field =~ s`(0/2|2/0)`$zerotwo`g;
            $data_field =~ s`(1/2|2/1)`$onetwo`g;

      }

      if (defined($alt3)) {

            $data_field =~ s|3/3|$alt3|g;
            my $zerothree = $IUPAC{"$ref_allele"."$alt3"};
            my $onethree = $IUPAC{"$alt1"."$alt3"};
            my $threetwo = $IUPAC{"$alt3"."$alt2"};
            $data_field =~ s`(0/3|3/0)`$zerothree`g;
            $data_field =~ s`(1/3|3/1)`$onethree`g;
            $data_field =~ s`(3/2|2/3)`$threetwo`g;

      }

      print $out
"$id\t$alleles\t$chrom\t$pos\t+\tNA\tNA\tNA\tNA\tNA\tNA\t$data_field\n
";

}

close $in;
close $out;

}
```

Dataset 1, Script 3
#convert HapMap files to GAPIT numerical format files-- does not precisely follow rules as to reference alleles, but this is irrelevant for GWAS purposes.

#chromosome number specified on line 15
#missing data is imputed as the major allele at line 50; change as desired. GAPIT requires all data to be imputed, so options are major allele, heterozygote, or minor allele

```
#STRUCTURE
#SD1/
#├── SD1-S3.pl #script to perform conversion
#├── Output .GM.txt and .GD.txt file(s)
#└── Input 1.hmp.txt - ##.hmp.txt genotype files

use warnings;
use strict;

for my $i (1..10) {

      open my $infile, '<', "$i.hmp.txt";

      my %numerical;
      open my $GM, '>', "$i.GM.txt";
      print $GM "Name\tChromosome\tPosition\n";
      open my $GD, '>', "$i.GD.txt";

      my $header = <$infile>;
      my @taxa = split("\t", $header);
      @taxa = @taxa[11..$#taxa];
      chomp @taxa;

      my @markerids;

      while (<$infile>) {

            my @data = split("\t", $_);
            chomp @data;

            my $marker_id = $data[0];
            push @markerids, $marker_id;

            my @alleles = split('/', $data[1]);
            my $major_allele = $alleles[0]; #Major allele is ALWAYS
leftmost allele in HapMap file
```

```
            unless ($major_allele =~ m/[ACTG+-]/) { print
"$major_allele\t$."; }
            my $position = $data[3];
            print $GM "$marker_id\t$i\t$position\n";

            @data = @data[11..$#data];

            for my $h (0..$#data) {

                  if ($data[$h] =~ m/[N$major_allele]/ ) { push @{
$numerical{$taxa[$h]} }, '2'; } #Conservatively impute missing data to
major allele; use 2 for major allele
                  elsif ($data[$h] =~ m/[KMRSWY0]/ ) { push @{
$numerical{$taxa[$h]} }, '1'; } #Heterozygotes are 1
                  elsif ($data[$h] =~ m/[ACTG+-]/ ) { push @{
$numerical{$taxa[$h]} }, '0'; } #Homozygotes that don't match major
allele (i.e. minor alleles) are 0

            }

      }

      close $GM;

      my $markerlist = join("\t", @markerids);
      print $GD "taxa\t$markerlist\n";

      for my $g (0..$#taxa) {

            my $output = join("\t",
$taxa[$g],@{$numerical{$taxa[$g]}});
            print $GD "$output\n";

      }

      close $GD;

}
```

Dataset 1, Script 4
#second of two scripts to generate sequence similarity databases between query and target proteomes, preceded by SD1-S9.pl
#requires the NCBI BLAST+ toolkit, BLAST databases formatted by said toolkit, and the gene names column from GeneNameList.gff3 (product of SD1-S5.pl) formatted as a standalone file

#target species specified line 31

```
#STRUCTURE
#SD1/
#├── SD1-S8.pl #first script to run to generate database
#├── SD1-S9.pl #second script to run to generate database
#├── GeneNameList.gff3 input file (product of SD1-S5.pl)
#├── SPECIES.txt #output file(s) for sequence similarity databases
between query and target species
#└── SD1-Files/
#    └── BLAST/
#      ├── Various .exe and .dll files used in the NCBI BLAST+ toolkit
#      ├── format_dbs.pl #run to use the NCBI BLAST+ toolkit to format
new databases. Folder structure is DATABASES/SPECIES/SPECIES(.fasta)
#           └── DATABASES/
#           └── folders for each species containing the FASTA-
formatted proteome for that species, named identically to the folder
and species within format_dbs.pl, this script, and SD1-S9.pl

use strict;
use warnings;
use Cwd qw(abs_path);
use File::Copy;
use File::Path;
use File::Basename;
use List::Util qw(sum);

my $type = fileparse_set_fstype("Unix"); #Allows spaces in file paths
my $dirname = dirname(__FILE__);
my $top = defined($dirname) ? $dirname : '.';

my @sp = ('Arabidopsis','Rice');
for my $species (@sp) {#Match Maize Query Genes to Rice/Arabidopsis
BLAST Matches

      my %unique;
      open my $in, '<', "$top/Blasted$species.txt";
      open my $out, '>', "$top/$species.txt";
      print $out "Query\t$species Gene\tDescription\tBLAST Alignment
Score\tE-Value\n";
```

```
	while (<$in>) {

			my @fields = split("\t",$_);
			my $gene = $fields[0];

			unless (exists($unique{$gene})) {

			$unique{$gene} = 1;
			$fields[1] =~ s/LOC_//;
			$fields[2] =~ s/%2C/,/; #Fix errors in BLAST
descriptions from HTML-encoded characters
			$fields[2] =~ s/%26#64257%3B/fi/;
			$fields[2] =~ s/%26/&/;
			$fields[2] =~ s/%3B/;/;
			$fields[2] =~ s/\| (.*?)\|.*/$1/g;
			$fields[2] =~ s/protein\|//;
			my $description = $fields[2];

			my $outline =
join("\t",$fields[0],$fields[1],$description,$fields[3],$fields[4]);
			chomp $outline;
			print $out "$outline\n";

		}

	}

	close $in;
	close $out;

}
```

Dataset 1, Script 5
#convert a gene list GFF3 file to the trimmed format used by COMPILE

#for these studies, converts maize transcript names to gene names on line 42 by removing _T suffix
#feature IDs to retain specified on lines 32 and 38
#input file name specified on line 16

#STRUCTURE
#SD1/
#├── SD1-S5.pl #script to run to perform GFF cleaning
#├── input .gff3 file
#└── GeneNameList.gff3 output file

```
use warnings;
use strict;

open my $input, '<', 'Zea_mays.B73_RefGen_v4.45.gff3'; #change filename as necessary
open my $output, '>', "GeneNameList.gff3";

while (<$input>) {

	next unless($_ !~ /^#/);

	chomp $_;
	my @t = split("\t", $_);
	my $chromosome = $t[0];
	my $source = $t[1];
	my $type = $t[2];
	my $start = $t[3];
	my $stop = $t[4];
	my $id = $t[8];

	if (($type =~ m/snRNA|snoRNA|lnc_RNA|tRNA|pre_miRNA|rRNA|miRNA/) && ($chromosome =~ m/^[[1-9]|10]$/)) { #specifies feature types to save, and assumes 10 chromosomes

		$id =~ m/gene:(.*?);/;
		my $symbol = $1;
		print $output "$chromosome\t$type\t$start\t$stop\t$symbol\n";

	} elsif (($type =~ m/mRNA/) && ($chromosome =~ m/^[[1-9]|10]$/)) {

		$id =~ m/transcript:(.*?);/;
		my $symbol = $1;
```

```
          $symbol =~ s/_T.*?$//;
          print $output
"$chromosome\tgene\t$start\t$stop\t$symbol\n";

     }

}

close $input;
close $output;
```

Dataset 1, Script 6
#convert the GeneNameList.gff3 file used by COMPILE (from SD1-S5.pl) into a list of gene positions for further use with SD1-S7.pl

#maize gene name format specified in regular expression on line 30

#STRUCTURE
#SD1/
#├── SD1-S6.pl #script to run to generate gene position list
#├── GeneNameList.gff3 input file (product of SD1-S5.pl)
#└── GenePositions.txt output file

```
use strict;
use warnings;

open my $in, '<', 'GeneNameList.gff3';
open my $out, '>', 'GenePositions.txt';

my %HoA;
my %Chromosomes;

while (<$in>) {

      chomp $_;
      my @columns = split("\t", $_);

      my $chromosome = $columns[0];
      my $start = $columns[2];
      my $stop = $columns[3];
      my $name = $columns[4];

      $name =~ s/(Zm.*?)_.{4}/$1/;
      push @{ $HoA{$name} }, $start, $stop;
      $Chromosomes{$name} = $chromosome;

}

close $in;
my @genes = keys %HoA;
my @outputs;

for my $gene (@genes) {

      my @positions = @{$HoA{$gene}};
      @positions = sort { $a <=> $b } @positions;

      my $chromosome = $Chromosomes{$gene};
      my $first = shift @positions;
```

```
      my $last = pop @positions;
      my $average = ($first+$last)/2;

      push @outputs, "$chromosome\t$average\t$gene\n";

}

my @sorted = sort {
    my ($aa, $bb) = map { (split /\t/)[0] } $a, $b;
      my ($cc, $dd) = map { (split /\t/)[1] } $a, $b;
    $aa <=> $bb
           or
      $cc <=> $dd;
} @outputs;


for my $line (@sorted) {

      print $out $line;

}

close $out;
```

Dataset 1, Script 7
#identify the ten nearest genes (from marker .gm.txt files in GAPIT numerical format to each specified gene in GenePositions.txtmarker set
#requires the marker GM files used by GAPIT (product of SD1-S3.pl) and GenePositions.txt (product of SD1-S6.pl)

#number of nearest genes specified on lines 48, 52
#number of chromosomes specified on line 32

#STRUCTURE
#SD1/
#├── SD1-S7.pl #script to run after SD1-S6.pl to generate list of ten nearest genes to each marker
#├── Input 1.GM.txt - ##.GM.txt #genotype files for panel in GAPIT numerical format (e.g. from SD1-S3.pl)
#├── GenePositions.txt file from SD1-S6.pl
#└── Nearest.txt output file

```
use strict;
use warnings;
use Number::Closest;

my @AoH;
open my $translate, '<', 'GenePositions.txt';

while (<$translate>) {

      chomp $_;
      my @columns = split("\t", $_);
      $AoH[$columns[0]]{$columns[1]} = $columns[2];

}

close $translate;
open my $output, '>', "Nearest.txt";

for my $j (1..10) {

      open my $markers, '<', "$j.GM.txt";
      my $header = <$markers>;

      my @coords = keys(%{$AoH[$j]});
      @coords = sort { $a <=> $b } @coords;

      while (<$markers>) {

            chomp $_;
            my @columns = split("\t", $_);
```

```
            my $name = $columns[0];
            my $position = $columns[2];

            my $finder = Number::Closest->new(number => $position,
numbers => \@coords) ;
            my @values = @{$finder->find(10)};
            @values = sort @values;

            my @temp;
            for my $k (0..9) {

                  push @temp, $AoH[$j]{$values[$k]};

            }

            my $out = join("\t", @temp);
            my $outline = "$name\t$out";
            print $output "$outline\n";

      }

      close $markers;


}

close $output;
```

Dataset 1, Script 8
#first of two scripts to generate sequence similarity databases between query and target proteomes, followed by SD1-S9.pl
#requires the NCBI BLAST+ toolkit, BLAST databases formatted by said toolkit, and the gene names column from GeneNameList.gff3 (product of SD1-S5.pl) formatted as a standalone file

#threshold for a valid match (i.e. value beyond which no match will be output to the final database) is an e-value of 1E-20 (specified in the '-evalue' parameters in lines 75/76 of SD1-S9.pl)
#if the best match value is above 1E-20, no match will be described. For more permissive matching, set the threshold higher in lines 75 and 76.

#query species specified line 51, and in FILES/BLAST/format_dbs.pl (when adding new databases)
#target species specified line 78, and in FILES/BLAST/format_dbs.pl (when adding new databases)
#name filtering options (specific to maize for this study) set on lines 58, 59

```
#STRUCTURE
#SD1/
#├── SD1-S8.pl #first script to run to generate database
#├── SD1-S9.pl #second script to run to generate database
#├── GeneNameList.gff3 input file (product of SD1-S5.pl)
#├── BlastedSPECIES.txt #output file(s) for sequence similarity databases between query and target species
#└── SD1-Files/
#    └── BLAST/
#      ├── .exe and .dll files used in the NCBI BLAST+ toolkit
#      ├── format_dbs.pl #run to use the NCBI BLAST+ toolkit to format new databases. Folder structure is DATABASES/SPECIES/SPECIES(.fasta)
#           └── DATABASES/
#           └── folders for each species containing the FASTA-formatted proteome for that species, named identically to the folder and species within format_dbs.pl, this script, and SD1-S9.pl
```

```perl
use strict;
use warnings;
use Cwd qw(abs_path);
use File::Copy;
use File::Path;
use File::Basename;
use List::Util qw(sum);

my $type = fileparse_set_fstype("Unix"); #Allows spaces in file paths
my $dirname = dirname(__FILE__);
```

```
my $top = defined($dirname) ? $dirname : '.';

my @Hits;

open my $gff, '<', "$top/GeneNameList.gff3";
while (<$gff>) {

	chomp $_;
	my @GFFline = split("\t",$_);
	my $name=$GFFline[4];
	push @Hits, $name;

}

close $gff;

system("$top/SD1-Files/BLAST/blastdbcmd.exe -db $top/SD1-
Files/BLAST/DATABASES/Maize/Formatted_Maize -dbtype prot -entry_batch
$top/Names.txt -out $top/NamesRaw.txt");

open my $in, '<', "$top/NamesRaw.txt"; #Removes everything but the
name from the ID line
open my $out, '>', "$top/NamesClean.txt";

while (<$in>) {

	my $linein = substr($_,0,1);
	my $newline = substr($_,1,14);

	if ($linein eq '>') {

		print $out '>'."$newline\n";

	} else {

		print $out $_;

	}
}

close $in;
close $out;

my @targetpecies = ("Maize","Rice");
for my $taxon (@targetspecies) {

	system("$top/SD1-Files/BLAST/blastp.exe -db $top/SD1-
Files/BLAST/DATABASES/$taxon/Formatted_$taxon -query
```

```
$top/NamesClean.txt -evalue 0.0000000000000000001 -out
$top/Blasted$taxon.txt -outfmt \"6 qseqid sseqid stitle bitscore
evalue\"");

}
```

Dataset 1, Script 9
#second of two scripts to generate sequence similarity databases between query and target proteomes, preceded by SD1-S9.pl
#requires the NCBI BLAST+ toolkit, BLAST databases formatted by said toolkit, and the gene names column from GeneNameList.gff3 (product of SD1-S5.pl) formatted as a standalone file

#target species specified line 31

```
#STRUCTURE
#SD1/
#├── SD1-S8.pl #first script to run to generate database
#├── SD1-S9.pl #second script to run to generate database
#├── GeneNameList.gff3 input file (product of SD1-S5.pl)
#├── SPECIES.txt #output file(s) for sequence similarity databases between query and target species
#└── SD1-Files/
#    └── BLAST/
#      ├── Various .exe and .dll files used in the NCBI BLAST+ toolkit
#      ├── format_dbs.pl #run to use the NCBI BLAST+ toolkit to format new databases. Folder structure is DATABASES/SPECIES/SPECIES(.fasta)
#           └── DATABASES/
#           └── folders for each species containing the FASTA-formatted proteome for that species, named identically to the folder and species within format_dbs.pl, this script, and SD1-S9.pl
```

```
use strict;
use warnings;
use Cwd qw(abs_path);
use File::Copy;
use File::Path;
use File::Basename;
use List::Util qw(sum);

my $type = fileparse_set_fstype("Unix"); #Allows spaces in file paths
my $dirname = dirname(__FILE__);
my $top = defined($dirname) ? $dirname : '.';

my @sp = ('Arabidopsis','Rice');
for my $species (@sp) {#Match Maize Query Genes to Rice/Arabidopsis BLAST Matches

      my %unique;
      open my $in, '<', "$top/Blasted$species.txt";
      open my $out, '>', "$top/$species.txt";
      print $out "Query\t$species Gene\tDescription\tBLAST Alignment Score\tE-Value\n";
```

```
	while (<$in>) {

		my @fields = split("\t",$_);
		my $gene = $fields[0];

		unless (exists($unique{$gene})) {

		$unique{$gene} = 1;
		$fields[1] =~ s/LOC_//;
		$fields[2] =~ s/%2C/,/; #Fix errors in BLAST
descriptions from HTML-encoded characters
		$fields[2] =~ s/%26#64257%3B/fi/;
		$fields[2] =~ s/%26/&/;
		$fields[2] =~ s/%3B/;/;
		$fields[2] =~ s/\| (.*?)\|.*/$1/g;
		$fields[2] =~ s/protein\|//;
		my $description = $fields[2];

		my $outline =
join("\t",$fields[0],$fields[1],$description,$fields[3],$fields[4]);
		chomp $outline;
		print $out "$outline\n";

	    }

	}

	close $in;
	close $out;

}
```

Dataset 2, Script 1

```
#configuration options start at line 38 for:
#    threshold options for selecting candidate significant markers. 
which of these options to use is defined at lines 484-485 and 571-573
#    method for assigning markers to genes-- specified LD window or n 
closest genes to marker

#more in-depth customization:
#    GAPIT model defined at lines 343, 381, together with surrounding 
script. For example, enable compression by setting the group.from 
parameter to 0, and/or by adding a group.to parameter to adjust the 
groupings.

#STRUCTURE
#SD2/GOODMAN_2.7/
#├── SD2-S1.pl #script to execute COMPILE
#├── input *.txt file(s) containing phenotype data
#├── RUNS/
#|    ├── Example.txt #contains example input data in correct format
#|    └── RESULTS_FOLDERS #one per input data file
#|         ├── INTERMEDIATES/ #raw GAPIT output files, including some 
not used by COMPILE (e.g. reports of marker effect size)
#|         ├── output *_DATA.txt #copy of original input phenotype 
data
#|         ├── output *_Manhattan.pdf #Manhattan plots of results
#|         ├── output *_QQ.pdf #quantile-quantile plot of results
#|         ├── output *_Arabidopsis.txt #candidate gene report 
matching maize to arabidopsis genes
#|         └── output *_Rice.txt #candidate gene report matching 
maize to rice genes
#└── COREFILES/
#     ├── GAPIT/ #contains files used by GAPIT to execute GWAS
#     └── GENOME/
#          ├── 1.GD.txt - 10.GD.txt, 1.GM.txt - 10.GM.txt #marker 
data in GAPIT numerical format (SD1-S2, SD1-S3)
#          ├── 1k.txt - 10k.txt #kinship matrix data (SD1-S4)
#          ├── GeneNameList.gff3 #filtered gene info list (SD1-S5)
#          ├── GenePositions.txt #list of gene positions (SD1-S6)
#          ├── KnownGenes.txt #list of known genes in maize
#          ├── taxa.txt #list of taxa
#          ├── Nearest.txt #atlas of nearest ten genes to each marker 
(SD1-S7)
#          └── Rice.txt, Arabidopsis.txt #sequence similarity 
databases relating maize to rice and arabidopsis (SD1-S8, SD1-S9)

use strict;
use warnings;
use Cwd qw(abs_path);
```

```
use File::Copy;
use File::Path;
use File::Basename;
use List::Util qw(sum);
use POSIX qw(ceil);

my $debug = 0; #If 1, doesn't delete temporary files or directories
my $do_serial = 0; #If 1, runs chromosomes in order 1-10 instead of in
parallel
my $bjh_alpha = 0.1; #Significance threshold for BJH FDR p-val
my $bonf_alpha = 0.1; #Significance threshold for Bonferroni alpha
my $p_annotate = 0.0001; #Significance threshold to annotate markers
anyway

my $use_ld = 1; #whether to use number of close genes or LD window.
$number_of_close_genes option will still function, e.g. if >1 gene
within the LD window, so set appropriately.
my $number_of_close_genes = 10; #number of genes near each marker to
annotate (max 10)
my $ld_window = 10000; #window in bp around each marker to find genes
to annotate

my $type = fileparse_set_fstype("Unix"); #Allows spaces in file paths
my $dirname = dirname(__FILE__);
my $top = defined($dirname) ? $dirname : '.';

my @num_markers;
for my $i (1..10) { #Get numbers of markers on each chromosome

     open my $file, '<', "$top/COREFILES/GENOME/$i.GM.txt";
     while (<$file>) {}
     my $line = $.-1; #0-index takes care of -1 for empty line at end;
another -1 for header
     push @num_markers, $line;
     close $file;

}

my @bonferroni;
my @pcalc;
for my $nm (@num_markers) {

     push @pcalc, $bonf_alpha/$nm; #Y-values for significance
thresholds on plot
     my $x = -(log($bonf_alpha/$nm)/log(10));
     push @bonferroni, $x;

}
```

```
my $avg_p = sum(@pcalc)/@pcalc;
my $avg_Y = -(log($avg_p)/log(10)); #Average Bonferroni significance
threshold across all 10 chromosomes

my $b1 = $bonferroni[0]; #Because R doesn't accept the array value; I
know it looks bad
my $b2 = $bonferroni[1];
my $b3 = $bonferroni[2];
my $b4 = $bonferroni[3];
my $b5 = $bonferroni[4];
my $b6 = $bonferroni[5];
my $b7 = $bonferroni[6];
my $b8 = $bonferroni[7];
my $b9 = $bonferroni[8];
my $b10 = $bonferroni[9];

print "Initializing, please wait a moment...\n";

my %nonprot;
open my $gff, '<', "$top/COREFILES/GENOME/GeneNameList.gff3";
while (<$gff>) {

      chomp $_;
      my @columns = split("\t", $_);
      if ($columns[1] ne 'gene') { $nonprot{$columns[4]} = $columns[1];
}

}

close $gff;

my %start;
my %stop;
if ($use_ld == 1) {

      open my $gff, '<', "$top/COREFILES/GENOME/GeneNameList.gff3";
      while (<$gff>) {

            chomp $_;
            my @columns = split("\t", $_);

            if ($columns[1] eq 'gene') {

                  my $name = substr($columns[4], 0, -5);
                  if ((!(exists($start{$name}))) || ($columns[2] <
$start{$name})) { $start{$name} = $columns[2]; }
```

```
                if ((!(exists($stop{$name}))) || ($columns[3] >
$stop{$name}))  { $stop{$name} = $columns[3]; }

           } else {

                if ((!(exists($start{$columns[4]}))) || ($columns[2] <
$start{$columns[4]})) { $start{$columns[4]} = $columns[2]; }
                if ((!(exists($stop{$columns[4]}))) || ($columns[3] >
$stop{$columns[4]}))  { $stop{$columns[4]} = $columns[3]; }

           }

      }

}

my %midpoints;
open my $mid, '<', "$top/COREFILES/GENOME/GenePositions.txt";
while (<$mid>) {

      chomp $_;
      my @columns = split("\t", $_);
      $midpoints{$columns[2]} = ceil($columns[1]) ;

}

close $mid;

my %mappings;
open my $map, '<', "$top/COREFILES/GENOME/Nearest.txt";
while (<$map>) {

      chomp $_;
      my @columns = split("\t", $_);
      if ($number_of_close_genes > 10) { $number_of_close_genes = 10; }
      $mappings{$columns[0]} =
join("\t",@columns[1..$number_of_close_genes]);

}

close $map;

my %known;
open my $knownfile, '<', "$top/COREFILES/Genome/KnownGenes.txt";
my $header1 = <$knownfile>;
while (<$knownfile>) {

      chomp $_;
```

```
      my @columns = split("\t", $_);
      $known{$columns[0]} = join('/',@columns[1..2]);

}

close $knownfile;

my %rice;
open my $ricefile, '<', "$top/COREFILES/Genome/Rice.txt";
my $header = <$ricefile>;
while (<$ricefile>) {

      chomp $_;
      my @columns = split("\t", $_);
      $rice{$columns[0]} = join("\t",@columns[1..$#columns]);

}

close $ricefile;

my %arabidopsis;
open my $arabidopsisfile, '<',
"$top/COREFILES/Genome/Arabidopsis.txt";
my $header2 = <$arabidopsisfile>;
while (<$arabidopsisfile>) {

      chomp $_;
      my @columns = split("\t", $_);
      $arabidopsis{$columns[0]} = join("\t",@columns[1..$#columns]);

}

close $arabidopsisfile;

open my $RPath, '<', "$top/COREFILES/R_Installation.txt";
my $R = <$RPath>;
close $RPath;

print "Please place copies of your phenotype data in .txt format in
the base Pipeline folder.\nThe Pipeline will run them
sequentially.\nData files will be renamed and moved to the run folder
in /RUNS/.\n";
print "Please ensure the phenotype data is in the format in
/RUNS/Template.txt.\nPress any key to continue.\n>";
my $GO = <>;

my @datafiles = glob("$top/*.txt");
```

```
for my $file (@datafiles) { #Executes the Pipeline for every set of
trait data in the folder

opendir my $dh, "$top/RUNS"; #Gets the name of every existing
directory in RUNS
my @existing_runs = grep {-d "$top/RUNS/$_" && ! /^\.{1,2}$/}
readdir($dh);
closedir $dh;

open my $traitfile, '<', "$file"; #Gets the first line of the input
file
my $firstline = <$traitfile>;
close $traitfile;

$firstline =~ /\t(.*)$/; #Gets the trait name from the first line
my $trait = $1;
chomp $trait;
my $traitund = "$trait"."__";

my $numfix = 1; #Finds how many runs for this trait already exist in
RUNS and add 1 to the suffix
for my $run (@existing_runs) {

     my $suffx = (split('__',$run))[1];
     if ($run =~ m/$traitund/) {

          $numfix = $suffx+1;

     }

}

my $RunName = join('__', $trait,$numfix);

mkdir "$top/RUNS/$RunName", 0755; #Creates and populates the run
directory
mkdir "$top/RUNS/$RunName/TMP", 0755;
my $RunDataName = join('_', $trait,'DATA');

open my $translate, '<', "$file";
open my $trans_out, '>', "$top/RUNS/$RunName/$RunDataName.txt";
while (<$translate>) {

     #$_ =~ s///; #In case some global translation of input taxa names
is needed in future
     print $trans_out $_;

}
```

```
close $trans_out;

open my $names, '<', "$top/RUNS/$RunName/$RunDataName.txt"; #Slurp in
lines from data file
my @names = <$names>;
close $names;

my @exists; #Create an array of taxa with data
for (@names) {

      if ($_ !~ m/NaN/) { push @exists, (split("\t", $_))[0]; }

}

chomp @exists;

open my $reference, '<', "$top/COREFILES/GENOME/taxa.txt"; #Nab the
taxa list
my @columns = split("\t", <$reference>); #Get an array of taxa names
close $reference;
chomp @columns;

my @keep; #Array of taxa names to keep

for my $j (0..$#columns) { #Pushes only taxa for which there is data
to @keep by looping through @exists for each value in @columns

      my $flg = 0;
      for my $k (0..$#exists) {

            if ($exists[$k] eq $columns[$j]) { $flg = 1; } #If the
taxon is present, set the flag to 1 so it gets pushed to @keep

      }
      if ($flg == 1) { push @keep, $columns[$j]; }

}

my $taxa = scalar(@keep); #Number of taxa is equal to the number of
kept columns
my %to_keep = map { $_ => 1 } @keep;

print "Scaling Marker Sets ($taxa Individuals)...\n";
for my $i (1..10) {

      die "could not fork" unless defined(my $prepid = fork); #Parallel
processing of genotype files
```

```
     unless ($prepid) {

          copy("$top/COREFILES/GENOME/$i.GM.txt",
"$top/RUNS/$RunName/TMP/$i.GM.txt");
          open my $in, '<', "$top/COREFILES/GENOME/$i.GD.txt";
          open my $out, '>', "$top/RUNS/$RunName/TMP/$i.GD.txt";
          my $header = <$in>;
          print $out $header;

          while (<$in>) {

               my $taxon = (split("\t", $_))[0];
               if(exists($to_keep{$taxon})) { print $out $_; }

          }

          close $in;
          close $out;
          exit;

     }

}

while (1) {

  my $child = waitpid(-1, 0);
  last if $child == -1;

}

if ($do_serial == 1) {

print "Executing Chromosomal MLM via GAPIT for $trait serially...\n";
for my $i (1..10) { #Simultaneously executes GAPIT for all 10
chromosomes

     my $k = "$i"."k";
     my $I = $i;
     mkdir "$top/RUNS/$RunName/TMP/$i", 0755;
     open my $rfile, '>', "$top/RUNS/$RunName/TMP/$i/R.txt";
     my $core = dirname(abs_path($0));

     print $rfile qq`library(compiler)
source("$core/COREFILES/GAPIT/gapit_functions.txt")
source("$core/COREFILES/GAPIT/emma.txt")
source("$core/COREFILES/GAPIT/mt.txt")
library(gplots)
```

```
library(LDheatmap)
library(genetics)
library(EMMREML)
library(scatterplot3d)
myGD <- read.table("$core/RUNS/$RunName/TMP/$i.GD.txt", head=TRUE)
myGM <- read.table("$core/RUNS/$RunName/TMP/$i.GM.txt", head=TRUE)
myKI <- read.table("$core/COREFILES/GENOME/$k.txt", head=FALSE)
myY <- read.table("$core/RUNS/$RunName/$RunDataName.txt", head=TRUE)
setwd("$top/RUNS/$RunName/TMP/$i")
myGAPIT <- GAPIT(Y=myY, GD=myGD,GM=myGM, KI=myKI, group.from=282,
group.to=282, Geno.View.output=FALSE)
q()
`;
     close $rfile;
     my $command = "$R/Rscript $top/RUNS/$RunName/TMP/$i/R.txt > nul
2>\&1";
     system("$command");
     print "Chromosome $i complete!\n";

}

} else {

print "Executing Chromosomal MLM via GAPIT for $trait in
parallel...\n";
for my $i (1..10) { #Simultaneously executes GAPIT for all 10
chromosomes

     die "could not fork" unless defined(my $pid = fork);
     unless ($pid) {

          my $k = "$i"."k";
          my $I = $i;
          mkdir "$top/RUNS/$RunName/TMP/$i", 0755;
          open my $rfile, '>', "$top/RUNS/$RunName/TMP/$i/R.txt";
          my $core = dirname(abs_path($0));

          print $rfile qq`library(compiler)
source("$core/COREFILES/GAPIT/gapit_functions.txt")
source("$core/COREFILES/GAPIT/emma.txt")
source("$core/COREFILES/GAPIT/mt.txt")
library(gplots)
library(LDheatmap)
library(genetics)
library(EMMREML)
library(scatterplot3d)
myGD <- read.table("$core/RUNS/$RunName/TMP/$i.GD.txt", head=TRUE)
myGM <- read.table("$core/RUNS/$RunName/TMP/$i.GM.txt", head=TRUE)
```

```
myKI <- read.table("$core/COREFILES/GENOME/$k.txt", head=FALSE)
myY <- read.table("$core/RUNS/$RunName/$RunDataName.txt", head=TRUE)
setwd("$core/RUNS/$RunName/TMP/$i")
myGAPIT <- GAPIT(Y=myY, GD=myGD,GM=myGM, KI=myKI, group.from=282,
group.to=282, Geno.View.output=FALSE)
q()
`;
           close $rfile;
           my $command = "$R/Rscript $top/RUNS/$RunName/TMP/$i/R.txt >
nul 2>\&1";
           system("$command");
           print "Chromosome $i complete!\n";
           exit;

      }

}

while (1) {

  my $child = waitpid(-1, 0);
  last if $child == -1;

}

}

print "MLM Complete! Compiling results...\n"; #Compiles GWAS Results

open my $rfile, '>', "$top/RUNS/$RunName/TMP/R1.txt";
my $core = dirname(abs_path($0));

print $rfile qq`library(compiler)
source("$core/COREFILES/GAPIT/gapit_functions.txt")
source("$core/COREFILES/GAPIT/mt.txt")
library(gplots)
library(genetics)
library(EMMREML)
library("scatterplot3d")
library(MASS)
source("$core/COREFILES/GAPIT/manhattan.txt")
library(manhattanly)
library(plotly)
mydataPath.Results.1="$core/RUNS/$RunName/TMP/1/"
mydataPath.Results.2="$core/RUNS/$RunName/TMP/2/"
mydataPath.Results.3="$core/RUNS/$RunName/TMP/3/"
mydataPath.Results.4="$core/RUNS/$RunName/TMP/4/"
mydataPath.Results.5="$core/RUNS/$RunName/TMP/5/"
```

```
mydataPath.Results.6="$core/RUNS/$RunName/TMP/6/"
mydataPath.Results.7="$core/RUNS/$RunName/TMP/7/"
mydataPath.Results.8="$core/RUNS/$RunName/TMP/8/"
mydataPath.Results.9="$core/RUNS/$RunName/TMP/9/"
mydataPath.Results.10="$core/RUNS/$RunName/TMP/10/"
name <- "$trait"
GWAS.Results.1 <-
read.csv(paste(mydataPath.Results.1,"GAPIT..",name,".GWAS.Results.csv"
,sep=""), head=TRUE)
GWAS.Results.2 <-
read.csv(paste(mydataPath.Results.2,"GAPIT..",name,".GWAS.Results.csv"
,sep=""), head=TRUE)
GWAS.Results.3 <-
read.csv(paste(mydataPath.Results.3,"GAPIT..",name,".GWAS.Results.csv"
,sep=""), head=TRUE)
GWAS.Results.4 <-
read.csv(paste(mydataPath.Results.4,"GAPIT..",name,".GWAS.Results.csv"
,sep=""), head=TRUE)
GWAS.Results.5 <-
read.csv(paste(mydataPath.Results.5,"GAPIT..",name,".GWAS.Results.csv"
,sep=""), head=TRUE)
GWAS.Results.6 <-
read.csv(paste(mydataPath.Results.6,"GAPIT..",name,".GWAS.Results.csv"
,sep=""), head=TRUE)
GWAS.Results.7 <-
read.csv(paste(mydataPath.Results.7,"GAPIT..",name,".GWAS.Results.csv"
,sep=""), head=TRUE)
GWAS.Results.8 <-
read.csv(paste(mydataPath.Results.8,"GAPIT..",name,".GWAS.Results.csv"
,sep=""), head=TRUE)
GWAS.Results.9 <-
read.csv(paste(mydataPath.Results.9,"GAPIT..",name,".GWAS.Results.csv"
,sep=""), head=TRUE)
GWAS.Results.10 <-
read.csv(paste(mydataPath.Results.10,"GAPIT..",name,".GWAS.Results.csv
",sep=""), head=TRUE)
Effect.Estimates.1 <-
read.csv(paste(mydataPath.Results.1,"GAPIT..",name,".Allelic_Effect_Es
timates.csv",sep=""), head=TRUE)
Effect.Estimates.2 <-
read.csv(paste(mydataPath.Results.2,"GAPIT..",name,".Allelic_Effect_Es
timates.csv",sep=""), head=TRUE)
Effect.Estimates.3 <-
read.csv(paste(mydataPath.Results.3,"GAPIT..",name,".Allelic_Effect_Es
timates.csv",sep=""), head=TRUE)
Effect.Estimates.4 <-
read.csv(paste(mydataPath.Results.4,"GAPIT..",name,".Allelic_Effect_Es
timates.csv",sep=""), head=TRUE)
```

```
Effect.Estimates.5 <-
read.csv(paste(mydataPath.Results.5,"GAPIT..",name,".Allelic_Effect_Es
timates.csv",sep=""), head=TRUE)
Effect.Estimates.6 <-
read.csv(paste(mydataPath.Results.6,"GAPIT..",name,".Allelic_Effect_Es
timates.csv",sep=""), head=TRUE)
Effect.Estimates.7 <-
read.csv(paste(mydataPath.Results.7,"GAPIT..",name,".Allelic_Effect_Es
timates.csv",sep=""), head=TRUE)
Effect.Estimates.8 <-
read.csv(paste(mydataPath.Results.8,"GAPIT..",name,".Allelic_Effect_Es
timates.csv",sep=""), head=TRUE)
Effect.Estimates.9 <-
read.csv(paste(mydataPath.Results.9,"GAPIT..",name,".Allelic_Effect_Es
timates.csv",sep=""), head=TRUE)
Effect.Estimates.10 <-
read.csv(paste(mydataPath.Results.10,"GAPIT..",name,".Allelic_Effect_E
stimates.csv",sep=""), head=TRUE)
GWAS.Results <- rbind(GWAS.Results.1, GWAS.Results.2, GWAS.Results.3,
GWAS.Results.4, GWAS.Results.5, GWAS.Results.6, GWAS.Results.7,
GWAS.Results.8, GWAS.Results.9, GWAS.Results.10)
Effect.Estimates <- rbind(Effect.Estimates.1, Effect.Estimates.2,
Effect.Estimates.3, Effect.Estimates.4, Effect.Estimates.5,
Effect.Estimates.6, Effect.Estimates.7, Effect.Estimates.8,
Effect.Estimates.9, Effect.Estimates.10)
GWAS.Results <- GWAS.Results[,-ncol(GWAS.Results)]
setwd("$core/RUNS/$RunName")
Conduct.FDR <-
GAPIT.Perform.BH.FDR.Multiple.Correction.Procedure(PWI=GWAS.Results,
FDR.Rate=$bjh_alpha, FDR.Procedure="BH")
GWAS.Results.FDR <- Conduct.FDR\$PWIP
write.table(GWAS.Results.FDR, paste("GAPIT.", name,
".GWAS.Results.txt", sep = ""), quote = FALSE, sep = "\t", row.names =
FALSE, col.names = TRUE)
write.table(Effect.Estimates, paste("GAPIT.", name,
".Allelic_Effect_Estimates.txt", sep = ""), quote = FALSE, sep = "\t",
row.names = FALSE, col.names = TRUE)
`;

close $rfile;
my $command = "$R/Rscript $top/RUNS/$RunName/TMP/R1.txt > nul 2>\&1";
system("$command");

move("$top/RUNS/$RunName/GAPIT.$trait.Allelic_Effect_Estimates.txt",
"$top/RUNS/$RunName/$trait"."_Effects.txt");
move("$top/RUNS/$RunName/GAPIT.$trait.GWAS.Results.txt",
"$top/RUNS/$RunName/$trait"."_StatisticsTMP.txt");
```

```
open my $in, '<', "$top/RUNS/$RunName/$trait"."_StatisticsTMP.txt";
#Creates a new statistics file sorted by corrected FDR P-value
my $discard = <$in>;
my @lines = <$in>;
chomp @lines;
close $in;

my @ordered_lines = sort { (split("\t", $a))[1] <=> (split("\t",
$b))[1] || (split("\t", $a))[2] <=> (split("\t", $b))[2] } @lines;

open my $out, '>', "$top/RUNS/$RunName/$trait"."_Statistics.txt";
for (@ordered_lines) { print $out "$_\n"; }
close $out;

my @BJH; #Pushes to @BJH FDR P-values below $bjh_alpha, these hits are
colored in the final Manhattan plot
open $in,  '<',  "$top/RUNS/$RunName/$trait"."_Statistics.txt";

while (<$in>) {

     #if ((split("\t", $_))[8] < $bjh_alpha) { #bjh-corrected p-value
below BJH threshold
     if ((split("\t", $_))[3] < $pcalc[(split("\t", $_))[1]-1]) {
#uncorrected p-value below Bonferroni threshold

          push @BJH, (split("\t", $_))[0];

     }

}

close $in;
my $R_HITS = join('","', @BJH);

#Assimilate the final interactive Manhattan plots
open $rfile, '>', "$top/RUNS/$RunName/TMP/R2.txt";
print $rfile qq`capture.output()
setwd("$core/RUNS/$RunName")
name <- "$trait"
library(compiler)
source("$core/COREFILES/GAPIT/gapit_functions.txt")
source("$core/COREFILES/GAPIT/mt.txt")
library(gplots)
library(genetics)
library(EMMREML)
library("scatterplot3d")
library(MASS)
source("$core/COREFILES/GAPIT/manhattan.txt")
```

```
library(manhattanly)
library(plotly)
GWAS.Results <- read.table(paste("$core/RUNS/$RunName/$trait",
"_StatisticsTMP.txt", sep = ""), head=TRUE)
SNPsOfInterest <- c("$R_HITS")
GAPIT.QQ(P.values = GWAS.Results[,4], name.of.trait = name,DPP=50000)
manhattan(GWAS.Results, genomewideline=$avg_Y, chr="Chromosome",
bp="Position", p="P.value", snp="SNP", col=c("black", "gold3"), main =
"Manhattan Plot for $trait", highlight = SNPsOfInterest)
manhattan(subset(GWAS.Results, Chromosome==1), chr="Chromosome",
bp="Position", p="P.value", snp="SNP", col=c("black"),
genomewideline=$b1, main = "Manhattan Plot for $trait", highlight =
SNPsOfInterest)
manhattan(subset(GWAS.Results, Chromosome==2), chr="Chromosome",
bp="Position", p="P.value", snp="SNP", col=c("black"),
genomewideline=$b2, main = "Manhattan Plot for $trait", highlight =
SNPsOfInterest)
manhattan(subset(GWAS.Results, Chromosome==3), chr="Chromosome",
bp="Position", p="P.value", snp="SNP", col=c("black"),
genomewideline=$b3, main = "Manhattan Plot for $trait", highlight =
SNPsOfInterest)
manhattan(subset(GWAS.Results, Chromosome==4), chr="Chromosome",
bp="Position", p="P.value", snp="SNP", col=c("black"),
genomewideline=$b4, main = "Manhattan Plot for $trait", highlight =
SNPsOfInterest)
manhattan(subset(GWAS.Results, Chromosome==5), chr="Chromosome",
bp="Position", p="P.value", snp="SNP", col=c("black"),
genomewideline=$b5, main = "Manhattan Plot for $trait", highlight =
SNPsOfInterest)
manhattan(subset(GWAS.Results, Chromosome==6), chr="Chromosome",
bp="Position", p="P.value", snp="SNP", col=c("black"),
genomewideline=$b6, main = "Manhattan Plot for $trait", highlight =
SNPsOfInterest)
manhattan(subset(GWAS.Results, Chromosome==7), chr="Chromosome",
bp="Position", p="P.value", snp="SNP", col=c("black"),
genomewideline=$b7, main = "Manhattan Plot for $trait", highlight =
SNPsOfInterest)
manhattan(subset(GWAS.Results, Chromosome==8), chr="Chromosome",
bp="Position", p="P.value", snp="SNP", col=c("black"),
genomewideline=$b8, main = "Manhattan Plot for $trait", highlight =
SNPsOfInterest)
manhattan(subset(GWAS.Results, Chromosome==9), chr="Chromosome",
bp="Position", p="P.value", snp="SNP", col=c("black"),
genomewideline=$b9, main = "Manhattan Plot for $trait", highlight =
SNPsOfInterest)
manhattan(subset(GWAS.Results, Chromosome==10), chr="Chromosome",
bp="Position", p="P.value", snp="SNP", col=c("black"),
```

```
genomewideline=$b10, main = "Manhattan Plot for $trait", highlight =
SNPsOfInterest)
GR <- manhattanr(GWAS.Results, chr = "Chromosome", bp = "Position", p
= "P.value")
htmlwidgets::saveWidget(manhattanly(subset(GR[["data"]], CHR %in% 1),
genomewideline=$b1, title = "Manhattan Plot for $trait",
col=c("black")), "Chr_1.html")
htmlwidgets::saveWidget(manhattanly(subset(GR[["data"]], CHR %in% 2),
genomewideline=$b2, title = "Manhattan Plot for $trait",
col=c("black")), "Chr_2.html")
htmlwidgets::saveWidget(manhattanly(subset(GR[["data"]], CHR %in% 3),
genomewideline=$b3, title = "Manhattan Plot for $trait",
col=c("black")), "Chr_3.html")
htmlwidgets::saveWidget(manhattanly(subset(GR[["data"]], CHR %in% 4),
genomewideline=$b4, title = "Manhattan Plot for $trait",
col=c("black")), "Chr_4.html")
htmlwidgets::saveWidget(manhattanly(subset(GR[["data"]], CHR %in% 5),
genomewideline=$b5, title = "Manhattan Plot for $trait",
col=c("black")), "Chr_5.html")
htmlwidgets::saveWidget(manhattanly(subset(GR[["data"]], CHR %in% 6),
genomewideline=$b6, title = "Manhattan Plot for $trait",
col=c("black")), "Chr_6.html")
htmlwidgets::saveWidget(manhattanly(subset(GR[["data"]], CHR %in% 7),
genomewideline=$b7, title = "Manhattan Plot for $trait",
col=c("black")), "Chr_7.html")
htmlwidgets::saveWidget(manhattanly(subset(GR[["data"]], CHR %in% 8),
genomewideline=$b8, title = "Manhattan Plot for $trait",
col=c("black")), "Chr_8.html")
htmlwidgets::saveWidget(manhattanly(subset(GR[["data"]], CHR %in% 9),
genomewideline=$b9, title = "Manhattan Plot for $trait",
col=c("black")), "Chr_9.html")
htmlwidgets::saveWidget(manhattanly(subset(GR[["data"]], CHR %in% 10),
genomewideline=$b10, title = "Manhattan Plot for $trait",
col=c("black")), "Chr_10.html")
rm(GWAS.Results)
rm(Effect.Estimates)
`;

close $rfile;
$command = "$R/Rscript $top/RUNS/$RunName/TMP/R2.txt > nul 2>\&1";
system("$command");

print "Results Compiled! Cleaning...\n"; #Finish sorting out GAPIT
outputs
move("$top/RUNS/$RunName/Rplots.pdf",
"$top/RUNS/$RunName/$trait"."_Manhattan.pdf");
move("$top/RUNS/$RunName/GAPIT.$trait.QQ-Plot.pdf",
"$top/RUNS/$RunName/$trait"."_QQ.pdf");
```

```
unless ($debug == 1) { rmtree("$top/RUNS/$RunName/TMP"); }
print "Results Cleaned! Beginning Interpretation...\n";

my @Hi_Mom;

open $out, '>', "$top/RUNS/$RunName/$trait"."_SignificantHits";
open $in, '<', "$top/RUNS/$RunName/$trait"."_Statistics.txt";
my $head = (<$in>);

print "Selecting Significant Results at FDR p-value < $bjh_alpha or
average Bonferroni p-value < $avg_p (alpha=$bonf_alpha)!\n";
print "Also printing results where p < $p_annotate for
completeness.\n";

while (<$in>) { #Prints Significant Hits to output file

	chomp $_;
	my @names = split("\t", $_);
	my $SNP=$names[0];
	my $Chrom=$names[1];
	my $BP=$names[2];
	my $pval=$names[3];
	my $fdrpval=$names[8];

	#if (($fdrpval < $bjh_alpha) || ($pval < $pcalc[$Chrom-1]) ||
($pval < $p_annotate)) { #must be below BJH, bonferroni, AND user-
specified threshold
	#if ($fdrpval < $bjh_alpha) { #must be below only BJH threshold
	if ($pval < $pcalc[$Chrom-1]) { #must be below only user
threshold

		my $tmpos = join(",",$SNP,$Chrom,$BP,$pval,$fdrpval);
		chomp $tmpos;
		print $out "$tmpos\n";

	}

}

close $out;
close $in;

open $in, '<', "$top/RUNS/$RunName/$trait"."_SignificantHits";
open my $finalarabidopsis, '>',
"$top/RUNS/$RunName/$trait"."_Arabidopsis.txt";
open my $finalrice, '>', "$top/RUNS/$RunName/$trait"."_Rice.txt";
```

```
print $finalarabidopsis "SNP\tChromosome\tLocus\tMLM p-value\tBJH p-value\tGene Midpoint\tGene Distance\tType\tMaize Gene\tArabidopsis Gene\tDescription\tBLAST Alignent Score\tE-value\n";
print $finalrice "SNP\tChromosome\tLocus\tMLM p-value\tBJH p-value\tGene Midpoint\tGene Distance\tType\tMaize Gene\tRice Gene\tDescription\tBLAST Alignent Score\tE-value\n";

while (<$in>) {

      chomp $_;
      my @gwaspos = split(',',$_);
      my $SNP=$gwaspos[0];
      my $chromosome=$gwaspos[1];
      my $posit=$gwaspos[2];
      my $pval=$gwaspos[3];
      my $fdrpval=$gwaspos[4];

      my @genelist = split("\t", $mappings{$SNP});
      for my $gene (@genelist) {

            if ($use_ld == 1) {

                  my $startdistance = abs($start{$gene} - $posit);
                  my $stopdistance = abs($stop{$gene} - $posit);
                  unless (($startdistance <= $ld_window) ||
($stopdistance <= $ld_window)) { next; }

            }

            my $midpoint = $midpoints{$gene};
            my $distance = $midpoint-$posit;
            my $type = $nonprot{$gene} // "gene";
            my $name = $known{$gene} // "none";
            my $rice = "none\tnone\tnone\tnone";
            my $arabidopsis = "none\tnone\tnone\tnone";

            if (exists($arabidopsis{$gene})) {

                  $arabidopsis = $arabidopsis{$gene};

            }

            if (exists($rice{$gene})) {

                  $rice = $rice{$gene};

            }
```

```
          print $finalarabidopsis
"$SNP\t$chromosome\t$posit\t$pval\t$fdrpval\t$midpoint\t$distance\t$ty
pe\t$gene\t$name\t$arabidopsis\n";
          print $finalrice
"$SNP\t$chromosome\t$posit\t$pval\t$fdrpval\t$midpoint\t$distance\t$ty
pe\t$gene\t$name\t$rice\n";

     }

     print $finalarabidopsis "\n";
     print $finalrice "\n";

}

close $in;
close $finalarabidopsis;
close $finalrice;

print "Final Report Assembled!\n";

unless ($debug == 1) {

     unlink "$top/RUNS/$RunName/$trait"."_StatisticsTMP.txt";

}

mkdir "$top/RUNS/$RunName/INTERMEDIATES";
move("$top/RUNS/$RunName/$trait"."_Statistics.txt",
"$top/RUNS/$RunName/INTERMEDIATES/$trait"."_Statistics.txt");
move("$top/RUNS/$RunName/$trait"."_Effects.txt",
"$top/RUNS/$RunName/INTERMEDIATES/$trait"."_Effects.txt");
move("$top/RUNS/$RunName/$trait"."_SignificantHits",
"$top/RUNS/$RunName/INTERMEDIATES/$trait"."_SignificantHits.txt");

open $out, '>',
"$top/RUNS/$RunName/INTERMEDIATES/$trait"."_Significance_Thresholds.tx
t";
print $out "Bonferroni Alpha: $bonf_alpha\nFDR-corrected p-value
Threshold: $bjh_alpha\nAverage Bonferroni Threshold p-value:
$avg_p\nAverage Bonferroni Threshold Y-value: $avg_Y\n";
close $out;

print "Cleanup complete!\n";

}

print "GWAS PIPELINE COMPLETE!\n";
print "Press ENTER to Exit.\n>";
```

```
my $DONE = <>;
```

Dataset 2, Script 2

```
#configuration options start at line 38 for:
#    threshold options for selecting candidate significant markers. which of these options to use is defined at lines 484-485 and 571-573
#    method for assigning markers to genes-- specified LD window or n closest genes to marker

#more in-depth customization:
#    GAPIT model defined at lines 343, 381, together with surrounding script. For example, enable compression by setting the group.from parameter to 0, and/or by adding a group.to parameter to adjust the groupings.

#STRUCTURE
#SD2/NCRPIS_2.7/
#├── SD2-S2.pl #script to execute COMPILE
#├── input *.txt file(s) containing phenotype data
#├── RUNS/
#|     ├── Example.txt #contains example input data in correct format
#|     └── RESULTS_FOLDERS #one per input data file
#|           ├── INTERMEDIATES/ #raw GAPIT output files, including some not used by COMPILE (e.g. reports of marker effect size)
#|           ├── output *_DATA.txt #copy of original input phenotype data
#|           ├── output *_Manhattan.pdf #Manhattan plots of results
#|           ├── output *_QQ.pdf #quantile-quantile plot of results
#|           ├── output *_Arabidopsis.txt #candidate gene report matching maize to arabidopsis genes
#|           └── output *_Rice.txt #candidate gene report matching maize to rice genes
#└── COREFILES/
#     ├── GAPIT/ #contains files used by GAPIT to execute GWAS
#     └── GENOME/
#           ├── 1.GD.txt - 10.GD.txt, 1.GM.txt - 10.GM.txt #marker data in GAPIT numerical format (SD1-S2, SD1-S3)
#           ├── 1k.txt - 10k.txt #kinship matrix data (SD1-S4)
#           ├── GeneNameList.gff3 #filtered gene info list (SD1-S5)
#           ├── GenePositions.txt #list of gene positions (SD1-S6)
#           ├── KnownGenes.txt #list of known genes in maize
#           ├── taxa.txt #list of taxa
#           ├── Nearest.txt #atlas of nearest ten genes to each marker (SD1-S7)
#           └── Rice.txt, Arabidopsis.txt #sequence similarity databases relating maize to rice and arabidopsis (SD1-S8, SD1-S9)

use strict;
use warnings;
use Cwd qw(abs_path);
```

```
use File::Copy;
use File::Path;
use File::Basename;
use List::Util qw(sum);
use POSIX qw(ceil);

my $debug = 0; #If 1, doesn't delete temporary files or directories
my $do_serial = 0; #If 1, runs chromosomes in order 1-10 instead of in
parallel
my $bjh_alpha = 0.05; #Significance threshold for BJH FDR p-val
my $bonf_alpha = 0.05; #Significance threshold for Bonferroni alpha
my $p_annotate = 0.00001; #Significance threshold to annotate markers
anyway

my $use_ld = 0; #whether to use number of close genes or LD window.
$number_of_close_genes option will still function, e.g. if >1 gene
within the LD window, so set appropriately.
my $number_of_close_genes = 10; #number of genes near each marker to
annotate (max 10)
my $ld_window = 10000; #window in bp around each marker to find genes
to annotate

my $type = fileparse_set_fstype("Unix"); #Allows spaces in file paths
my $dirname = dirname(__FILE__);
my $top = defined($dirname) ? $dirname : '.';

my @num_markers;
for my $i (1..10) { #Get numbers of markers on each chromosome

     open my $file, '<', "$top/COREFILES/GENOME/$i.GM.txt";
     while (<$file>) {}
     my $line = $.-1; #0-index takes care of -1 for empty line at end;
another -1 for header
     push @num_markers, $line;
     close $file;

}

my @bonferroni;
my @pcalc;
for my $nm (@num_markers) {

     push @pcalc, $bonf_alpha/$nm; #Y-values for significance
thresholds on plot
     my $x = -(log($bonf_alpha/$nm)/log(10));
     push @bonferroni, $x;

}
```

```
my $avg_p = sum(@pcalc)/@pcalc;
my $avg_Y = -(log($avg_p)/log(10)); #Average Bonferroni significance
threshold across all 10 chromosomes

my $b1 = $bonferroni[0]; #Because R doesn't accept the array value; I
know it looks bad
my $b2 = $bonferroni[1];
my $b3 = $bonferroni[2];
my $b4 = $bonferroni[3];
my $b5 = $bonferroni[4];
my $b6 = $bonferroni[5];
my $b7 = $bonferroni[6];
my $b8 = $bonferroni[7];
my $b9 = $bonferroni[8];
my $b10 = $bonferroni[9];

print "Initializing, please wait a moment...\n";

my %nonprot;
open my $gff, '<', "$top/COREFILES/GENOME/GeneNameList.gff3";
while (<$gff>) {

      chomp $_;
      my @columns = split("\t", $_);
      if ($columns[1] ne 'gene') { $nonprot{$columns[4]} = $columns[1];
}

}

close $gff;

my %start;
my %stop;
if ($use_ld == 1) {

      open my $gff, '<', "$top/COREFILES/GENOME/GeneNameList.gff3";
      while (<$gff>) {

            chomp $_;
            my @columns = split("\t", $_);

            if ($columns[1] eq 'gene') {

                  my $name = substr($columns[4], 0, -5);
                  if ((!(exists($start{$name}))) || ($columns[2] <
$start{$name})) { $start{$name} = $columns[2]; }
```

```
                if ((!(exists($stop{$name}))) || ($columns[3] >
$stop{$name}))  { $stop{$name} = $columns[3]; }

           } else {

                if ((!(exists($start{$columns[4]}))) || ($columns[2] <
$start{$columns[4]})) { $start{$columns[4]} = $columns[2]; }
                if ((!(exists($stop{$columns[4]}))) || ($columns[3] >
$stop{$columns[4]}))  { $stop{$columns[4]} = $columns[3]; }

           }

      }

}

my %midpoints;
open my $mid, '<', "$top/COREFILES/GENOME/GenePositions.txt";
while (<$mid>) {

      chomp $_;
      my @columns = split("\t", $_);
      $midpoints{$columns[2]} = ceil($columns[1]) ;

}

close $mid;

my %mappings;
open my $map, '<', "$top/COREFILES/GENOME/Nearest.txt";
while (<$map>) {

      chomp $_;
      my @columns = split("\t", $_);
      if ($number_of_close_genes > 10) { $number_of_close_genes = 10; }
      $mappings{$columns[0]} =
join("\t",@columns[1..$number_of_close_genes]);

}

close $map;

my %known;
open my $knownfile, '<', "$top/COREFILES/Genome/KnownGenes.txt";
my $header = <$knownfile>;
while (<$knownfile>) {

      chomp $_;
```

```
      my @columns = split("\t", $_);
      $known{$columns[0]} = join('/',@columns[1..2]);

}

close $knownfile;

my %rice;
open my $ricefile, '<', "$top/COREFILES/Genome/Rice.txt";
my $header1 = <$ricefile>;
while (<$ricefile>) {

      chomp $_;
      my @columns = split("\t", $_);
      $rice{$columns[0]} = join("\t",@columns[1..$#columns]);

}

close $ricefile;

my %arabidopsis;
open my $arabidopsisfile, '<',
"$top/COREFILES/Genome/Arabidopsis.txt";
my $header2 = <$arabidopsisfile>;
while (<$arabidopsisfile>) {

      chomp $_;
      my @columns = split("\t", $_);
      $arabidopsis{$columns[0]} = join("\t",@columns[1..$#columns]);

}

close $arabidopsisfile;

open my $RPath, '<', "$top/COREFILES/R_Installation.txt";
my $R = <$RPath>;
close $RPath;

print "Please place copies of your phenotype data in .txt format in
the base Pipeline folder.\nThe Pipeline will run them
sequentially.\nData files will be renamed and moved to the run folder
in /RUNS/.\n";
print "Please ensure the phenotype data is in the format in
/RUNS/Template.txt.\nPress any key to continue.\n>";
my $GO = <>;

my @datafiles = glob("$top/*.txt");
```

```
for my $file (@datafiles) { #Executes the Pipeline for every set of
trait data in the folder

opendir my $dh, "$top/RUNS"; #Gets the name of every existing
directory in RUNS
my @existing_runs = grep {-d "$top/RUNS/$_" && ! /^\.{1,2}$/}
readdir($dh);
closedir $dh;

open my $traitfile, '<', "$file"; #Gets the first line of the input
file
my $firstline = <$traitfile>;
close $traitfile;

$firstline =~ /\t(.*)$/; #Gets the trait name from the first line
my $trait = $1;
chomp $trait;
my $traitund = "$trait"."__";

my $numfix = 1; #Finds how many runs for this trait already exist in
RUNS and add 1 to the suffix
for my $run (@existing_runs) {

      my $suffx = (split('__',$run))[1];
      if ($run =~ m/$traitund/) {

            $numfix = $suffx+1;

      }

}

my $RunName = join('__', $trait,$numfix);

mkdir "$top/RUNS/$RunName", 0755; #Creates and populates the run
directory
mkdir "$top/RUNS/$RunName/TMP", 0755;
my $RunDataName = join('_', $trait,'DATA');

open my $translate, '<', "$file";
open my $trans_out, '>', "$top/RUNS/$RunName/$RunDataName.txt";
while (<$translate>) {

      #$_ =~ s///; #In case some global translation of input taxa names
is needed in future
      print $trans_out $_;

}
```

```
close $trans_out;

open my $names, '<', "$top/RUNS/$RunName/$RunDataName.txt"; #Slurp in
lines from data file
my @names = <$names>;
close $names;

my @exists; #Create an array of taxa with data
for (@names) {

	if ($_ !~ m/NaN/) { push @exists, (split("\t", $_))[0]; }

}

chomp @exists;

open my $reference, '<', "$top/COREFILES/GENOME/taxa.txt"; #Nab the
taxa list
my @columns = split("\t", <$reference>); #Get an array of taxa names
close $reference;
chomp @columns;

my @keep; #Array of taxa names to keep

for my $j (0..$#columns) { #Pushes only taxa for which there is data
to @keep by looping through @exists for each value in @columns

	my $flg = 0;
	for my $k (0..$#exists) {

		if ($exists[$k] eq $columns[$j]) { $flg = 1; } #If the
taxon is present, set the flag to 1 so it gets pushed to @keep

	}
	if ($flg == 1) { push @keep, $columns[$j]; }

}

my $taxa = scalar(@keep); #Number of taxa is equal to the number of
kept columns
my %to_keep = map { $_ => 1 } @keep;

print "Scaling Marker Sets ($taxa Individuals)...\n";
for my $i (1..10) {

	die "could not fork" unless defined(my $prepid = fork); #Parallel
processing of genotype files
```

```
      unless ($prepid) {

            copy("$top/COREFILES/GENOME/$i.GM.txt",
"$top/RUNS/$RunName/TMP/$i.GM.txt");
            open my $in, '<', "$top/COREFILES/GENOME/$i.GD.txt";
            open my $out, '>', "$top/RUNS/$RunName/TMP/$i.GD.txt";
            my $header = <$in>;
            print $out $header;

            while (<$in>) {

                  my $taxon = (split("\t", $_))[0];
                  if(exists($to_keep{$taxon})) { print $out $_; }

            }

            close $in;
            close $out;
            exit;

      }

}

while (1) {

  my $child = waitpid(-1, 0);
  last if $child == -1;

}

if ($do_serial == 1) {

print "Executing Chromosomal MLM via GAPIT for $trait serially...\n";
for my $i (1..10) { #Simultaneously executes GAPIT for all 10
chromosomes

      my $k = "$i"."k";
      my $I = $i;
      mkdir "$top/RUNS/$RunName/TMP/$i", 0755;
      open my $rfile, '>', "$top/RUNS/$RunName/TMP/$i/R.txt";
      my $core = dirname(abs_path($0));

      print $rfile qq`library(compiler)
source("$core/COREFILES/GAPIT/gapit_functions.txt")
source("$core/COREFILES/GAPIT/emma.txt")
source("$core/COREFILES/GAPIT/mt.txt")
library(gplots)
```

```
library(LDheatmap)
library(genetics)
library(EMMREML)
library(scatterplot3d)
myGD <- read.table("$core/RUNS/$RunName/TMP/$i.GD.txt", head=TRUE)
myGM <- read.table("$core/RUNS/$RunName/TMP/$i.GM.txt", head=TRUE)
myKI <- read.table("$core/COREFILES/GENOME/$k.txt", head=FALSE)
myY <- read.table("$core/RUNS/$RunName/$RunDataName.txt", head=TRUE)
setwd("$core/RUNS/$RunName/TMP/$i")
myGAPIT <- GAPIT(Y=myY, GD=myGD,GM=myGM, KI=myKI, group.from=2853,
group.to=2853, Geno.View.output=FALSE)
q()
`;
      close $rfile;
      my $command = "$R/Rscript $top/RUNS/$RunName/TMP/$i/R.txt > nul
2>\&1";
      system("$command");
      print "Chromosome $i complete!\n";

}

} else {

print "Executing Chromosomal MLM via GAPIT for $trait in
parallel...\n";
for my $i (1..10) { #Simultaneously executes GAPIT for all 10
chromosomes

      die "could not fork" unless defined(my $pid = fork);
      unless ($pid) {

            my $k = "$i"."k";
            my $I = $i;
            mkdir "$top/RUNS/$RunName/TMP/$i", 0755;
            open my $rfile, '>', "$top/RUNS/$RunName/TMP/$i/R.txt";
            my $core = dirname(abs_path($0));

            print $rfile qq`library(compiler)
source("$core/COREFILES/GAPIT/gapit_functions.txt")
source("$core/COREFILES/GAPIT/emma.txt")
source("$core/COREFILES/GAPIT/mt.txt")
library(gplots)
library(LDheatmap)
library(genetics)
library(EMMREML)
library(scatterplot3d)
myGD <- read.table("$core/RUNS/$RunName/TMP/$i.GD.txt", head=TRUE)
myGM <- read.table("$core/RUNS/$RunName/TMP/$i.GM.txt", head=TRUE)
```

```
myKI <- read.table("$core/COREFILES/GENOME/$k.txt", head=FALSE)
myY <- read.table("$core/RUNS/$RunName/$RunDataName.txt", head=TRUE)
setwd("$core/RUNS/$RunName/TMP/$i")
myGAPIT <- GAPIT(Y=myY, GD=myGD,GM=myGM, KI=myKI, group.from=2853,
group.to=2853, Geno.View.output=FALSE)
q()
`;
           close $rfile;
           my $command = "$R/Rscript $top/RUNS/$RunName/TMP/$i/R.txt >
nul 2>\&1";
           system("$command");
           print "Chromosome $i complete!\n";
           exit;

     }

}

while (1) {

  my $child = waitpid(-1, 0);
  last if $child == -1;

}

}

print "MLM Complete! Compiling results...\n"; #Compiles GWAS Results

open my $rfile, '>', "$top/RUNS/$RunName/TMP/R1.txt";
my $core = dirname(abs_path($0));

print $rfile qq`library(compiler)
source("$core/COREFILES/GAPIT/gapit_functions.txt")
source("$core/COREFILES/GAPIT/mt.txt")
library(gplots)
library(genetics)
library(EMMREML)
library("scatterplot3d")
library(MASS)
source("$core/COREFILES/GAPIT/manhattan.txt")
library(manhattanly)
library(plotly)
mydataPath.Results.1="$core/RUNS/$RunName/TMP/1/"
mydataPath.Results.2="$core/RUNS/$RunName/TMP/2/"
mydataPath.Results.3="$core/RUNS/$RunName/TMP/3/"
mydataPath.Results.4="$core/RUNS/$RunName/TMP/4/"
mydataPath.Results.5="$core/RUNS/$RunName/TMP/5/"
```

```
mydataPath.Results.6="$core/RUNS/$RunName/TMP/6/"
mydataPath.Results.7="$core/RUNS/$RunName/TMP/7/"
mydataPath.Results.8="$core/RUNS/$RunName/TMP/8/"
mydataPath.Results.9="$core/RUNS/$RunName/TMP/9/"
mydataPath.Results.10="$core/RUNS/$RunName/TMP/10/"
name <- "$trait"
GWAS.Results.1 <-
read.csv(paste(mydataPath.Results.1,"GAPIT..",name,".GWAS.Results.csv"
,sep=""), head=TRUE)
GWAS.Results.2 <-
read.csv(paste(mydataPath.Results.2,"GAPIT..",name,".GWAS.Results.csv"
,sep=""), head=TRUE)
GWAS.Results.3 <-
read.csv(paste(mydataPath.Results.3,"GAPIT..",name,".GWAS.Results.csv"
,sep=""), head=TRUE)
GWAS.Results.4 <-
read.csv(paste(mydataPath.Results.4,"GAPIT..",name,".GWAS.Results.csv"
,sep=""), head=TRUE)
GWAS.Results.5 <-
read.csv(paste(mydataPath.Results.5,"GAPIT..",name,".GWAS.Results.csv"
,sep=""), head=TRUE)
GWAS.Results.6 <-
read.csv(paste(mydataPath.Results.6,"GAPIT..",name,".GWAS.Results.csv"
,sep=""), head=TRUE)
GWAS.Results.7 <-
read.csv(paste(mydataPath.Results.7,"GAPIT..",name,".GWAS.Results.csv"
,sep=""), head=TRUE)
GWAS.Results.8 <-
read.csv(paste(mydataPath.Results.8,"GAPIT..",name,".GWAS.Results.csv"
,sep=""), head=TRUE)
GWAS.Results.9 <-
read.csv(paste(mydataPath.Results.9,"GAPIT..",name,".GWAS.Results.csv"
,sep=""), head=TRUE)
GWAS.Results.10 <-
read.csv(paste(mydataPath.Results.10,"GAPIT..",name,".GWAS.Results.csv
",sep=""), head=TRUE)
Effect.Estimates.1 <-
read.csv(paste(mydataPath.Results.1,"GAPIT..",name,".Allelic_Effect_Es
timates.csv",sep=""), head=TRUE)
Effect.Estimates.2 <-
read.csv(paste(mydataPath.Results.2,"GAPIT..",name,".Allelic_Effect_Es
timates.csv",sep=""), head=TRUE)
Effect.Estimates.3 <-
read.csv(paste(mydataPath.Results.3,"GAPIT..",name,".Allelic_Effect_Es
timates.csv",sep=""), head=TRUE)
Effect.Estimates.4 <-
read.csv(paste(mydataPath.Results.4,"GAPIT..",name,".Allelic_Effect_Es
timates.csv",sep=""), head=TRUE)
```

```
Effect.Estimates.5 <-
read.csv(paste(mydataPath.Results.5,"GAPIT..",name,".Allelic_Effect_Es
timates.csv",sep=""), head=TRUE)
Effect.Estimates.6 <-
read.csv(paste(mydataPath.Results.6,"GAPIT..",name,".Allelic_Effect_Es
timates.csv",sep=""), head=TRUE)
Effect.Estimates.7 <-
read.csv(paste(mydataPath.Results.7,"GAPIT..",name,".Allelic_Effect_Es
timates.csv",sep=""), head=TRUE)
Effect.Estimates.8 <-
read.csv(paste(mydataPath.Results.8,"GAPIT..",name,".Allelic_Effect_Es
timates.csv",sep=""), head=TRUE)
Effect.Estimates.9 <-
read.csv(paste(mydataPath.Results.9,"GAPIT..",name,".Allelic_Effect_Es
timates.csv",sep=""), head=TRUE)
Effect.Estimates.10 <-
read.csv(paste(mydataPath.Results.10,"GAPIT..",name,".Allelic_Effect_E
stimates.csv",sep=""), head=TRUE)
GWAS.Results <- rbind(GWAS.Results.1, GWAS.Results.2, GWAS.Results.3,
GWAS.Results.4, GWAS.Results.5, GWAS.Results.6, GWAS.Results.7,
GWAS.Results.8, GWAS.Results.9, GWAS.Results.10)
Effect.Estimates <- rbind(Effect.Estimates.1, Effect.Estimates.2,
Effect.Estimates.3, Effect.Estimates.4, Effect.Estimates.5,
Effect.Estimates.6, Effect.Estimates.7, Effect.Estimates.8,
Effect.Estimates.9, Effect.Estimates.10)
GWAS.Results <- GWAS.Results[,-ncol(GWAS.Results)]
setwd("$core/RUNS/$RunName")
Conduct.FDR <-
GAPIT.Perform.BH.FDR.Multiple.Correction.Procedure(PWI=GWAS.Results,
FDR.Rate=$bjh_alpha, FDR.Procedure="BH")
GWAS.Results.FDR <- Conduct.FDR\$PWIP
write.table(GWAS.Results.FDR, paste("GAPIT.", name,
".GWAS.Results.txt", sep = ""), quote = FALSE, sep = "\t", row.names =
FALSE, col.names = TRUE)
write.table(Effect.Estimates, paste("GAPIT.", name,
".Allelic_Effect_Estimates.txt", sep = ""), quote = FALSE, sep = "\t",
row.names = FALSE, col.names = TRUE)
`;

close $rfile;
my $command = "$R/Rscript $top/RUNS/$RunName/TMP/R1.txt > nul 2>\&1";
system("$command");

move("$top/RUNS/$RunName/GAPIT.$trait.Allelic_Effect_Estimates.txt",
"$top/RUNS/$RunName/$trait"."_Effects.txt");
move("$top/RUNS/$RunName/GAPIT.$trait.GWAS.Results.txt",
"$top/RUNS/$RunName/$trait"."_StatisticsTMP.txt");
```

```
open my $in, '<', "$top/RUNS/$RunName/$trait"."_StatisticsTMP.txt";
#Creates a new statistics file sorted by corrected FDR P-value
my $discard = <$in>;
my @lines = <$in>;
chomp @lines;
close $in;

my @ordered_lines = sort { (split("\t", $a))[1] <=> (split("\t",
$b))[1] || (split("\t", $a))[2] <=> (split("\t", $b))[2] } @lines;

open my $out, '>', "$top/RUNS/$RunName/$trait"."_Statistics.txt";
for (@ordered_lines) { print $out "$_\n"; }
close $out;

my @BJH; #Pushes to @BJH FDR P-values below $bjh_alpha, these hits are
colored in the final Manhattan plot
open $in,  '<',  "$top/RUNS/$RunName/$trait"."_Statistics.txt";

while (<$in>) {

     #if ((split("\t", $_))[8] < $bjh_alpha) { #bjh-corrected p-value
below BJH threshold
     if ((split("\t", $_))[3] < $pcalc[(split("\t", $_))[1]-1]) {
#uncorrected p-value below Bonferroni threshold

          push @BJH, (split("\t", $_))[0];

     }

}

close $in;
my $R_HITS = join('","', @BJH);

#Assimilate the final interactive Manhattan plots
open $rfile, '>', "$top/RUNS/$RunName/TMP/R2.txt";
print $rfile qq`capture.output()
setwd("$core/RUNS/$RunName")
name <- "$trait"
library(compiler)
source("$core/COREFILES/GAPIT/gapit_functions.txt")
source("$core/COREFILES/GAPIT/mt.txt")
library(gplots)
library(genetics)
library(EMMREML)
library("scatterplot3d")
library(MASS)
source("$core/COREFILES/GAPIT/manhattan.txt")
```

```
library(manhattanly)
library(plotly)
GWAS.Results <- read.table(paste("$core/RUNS/$RunName/$trait",
"_StatisticsTMP.txt", sep = ""), head=TRUE)
SNPsOfInterest <- c("$R_HITS")
GAPIT.QQ(P.values = GWAS.Results[,4], name.of.trait = name,DPP=50000)
manhattan(GWAS.Results, genomewideline=$avg_Y, chr="Chromosome",
bp="Position", p="P.value", snp="SNP", col=c("black", "gold3"), main =
"Manhattan Plot for $trait", highlight = SNPsOfInterest)
manhattan(subset(GWAS.Results, Chromosome==1), chr="Chromosome",
bp="Position", p="P.value", snp="SNP", col=c("black"),
genomewideline=$b1, main = "Manhattan Plot for $trait", highlight =
SNPsOfInterest)
manhattan(subset(GWAS.Results, Chromosome==2), chr="Chromosome",
bp="Position", p="P.value", snp="SNP", col=c("black"),
genomewideline=$b2, main = "Manhattan Plot for $trait", highlight =
SNPsOfInterest)
manhattan(subset(GWAS.Results, Chromosome==3), chr="Chromosome",
bp="Position", p="P.value", snp="SNP", col=c("black"),
genomewideline=$b3, main = "Manhattan Plot for $trait", highlight =
SNPsOfInterest)
manhattan(subset(GWAS.Results, Chromosome==4), chr="Chromosome",
bp="Position", p="P.value", snp="SNP", col=c("black"),
genomewideline=$b4, main = "Manhattan Plot for $trait", highlight =
SNPsOfInterest)
manhattan(subset(GWAS.Results, Chromosome==5), chr="Chromosome",
bp="Position", p="P.value", snp="SNP", col=c("black"),
genomewideline=$b5, main = "Manhattan Plot for $trait", highlight =
SNPsOfInterest)
manhattan(subset(GWAS.Results, Chromosome==6), chr="Chromosome",
bp="Position", p="P.value", snp="SNP", col=c("black"),
genomewideline=$b6, main = "Manhattan Plot for $trait", highlight =
SNPsOfInterest)
manhattan(subset(GWAS.Results, Chromosome==7), chr="Chromosome",
bp="Position", p="P.value", snp="SNP", col=c("black"),
genomewideline=$b7, main = "Manhattan Plot for $trait", highlight =
SNPsOfInterest)
manhattan(subset(GWAS.Results, Chromosome==8), chr="Chromosome",
bp="Position", p="P.value", snp="SNP", col=c("black"),
genomewideline=$b8, main = "Manhattan Plot for $trait", highlight =
SNPsOfInterest)
manhattan(subset(GWAS.Results, Chromosome==9), chr="Chromosome",
bp="Position", p="P.value", snp="SNP", col=c("black"),
genomewideline=$b9, main = "Manhattan Plot for $trait", highlight =
SNPsOfInterest)
manhattan(subset(GWAS.Results, Chromosome==10), chr="Chromosome",
bp="Position", p="P.value", snp="SNP", col=c("black"),
```

```
genomewideline=$b10, main = "Manhattan Plot for $trait", highlight =
SNPsOfInterest)
GR <- manhattanr(GWAS.Results, chr = "Chromosome", bp = "Position", p
= "P.value")
htmlwidgets::saveWidget(manhattanly(subset(GR[["data"]], CHR %in% 1),
genomewideline=$b1, title = "Manhattan Plot for $trait",
col=c("black")), "Chr_1.html")
htmlwidgets::saveWidget(manhattanly(subset(GR[["data"]], CHR %in% 2),
genomewideline=$b2, title = "Manhattan Plot for $trait",
col=c("black")), "Chr_2.html")
htmlwidgets::saveWidget(manhattanly(subset(GR[["data"]], CHR %in% 3),
genomewideline=$b3, title = "Manhattan Plot for $trait",
col=c("black")), "Chr_3.html")
htmlwidgets::saveWidget(manhattanly(subset(GR[["data"]], CHR %in% 4),
genomewideline=$b4, title = "Manhattan Plot for $trait",
col=c("black")), "Chr_4.html")
htmlwidgets::saveWidget(manhattanly(subset(GR[["data"]], CHR %in% 5),
genomewideline=$b5, title = "Manhattan Plot for $trait",
col=c("black")), "Chr_5.html")
htmlwidgets::saveWidget(manhattanly(subset(GR[["data"]], CHR %in% 6),
genomewideline=$b6, title = "Manhattan Plot for $trait",
col=c("black")), "Chr_6.html")
htmlwidgets::saveWidget(manhattanly(subset(GR[["data"]], CHR %in% 7),
genomewideline=$b7, title = "Manhattan Plot for $trait",
col=c("black")), "Chr_7.html")
htmlwidgets::saveWidget(manhattanly(subset(GR[["data"]], CHR %in% 8),
genomewideline=$b8, title = "Manhattan Plot for $trait",
col=c("black")), "Chr_8.html")
htmlwidgets::saveWidget(manhattanly(subset(GR[["data"]], CHR %in% 9),
genomewideline=$b9, title = "Manhattan Plot for $trait",
col=c("black")), "Chr_9.html")
htmlwidgets::saveWidget(manhattanly(subset(GR[["data"]], CHR %in% 10),
genomewideline=$b10, title = "Manhattan Plot for $trait",
col=c("black")), "Chr_10.html")
rm(GWAS.Results)
rm(Effect.Estimates)
`;

close $rfile;
$command = "$R/Rscript $top/RUNS/$RunName/TMP/R2.txt > nul 2>\&1";
system("$command");

print "Results Compiled! Cleaning...\n"; #Finish sorting out GAPIT
outputs
move("$top/RUNS/$RunName/Rplots.pdf",
"$top/RUNS/$RunName/$trait"."_Manhattan.pdf");
move("$top/RUNS/$RunName/GAPIT.$trait.QQ-Plot.pdf",
"$top/RUNS/$RunName/$trait"."_QQ.pdf");
```

```
unless ($debug == 1) { rmtree("$top/RUNS/$RunName/TMP"); }
print "Results Cleaned! Beginning Interpretation...\n";

my @Hi_Mom;

open $out, '>', "$top/RUNS/$RunName/$trait"."_SignificantHits";
open $in, '<', "$top/RUNS/$RunName/$trait"."_Statistics.txt";
my $head = (<$in>);

print "Selecting Significant Results at FDR p-value < $bjh_alpha or
average Bonferroni p-value < $avg_p (alpha=$bonf_alpha)!\n";
print "Also printing results where p < $p_annotate for
completeness.\n";

while (<$in>) { #Prints Significant Hits to output file

      chomp $_;
      my @names = split("\t", $_);
      my $SNP=$names[0];
      my $Chrom=$names[1];
      my $BP=$names[2];
      my $pval=$names[3];
      my $fdrpval=$names[8];

      #if (($fdrpval < $bjh_alpha) || ($pval < $pcalc[$Chrom-1]) ||
($pval < $p_annotate)) { #must be below BJH, bonferroni, AND user-
specified threshold
      #if ($fdrpval < $bjh_alpha) { #must be below only BJH threshold
      if ($pval < $pcalc[$Chrom-1]) { #must be below only user
threshold

            my $tmpos = join(",",$SNP,$Chrom,$BP,$pval,$fdrpval);
            chomp $tmpos;
            print $out "$tmpos\n";

      }

}

close $out;
close $in;

#Find maize gene names from significant hit marker positions
open $in, '<', "$top/RUNS/$RunName/$trait"."_SignificantHits";
open my $finalarabidopsis, '>',
"$top/RUNS/$RunName/$trait"."_Arabidopsis.txt";
open my $finalrice, '>', "$top/RUNS/$RunName/$trait"."_Rice.txt";
```

```
print $finalarabidopsis "SNP\tChromosome\tLocus\tMLM p-value\tBJH p-value\tGene Midpoint\tGene Distance\tType\tMaize Gene\tArabidopsis Gene\tDescription\tBLAST Alignent Score\tE-value\n";
print $finalrice "SNP\tChromosome\tLocus\tMLM p-value\tBJH p-value\tGene Midpoint\tGene Distance\tType\tMaize Gene\tRice Gene\tDescription\tBLAST Alignent Score\tE-value\n";

while (<$in>) {

      chomp $_;
      my @gwaspos = split(',',$_);
      my $SNP=$gwaspos[0];
      my $chromosome=$gwaspos[1];
      my $posit=$gwaspos[2];
      my $pval=$gwaspos[3];
      my $fdrpval=$gwaspos[4];

      my @genelist = split("\t", $mappings{$SNP});
      for my $gene (@genelist) {

            if ($use_ld == 1) {

                  my $startdistance = abs($start{$gene} - $posit);
                  my $stopdistance = abs($stop{$gene} - $posit);
                  unless (($startdistance <= $ld_window) || ($stopdistance <= $ld_window)) { next; }

            }

            my $midpoint = $midpoints{$gene};
            my $distance = $midpoint-$posit;
            my $type = $nonprot{$gene} // "gene";
            my $name = $known{$gene} // "none";
            my $rice = "none\tnone\tnone\tnone";
            my $arabidopsis = "none\tnone\tnone\tnone";

            if (exists($arabidopsis{$gene})) {

                  $arabidopsis = $arabidopsis{$gene};

            }

            if (exists($rice{$gene})) {

                  $rice = $rice{$gene};

            }
```

```
          print $finalarabidopsis
"$SNP\t$chromosome\t$posit\t$pval\t$fdrpval\t$midpoint\t$distance\t$ty
pe\t$gene\t$name\t$arabidopsis\n";
          print $finalrice
"$SNP\t$chromosome\t$posit\t$pval\t$fdrpval\t$midpoint\t$distance\t$ty
pe\t$gene\t$name\t$rice\n";

     }

     print $finalarabidopsis "\n";
     print $finalrice "\n";

}

close $in;
close $finalarabidopsis;
close $finalrice;

print "Final Report Assembled!\n";

unless ($debug == 1) {

     unlink "$top/RUNS/$RunName/$trait"."_StatisticsTMP.txt";

}

mkdir "$top/RUNS/$RunName/INTERMEDIATES";
move("$top/RUNS/$RunName/$trait"."_Statistics.txt",
"$top/RUNS/$RunName/INTERMEDIATES/$trait"."_Statistics.txt");
move("$top/RUNS/$RunName/$trait"."_Effects.txt",
"$top/RUNS/$RunName/INTERMEDIATES/$trait"."_Effects.txt");
move("$top/RUNS/$RunName/$trait"."_SignificantHits",
"$top/RUNS/$RunName/INTERMEDIATES/$trait"."_SignificantHits.txt");

open $out, '>',
"$top/RUNS/$RunName/INTERMEDIATES/$trait"."_Significance_Thresholds.tx
t";
print $out "Bonferroni Alpha: $bonf_alpha\nFDR-corrected p-value
Threshold: $bjh_alpha\nAverage Bonferroni Threshold p-value:
$avg_p\nAverage Bonferroni Threshold Y-value: $avg_Y\n";
close $out;

print "Cleanup complete!\n";

}

print "GWAS PIPELINE COMPLETE!\n";
```

```
print "Press ENTER to Exit.\n>";
my $DONE = <>;
```

Dataset 2, Script 3

```
#configuration options start at line 52 for:
#    threshold options for selecting candidate significant markers. 
which of these options to use is defined at lines 617-618 and 680-682
#    method for assigning markers to genes-- specified LD window or n 
closest genes to marker

#more in-depth customization:
#    number of markers in datasets 2.7 and 3.2.1 at lines 65-66 and 
328-337
#    GAPIT model defined at lines 555, together with surrounding 
script. For example, enable compression by setting the group.from 
parameter to 0, and/or by adding a group.to parameter to adjust the 
groupings.

#STRUCTURE
#SD2/FOCUS/
#├── SD2-S3.pl #script to execute COMPILE
#├── input *.txt file(s) containing phenotype data
#├── RUNS/
#|    ├── Example.txt #contains example input data in correct format, 
for FOCUS also specify regions to execute GWAS in
#|    └── RESULTS_FOLDERS #one per input data file
#|              ├── *_DATA.txt #copy of original input phenotype data
#|              └── RESULTS_SUBFOLDERS #one per chromosome region 
specified in input file
#|                    ├── INTERMEDIATES/ #raw GAPIT output files, 
including some not used by COMPILE (e.g. reports of marker effect 
size)
#|                    ├── output *_Significance_Thresholds.txt #report 
of significance thresholds used for candidate selection
#|                    ├── output *_Manhattan.pdf #Manhattan plots of 
results
#|                    ├── output *_QQ.pdf #quantile-quantile plot of 
results
#|                    ├── output *_Arabidopsis #candidate gene report 
matching maize to arabidopsis genes
#|                    └── output *_Rice #candidate gene report 
matching maize to rice genes
#└── COREFILES/
#    ├── GAPIT/ #contains files used by GAPIT to execute GWAS
#    └── GENOME/
#         ├── 2.7/ #marker data for Goodman 282 low-density markers
#         |    ├── 1.GD.txt - 10.GD.txt, 1.GM.txt - 10.GM.txt 
#marker data in GAPIT numerical format (SD1-S2, SD1-S3)
#         |    ├── 1k.txt - 10k.txt #kinship matrix data (SD1-S4)
#         |    └── taxa.txt #list of taxa
```

```
#           ├── 3.2.1/ #marker data for Goodman 282 high-density
markers
#           |     ├── 1.GD.txt - 10.GD.txt, 1.GM.txt - 10.GM.txt
#marker data in GAPIT numerical format (SD1-S2, SD1-S3)
#           |     ├── 1k.txt - 10k.txt #kinship matrix data (SD1-S4)
#           |     └── taxa.txt #list of taxa
#           ├── GeneNameList.gff3 #filtered gene info list (SD1-S5)
#           ├── GenePositions.txt #list of gene positions (SD1-S6)
#           ├── KnownGenes.txt #list of known genes in maize
#           ├── taxa.txt #list of taxa
#           ├── Nearest.txt #atlas of nearest ten genes to each marker
(SD1-S7)
#           └── Rice.txt, Arabidopsis.txt #sequence similarity
databases relating maize to rice and arabidopsis (SD1-S8, SD1-S9)

use strict;
use warnings;
use File::Basename;
use Cwd qw(abs_path);
use File::Copy;
use File::Path;
use List::Util qw(sum);
use POSIX qw(ceil);
use Number::Closest::NonOO qw(find_closest_number
find_farthest_number);

my $debug = 0; #If 1, doesn't delete temporary files or directories
my $bjh_alpha = 0.1; #Significance threshold for BJH FDR p-val
my $bonf_alpha = 0.1; #Significance threshold for Bonferroni alpha
my $p_annotate = 0.0001; #Significance threshold to annotate markers
anyway

my $use_ld = 0; #whether to use number of close genes or LD window.
$number_of_close_genes option will still function, e.g. if >1 gene
within the LD window, so set appropriately.
my $number_of_close_genes = 10; #number of genes near each marker to
annotate (max 10)
my $ld_window = 10000; #window in bp around each marker to find genes
to annotate

my $type = fileparse_set_fstype("Unix"); #Allows spaces in file paths
my $dirname = dirname(__FILE__);
my $top = defined($dirname) ? $dirname : '.';

my @num_markers_27 =
(49384,37798,36146,29340,36591,24611,26431,26111,23166,20905);
```

```
my @num_markers_321 =
(3165069,2498723,2436629,2374196,2014626,1609935,1603364,1679038,17024
87,1466141);

my @bonferroni_27;
my @pcalc_27;

for my $nm (@num_markers_27) {

	push @pcalc_27, $bonf_alpha/$nm; #Y-values for significance
thresholds on plot
	my $x = -(log($bonf_alpha/$nm)/log(10));
	push @bonferroni_27, $x;

}

my @bonferroni_321;
my @pcalc_321;

for my $nm (@num_markers_321) {

	push @pcalc_321, $bonf_alpha/$nm;
	my $x = -(log($bonf_alpha/$nm)/log(10));
	push @bonferroni_321, $x;

}

print "Initializing, please wait a moment...\n";

my %nonprot;
open my $gff, '<', "$top/COREFILES/GENOME/GeneNameList.gff3";
while (<$gff>) {

	chomp $_;
	my @columns = split("\t", $_);
	if ($columns[1] ne 'gene') { $nonprot{$columns[4]} = $columns[1];
}

}

close $gff;

my %start;
my %stop;
if ($use_ld == 1) {

	open my $gff, '<', "$top/COREFILES/GENOME/GeneNameList.gff3";
	while (<$gff>) {
```

```
          chomp $_;
          my @columns = split("\t", $_);

          if ($columns[1] eq 'gene') {

               my $name = substr($columns[4], 0, -5);
               if ((!(exists($start{$name}))) || ($columns[2] <
$start{$name})) { $start{$name} = $columns[2]; }
               if ((!(exists($stop{$name}))) || ($columns[3] >
$stop{$name}))  { $stop{$name} = $columns[3]; }

          } else {

               if ((!(exists($start{$columns[4]}))) || ($columns[2] <
$start{$columns[4]})) { $start{$columns[4]} = $columns[2]; }
               if ((!(exists($stop{$columns[4]}))) || ($columns[3] >
$stop{$columns[4]}))  { $stop{$columns[4]} = $columns[3]; }

          }

     }

}

my %midpoints;
open my $mid, '<', "$top/COREFILES/GENOME/GenePositions.txt";
while (<$mid>) {

     chomp $_;
     my @columns = split("\t", $_);
     $midpoints{$columns[2]} = ceil($columns[1]) ;

}

close $mid;

my %mappings;
open my $map, '<', "$top/COREFILES/GENOME/Nearest.txt";
while (<$map>) {

     chomp $_;
     my @columns = split("\t", $_);
     $mappings{$columns[0]} = join("\t",@columns[1..10]);

}

close $map;
```

```
my %known;
open my $knownfile, '<', "$top/COREFILES/Genome/KnownGenes.txt";
my $header = <$knownfile>;
while (<$knownfile>) {

      chomp $_;
      my @columns = split("\t", $_);
      $known{$columns[0]} = join('/',@columns[1..2]);

}

close $knownfile;

my %rice;
open my $ricefile, '<', "$top/COREFILES/Genome/Rice.txt";
my $header1 = <$ricefile>;
while (<$ricefile>) {

      chomp $_;
      my @columns = split("\t", $_);
      $rice{$columns[0]} = join("\t",@columns[1..$#columns]);

}

close $ricefile;

my %arabidopsis;
open my $arabidopsisfile, '<',
"$top/COREFILES/Genome/Arabidopsis.txt";
my $header2 = <$arabidopsisfile>;
while (<$arabidopsisfile>) {

      chomp $_;
      my @columns = split("\t", $_);
      $arabidopsis{$columns[0]} = join("\t",@columns[1..$#columns]);

}

close $arabidopsisfile;

my @AoH;
open my $translate, '<', "$top/COREFILES/Genome/GenePositions.txt";

while (<$translate>) {

      chomp $_;
      my @columns = split("\t", $_);
```

```
	$AoH[$columns[0]]{$columns[1]} = $columns[2];

}

close $translate;

open my $RPath, '<', "$top/COREFILES/R_Installation.txt";
my $R = <$RPath>;
close $RPath;

print "Please place copies of your input data in .txt format in the
base Pipeline folder.\nThe Pipeline will run them sequentially.\nData
files will be renamed and moved to the run folder in /RUNS/.\n";
print "Please ensure the input file is in the format in
/RUNS/Template.txt. Coordinates are accepted in units of
megabases.\nPress any key to continue.\n>";
my $GO = <>;

my @datafiles = glob("$top/*.txt");
for my $file (@datafiles) { #Executes the Pipeline for every set of
trait data in the folder

	opendir my $dh, "$top/RUNS"; #Gets the name of every existing
directory in RUNS
	my @existing_runs = grep {-d "$top/RUNS/$_" && ! /^\.{1,2}$/}
readdir($dh);
	closedir $dh;

	open my $traitfile, '<', "$file"; #Gets the first line of the
input file
	my $firstline = <$traitfile>;

	my @queries;
	my @phenotypes;

	my $trait;
	while (<$traitfile>) {

		chomp $_;
		if ($_  =~ m/Taxa\t(.*?)$/) {

			$trait = $1;
			while (<$traitfile>) {

				push(@phenotypes, $_);

			}
```

```
          }

     else { push(@queries, $_); }

     }

close $traitfile;
chomp $trait;
print "Working on queries for $trait!\n";
chomp @queries;
my $traitund = "$trait"."__";

my $numfix = 1; #Finds how many runs for this trait already exist in
RUNS and add 1 to the suffix
for my $run (@existing_runs) {

     my $suffx = (split('__',$run))[1];
     if ($run =~ m/$traitund/) {

          $numfix = $suffx+1;

     }

}

my $RunName = join('__', $trait,$numfix);
mkdir "$top/RUNS/$RunName", 0755; #Creates and populates the run
directory
my $RunDataName = join('_', $trait,'DATA');
copy("$file", "$top/RUNS/$RunName/$RunDataName.txt");

my @missing;
chomp @phenotypes;
open my $phenos, '>', "$top/RUNS/$RunName/$RunDataName.txt";
print $phenos "Taxa\t$trait\n";
foreach(@phenotypes) {

     print $phenos "$_\n";
     if ($_ =~ m/NaN/) { push @missing, (split("\t", $_))[0]; }

}

close $phenos;
chomp @missing;

my @versions = ("2.7","3.2.1");

for my $version (@versions) {
```

```
print "\tWorking on Version $version...\n";

open my $reference, '<', "$top/COREFILES/GENOME/$version/taxa.txt";
#Nab the taxa list
my @columns = split("\t", <$reference>); #Get an array of taxa names
in the marker data file
close $reference;
chomp @columns;

my @keep; #Defines an array of taxa names to keep

for my $j (0..$#columns) { #Pushes only taxa for which there is data
to @keep by looping through @missing for each value in @columns

      my $flg = 0;
      for my $k (0..$#missing) {

            if ($missing[$k] eq $columns[$j]) { $flg = 1; } #If the
taxon is missing, set the flag to 0 so it doesn't get pushed to @keep

      }

      if ($flg == 0) { push @keep, $columns[$j]; }

}

for my $q (@queries) {

my $input_type;
my $position1;
my $position2;
my @local_keep = @keep;

my $bon;
my $threshold;
my @final_markers;
my $compression_taxa;

my $chromosome = (split("\t", $q))[0];
      next unless ($chromosome =~ /[\d]/);

if ($version eq '2.7') {

      $bon = $pcalc_27[$chromosome-1];
      $threshold = $bonferroni_27[$chromosome-1];
      @final_markers =
(0,306971061,244417267,235520195,246943212,223706202,173351536,1821294
97,181044202,159696575,150890390);
```

```
      $compression_taxa = 282;

}

elsif ($version eq '3.2.1') {

      $bon = $pcalc_321[$chromosome-1];
      $threshold = $bonferroni_321[$chromosome-1];
      @final_markers =
(0,306970776,244440046,235653210,246967124,223706459,173377089,1817188
95,181046086,159687194,150930636);
      $compression_taxa = 279;

}

my $posinput = (split("\t", $q))[1];
if ($posinput !~ /-/) { $input_type = "Single" }
else { $input_type = "Range" }

if ($input_type eq "Single") {

      my $suffix_type;
      my $position;

      $position = $posinput*1000000;

      if ($position >= $final_markers[$chromosome]) {

            $position1 = $final_markers[$chromosome]-10000001;
            $position2 = $final_markers[$chromosome]-1;

      }

      else {

            $position1 = $position-5000000;
            $position2 = $position+5000000;

      }

}

if ($input_type eq "Range") {

      my $pos1 = (split("-", $posinput))[0];
      chomp $pos1;
      $position1 = $pos1*1000000;
```

```
      if ($position1 >= $final_markers[$chromosome]) {

      $position1 = $final_markers[$chromosome]-1;

      }

      my $pos2 = (split("-", $posinput))[1];
      chomp $pos2;
      $position2 = $pos2*1000000;

      if ($position2 >= $final_markers[$chromosome]) {

      $position2 = $final_markers[$chromosome]-1;

      }

      if ($position1 == $position2) { $position1 = $position1-10000000;
}

      my @positions = ($position1,$position2);
      my @sorted_positions = sort { $a <=> $b } @positions;
      $position1 = $sorted_positions[0];
      $position2 = $sorted_positions[1];

}

mkdir "$top/RUNS/$RunName/$version-$chromosome-$posinput", 0755;
mkdir "$top/RUNS/$RunName/$version-$chromosome-$posinput/TMP", 0755;

my $pos1MB = $position1/1000000;
my $pos2MB = $position2/1000000;

open my $manhattan, '<', "$top/COREFILES/GAPIT/manhattan.txt";
my $file_content = do { local $/; <$manhattan> };
open my $local_manhattan, '>', "$top/RUNS/$RunName/$version-
$chromosome-$posinput/TMP/manhattan.txt";
$file_content =~ s/insertxmin/xmin = $pos1MB/;
$file_content =~ s/insertxmax/xmax = $pos2MB/;
print $local_manhattan $file_content;
close $manhattan;
close $local_manhattan;

open my $index, '<', "COREFILES/GENOME/$version/$chromosome.GM.txt";
my $header = <$index>;

my $index_first;
my $index_last;
my $counter = 0;
```

```
while (<$index>) {

      my $position = (split("\t", $_))[2];
      chomp $position;

      if ($position1 >= $position) {

            $counter++;
            next;

      }

      elsif ($position1 <= $position) {

            $index_first = $counter;
            $counter++;
            last;

      }

}

while (<$index>) {

      my $position = (split("\t", $_))[2];
      chomp $position;

      if ($position2 >= $position) {

            $counter++;
            next;

      }

      elsif ($position2 <= $position) {

            $index_last = $counter;
            $counter++;
            last;

      }

}

close $index;
```

```
my $taxa = scalar(@keep); #Number of taxa is equal to the number of
kept columns
my %to_keep = map { $_ => 1 } @keep;

print "\t\tExecuting MLM via GAPIT for Chromosome $chromosome &
$posinput ($taxa Individuals)...\n";

open my $in, '<', "COREFILES/GENOME/$version/$chromosome.GD.txt";
open my $out, '>', "$top/RUNS/$RunName/$version-$chromosome-
$posinput/TMP/$chromosome.GD.txt";

my $firstline = <$in>;
my @firstlinecolumns = split("\t", $firstline);
my @headeroutput;

push @headeroutput, $firstlinecolumns[0];

for my $headernum ($index_first+1..$index_last+1) {

     push @headeroutput, $firstlinecolumns[$headernum];

}

my $GDHead = join("\t", @headeroutput);
print $out "$GDHead\n";

while (<$in>) {

     chomp $_;


     my $taxon = (split("\t", $_))[0];
     if(exists($to_keep{$taxon})) {

          my @cols = split("\t", $_);

          my @output;
          push @output, $cols[0];
          for my $number ($index_first+1..$index_last+1) {

               push @output, $cols[$number];

          }

          my $outline = join("\t", @output);
          print $out "$outline\n";

     }
```

```
}

open $in, '<', "COREFILES/GENOME/$version/$chromosome.GM.txt";
open $out, '>', "$top/RUNS/$RunName/$version-$chromosome-
$posinput/TMP/$chromosome.GM.txt";
$header = <$in>;
print $out $header;

my @GM_lines = <$in>;
close $in;

for my $number ($index_first..$index_last) {

     print $out $GM_lines[$number];

}

close $out;

my $k = "$chromosome"."k";
my $I = $chromosome;

open my $rfile, '>', "$top/RUNS/$RunName/$version-$chromosome-
$posinput/TMP/R1.txt";
my $core = dirname(abs_path($0));

$core =~ s|\\|/|g;
$core =~ s|(.*?)/Pipeline.pl|$1|g;

print $rfile qq`library(compiler)
source("$core/COREFILES/GAPIT/gapit_functions.txt")
source("$core/COREFILES/GAPIT/emma.txt")
source("$core/COREFILES/GAPIT/mt.txt")
library(gplots)
library(LDheatmap)
library(genetics)
library(EMMREML)
library(scatterplot3d)
myGM <- read.table("$core/RUNS/$RunName/$version-$chromosome-
$posinput/TMP/$chromosome.GM.txt", head=TRUE)
myGD <- read.table("$core/RUNS/$RunName/$version-$chromosome-
$posinput/TMP/$chromosome.GD.txt", head=TRUE)
myKI <- read.table("$core/COREFILES/GENOME/$version/$k.txt",
header=FALSE)
myY <- read.table("$core/RUNS/$RunName/$RunDataName.txt", head=TRUE)
setwd("$core/RUNS/$RunName/$version-$chromosome-$posinput/TMP")
```

```
myGAPIT <- GAPIT(Y=myY, GD=myGD, GM=myGM, KI=myKI,
group.from=$compression_taxa, group.to=$compression_taxa,
Geno.View.output=FALSE)
q()
`;

close $rfile;
my $command = "$R/Rscript $top/RUNS/$RunName/$version-$chromosome-
$posinput/TMP/R1.txt > nul 2>\&1";
system("$command");

#Compiles GWAS Results
open $rfile, '>', "$top/RUNS/$RunName/$version-$chromosome-
$posinput/TMP/R2.txt";
print $rfile qq`library(compiler)
source("$core/COREFILES/GAPIT/gapit_functions.txt")
source("$core/COREFILES/GAPIT/mt.txt")
source("$core/RUNS/$RunName/$version-$chromosome-
$posinput/TMP/manhattan.txt")
library(gplots)
library(genetics)
library(EMMREML)
library("scatterplot3d")
library(MASS)
library(manhattanly)
library(plotly)
name <- "$trait"
GWAS.Results <- read.csv("$core/RUNS/$RunName/$version-$chromosome-
$posinput/TMP/GAPIT..$trait.GWAS.Results.csv",head=TRUE)
Effect.Estimates <- read.csv("$core/RUNS/$RunName/$version-
$chromosome-$posinput/TMP/GAPIT..$trait.Allelic_Effect_Estimates.csv",
head=TRUE)
GWAS.Results <- rbind(GWAS.Results)
Effect.Estimates <- rbind(Effect.Estimates)
GWAS.Results <- GWAS.Results[,-ncol(GWAS.Results)]
setwd("$core/RUNS/$RunName/$version-$chromosome-$posinput/TMP")
Conduct.FDR <-
GAPIT.Perform.BH.FDR.Multiple.Correction.Procedure(PWI=GWAS.Results,
FDR.Rate=$bjh_alpha, FDR.Procedure="BH")
GWAS.Results.FDR <- Conduct.FDR\$PWIP
setwd("$core/RUNS/$RunName/$version-$chromosome-$posinput/TMP")
write.table(GWAS.Results.FDR, paste("GAPIT.", name,
".GWAS.Results.txt", sep = ""), quote = FALSE, sep = "\t", row.names =
FALSE, col.names = TRUE)
write.table(Effect.Estimates, paste("GAPIT.", name,
".Allelic_Effect_Estimates.txt", sep = ""), quote = FALSE, sep = "\t",
row.names = FALSE, col.names = TRUE)
q()
```

```
`;

close $rfile;
$command = "$R/Rscript $top/RUNS/$RunName/$version-$chromosome-
$posinput/TMP/R2.txt > nul 2>\&1";
system("$command");

move("$top/RUNS/$RunName/$version-$chromosome-
$posinput/TMP/GAPIT.$trait.Allelic_Effect_Estimates.txt",
"$top/RUNS/$RunName/$version-$chromosome-
$posinput/$trait"."_Effects.txt");
move("$top/RUNS/$RunName/$version-$chromosome-
$posinput/TMP/GAPIT.$trait.GWAS.Results.txt",
"$top/RUNS/$RunName/$version-$chromosome-
$posinput/$trait"."_StatisticsTMP.txt");

open $in, '<', "$top/RUNS/$RunName/$version-$chromosome-
$posinput/$trait"."_StatisticsTMP.txt"; #Creates a new statistics file
sorted by corrected FDR P-value
my $statsheader = <$in>;
my @lines = <$in>;
chomp @lines;
close $in;

my @ordered_lines = sort { (split("\t", $a))[1] <=> (split("\t",
$b))[1] || (split("\t", $a))[2] <=> (split("\t", $b))[2] } @lines;

open $out, '>', "$top/RUNS/$RunName/$version-$chromosome-
$posinput/$trait"."_Statistics.txt";
print $out $statsheader;
for (@ordered_lines) { print $out "$_\n"; }
close $out;

my @BJH; #Pushes to @BJH FDR P-values below $alpha, these hits are
colored in the final Manhattan plot
open $in,  '<',  "$top/RUNS/$RunName/$version-$chromosome-
$posinput/$trait"."_Statistics.txt";
my $discard = (<$in>);

while (<$in>) {

     #if ((split("\t", $_))[8] < $bjh_alpha) { #bjh-corrected p-value
below BJH threshold
     if ((split("\t", $_))[3] < $bon) { #uncorrected p-value below
Bonferroni threshold

            push @BJH, (split("\t", $_))[0];
```

```
      }

}

close $in;
my $R_HITS = join('","', @BJH);

#Assimilate the final interactive Manhattan plots

open $rfile, '>', "$top/RUNS/$RunName/$version-$chromosome-
$posinput/TMP/R3.txt";
print $rfile qq`library(compiler)
setwd("$top/RUNS/$RunName/$version-$chromosome-$posinput")
source("$core/COREFILES/GAPIT/gapit_functions.txt")
source("$core/COREFILES/GAPIT/mt.txt")
library(gplots)
library(genetics)
library(EMMREML)
library("scatterplot3d")
library(MASS)
source("$core/RUNS/$RunName/$version-$chromosome-
$posinput/TMP/manhattan.txt")
library(manhattanly)
library(plotly)
name <- "$trait"
GWAS.Results <- read.table(paste("$core/RUNS/$RunName/$version-
$chromosome-$posinput/$trait", "_StatisticsTMP.txt", sep = ""),
head=TRUE)
SNPsOfInterest <- c("$R_HITS")
GAPIT.QQ(P.values = GWAS.Results[,4], name.of.trait = name,DPP=50000)
manhattan(GWAS.Results, genomewideline=$threshold, chr="Chromosome",
bp="Position", p="P.value", snp="SNP", col=c("black", "gold3"), main =
"Manhattan Plot for $trait", highlight = SNPsOfInterest)
GR <- manhattanr(GWAS.Results, chr = "Chromosome", bp = "Position", p
= "P.value")
htmlwidgets::saveWidget(manhattanly(subset(GR[["data"]], CHR %in%
$chromosome), genomewideline=$threshold, title = "Manhattan Plot for
$trait", col=c("black")), "Chr_$chromosome.html")
q()
`;

close $rfile;
$command = "$R/Rscript $top/RUNS/$RunName/$version-$chromosome-
$posinput/TMP/R3.txt > nul 2>\&1";
system("$command");

#Finish sorting out GAPIT outputs
```

```
move("$top/RUNS/$RunName/$version-$chromosome-$posinput/Rplots.pdf",
"$top/RUNS/$RunName/$version-$chromosome-
$posinput/$trait"."_Manhattan.pdf");
move("$top/RUNS/$RunName/$version-$chromosome-
$posinput/GAPIT.$trait.QQ-Plot.pdf", "$top/RUNS/$RunName/$version-
$chromosome-$posinput/$trait"."_QQ.pdf");

unless ($debug == 1) { rmtree("$top/RUNS/$RunName/$version-
$chromosome-$posinput/TMP"); }

my @Hi_Mom;

open $out, '>', "$top/RUNS/$RunName/$version-$chromosome-
$posinput/$trait"."_SignificantHits";
open $in, '<', "$top/RUNS/$RunName/$version-$chromosome-
$posinput/$trait"."_Statistics.txt";
my $head = (<$in>);

while (<$in>) { #Prints Significant Hits to output file

      chomp $_;
      my @names = split("\t", $_);
      my $SNP=$names[0];
      my $Chrom=$names[1];
      my $BP=$names[2];
      my $pval=$names[3];
      my $fdrpval=$names[8];

      #if (($fdrpval < $bjh_alpha) || ($pval < $bon) || ($pval <
$p_annotate)) { #must be below BJH, bonferroni, AND user-specified
threshold
      #if ($fdrpval < $bjh_alpha) { #must be below only BJH threshold
      if ($pval < $bon) { #must be below only user threshold

            my $tmpos = join(',',$SNP,$Chrom,$BP,$pval,$fdrpval);
            chomp $tmpos;
            print $out "$tmpos\n";

      }

}

close $out;
close $in;


#Find maize gene names from significant hit marker positions
```

```
open $in, '<', "$top/RUNS/$RunName/$version-$chromosome-
$posinput/$trait"."_SignificantHits";
open my $finalarabidopsis, '>', "$top/RUNS/$RunName/$version-
$chromosome-$posinput/$trait"."_Arabidopsis.txt";
open my $finalrice, '>', "$top/RUNS/$RunName/$version-$chromosome-
$posinput/$trait"."_Rice.txt";

print $finalarabidopsis "SNP\tChromosome\tLocus\tMLM p-value\tBJH p-
value\tGene Midpoint\tGene Distance\tType\tMaize Gene\tArabidopsis
Gene\tDescription\tBLAST Alignent Score\tE-value\n";
print $finalrice "SNP\tChromosome\tLocus\tMLM p-value\tBJH p-
value\tGene Midpoint\tGene Distance\tType\tMaize Gene\tRice
Gene\tDescription\tBLAST Alignent Score\tE-value\n";

while (<$in>) {

      chomp $_;
      my @gwaspos = split(',',$_);
      my $SNP=$gwaspos[0];
      my $chromosome=$gwaspos[1];
      my $posit=$gwaspos[2];
      my $pval=$gwaspos[3];
      my $fdrpval=$gwaspos[4];

      my @genelist;

      if ($version eq "2.7") { @genelist = split("\t",
$mappings{$SNP}); }
      elsif ($version eq "3.2.1") {

            my @coords = keys(%{$AoH[$chromosome]});
            @coords = sort { $a <=> $b } @coords;

            my $closest = find_closest_number(number=>$posit,
numbers=>\@coords, items => 10);
            my @values = @{$closest};
            @values = sort @values;

            for my $k (0..9) {

                  push @genelist, $AoH[$chromosome]{$values[$k]};

            }


      }

      for my $gene (@genelist) {
```

```
            if ($use_ld == 1) {

                  my $startdistance = abs($start{$gene} - $posit);
                  my $stopdistance = abs($stop{$gene} - $posit);
                  unless (($startdistance <= $ld_window) ||
($stopdistance <= $ld_window)) { next; }

            }

            my $midpoint = $midpoints{$gene};
            my $distance = $midpoint-$posit;
            my $type = $nonprot{$gene} // "gene";
            my $name = $known{$gene} // "none";
            my $rice = "none\tnone\tnone\tnone";
            my $arabidopsis = "none\tnone\tnone\tnone";

            if (exists($arabidopsis{$gene})) {

                  $arabidopsis = $arabidopsis{$gene};

            }

            if (exists($rice{$gene})) {

                  $rice = $rice{$gene};

            }

            print $finalarabidopsis
"$SNP\t$chromosome\t$posit\t$pval\t$fdrpval\t$midpoint\t$distance\t$ty
pe\t$gene\t$name\t$arabidopsis\n";
            print $finalrice
"$SNP\t$chromosome\t$posit\t$pval\t$fdrpval\t$midpoint\t$distance\t$ty
pe\t$gene\t$name\t$rice\n";

      }

      print $finalarabidopsis "\n";
      print $finalrice "\n";

}

close $in;
close $finalarabidopsis;
close $finalrice;
```

```
unless ($debug == 1) { unlink "$top/RUNS/$RunName/$version-
$chromosome-$posinput/$trait"."_StatisticsTMP.txt"; }

mkdir "$top/RUNS/$RunName/$version-$chromosome-
$posinput/INTERMEDIATES", 0755;
move("$top/RUNS/$RunName/$version-$chromosome-
$posinput/$trait"."_SignificantHits", "$top/RUNS/$RunName/$version-
$chromosome-$posinput/INTERMEDIATES/$trait"."_SignificantHits.txt");
move("$top/RUNS/$RunName/$version-$chromosome-
$posinput/$trait"."_Statistics.txt", "$top/RUNS/$RunName/$version-
$chromosome-$posinput/INTERMEDIATES/$trait"."_Statistics.txt");
move("$top/RUNS/$RunName/$version-$chromosome-
$posinput/$trait"."_Effects.txt", "$top/RUNS/$RunName/$version-
$chromosome-$posinput/INTERMEDIATES/$trait"."_Effects.txt");

open $out, '>', "$top/Runs/$RunName/$version-$chromosome-
$posinput/$trait"."_Significance_Thresholds.txt";
print $out "Bonferroni Alpha: $bonf_alpha\nFDR-corrected p-value
Threshold: $bjh_alpha\nAverage Bonferroni Threshold p-value:
$bon\nAverage Bonferroni Threshold Y-value: $bon\n";
close $out;

}

}

}

print "GWAS PIPELINE COMPLETE!\n";
print "Press ENTER to Exit.\n>";
my $DONE = <>;
```

Dataset 3, Script 1

```
#reanalysis and annotation scripts for GWAS results (will accept any properly formatted files, e.g. from GAPIT)
#useful for re-evaluating significance with new thresholds, or switching between user-defined, Bonferroni, and Benjamini-Hochberg versions and their combinations.

#configuration options start at line 34 for:
#    threshold options for selecting candidate significant markers. which of these options to use is defined at lines 202-204
#    method for assigning markers to genes-- specified LD window or n closest genes to marker

#STRUCTURE
#SD3/REANNOTATE/
#├── SD3-S1.pl #script to re-analyze GAPIT statistics files, generating new lists of significant markers
#├── input *_Statistics.txt file(s) containing GAPIT results
#├── output *_Significance_Thresholds.txt file #significance thresholds
#├── output *_SignificantHits.txt #significant markers
#├── output *_Arabidopsis.txt file #significant gene candidate matches to Arabidopsis
#├── output *_Rice.txt file #significant gene candidate matches to Rice
#└── COREFILES/
#     ├── GOODMAN/1.GM.txt - 10.GM.txt #Goodman panel marker data in GAPIT numerical format (SD1-S2, SD1-S3)
#     ├── NCRPIS/1.GM.txt - 10.GM.txt #NCRPIS panel marker data in GAPIT numerical format (SD1-S2, SD1-S3)
#     ├── 3.2.1/1.GM.txt - 10.GM.txt #Goodman panel high-density marker data in GAPIT numerical format (SD1-S2, SD1-S3)
#     ├── Rice.txt, Arabidopsis.txt #sequence similarity databases relating maize to rice and arabidopsis (SD1-S8, SD1-S9)
#     ├── GeneNameList.gff3 #filtered gene info list (SD1-S5)
#     ├── GenePositions.txt #list of gene positions (SD1-S6)
#     ├── KnownGenes.txt #list of known genes in maize
#     ├── Nearest.txt #atlas of nearest ten genes to each marker (SD1-S7)
#     └── Manhattan.r #template for creating manhattan plot using R package manhattanly

use strict;
use warnings;
use File::Basename;
use List::Util qw(sum);
use POSIX qw(ceil);
```

```
my $version = 'GOODMAN'; #which genome to analyze - either GOODMAN,
NCRPIS, or 3.2.1
my $bjh_alpha = 0.1; #Significance threshold for BJH FDR p-val
my $bonf_alpha = 0.05; #Significance threshold for Bonferroni alpha
my $p_annotate = 0.0001; #Significance threshold to annotate markers
anyway

my $use_ld = 0; #whether to use number of close genes or LD window.
$number_of_close_genes option will still function, e.g. if >1 gene
within the LD window, so set appropriately.
my $number_of_close_genes = 10; #number of genes near each marker to
annotate (max 10)
my $ld_window = 10000; #window in bp around each marker to find genes
to annotate

my $type = fileparse_set_fstype("Unix"); #Allows spaces in file paths
my $dirname = dirname(__FILE__);
my $top = defined($dirname) ? $dirname : '.';

my @num_markers;
for my $i (1..10) { #Get numbers of markers on each chromosome

     open my $file, '<', "$top/COREFILES/$version/$i.GM.txt";
     while (<$file>) {}
     my $line = $.-1; #0-index takes care of -1 for empty line at end;
another -1 for header
     push @num_markers, $line;
     close $file;

}

my @bonferroni;
my @pcalc;
for my $nm (@num_markers) {

     push @pcalc, $bonf_alpha/$nm; #Y-values for significance
thresholds on plot
     my $x = -(log($bonf_alpha/$nm)/log(10));
     push @bonferroni, $x;

}

my $avg_p = sum(@pcalc)/@pcalc;
my $avg_Y = -(log($avg_p)/log(10)); #Average Bonferroni significance
threshold across all 10 chromosomes


my %nonprot;
```

```
open my $gff, '<', "$top/COREFILES/GeneNameList.gff3";
while (<$gff>) {

      chomp $_;
      my @columns = split("\t", $_);
      if ($columns[1] ne 'gene') { $nonprot{$columns[4]} = $columns[1];
}

}

close $gff;

my %start;
my %stop;
if ($use_ld == 1) {

      open my $gff, '<', "$top/COREFILES/GeneNameList.gff3";
      while (<$gff>) {

            chomp $_;
            my @columns = split("\t", $_);

            if ($columns[1] eq 'gene') {

                  my $name = substr($columns[4], 0, -5);
                  if ((!(exists($start{$name}))) || ($columns[2] <
$start{$name})) { $start{$name} = $columns[2]; }
                  if ((!(exists($stop{$name}))) || ($columns[3] >
$stop{$name}))  { $stop{$name} = $columns[3]; }

            } else {

                  if ((!(exists($start{$columns[4]}))) || ($columns[2] <
$start{$columns[4]})) { $start{$columns[4]} = $columns[2]; }
                  if ((!(exists($stop{$columns[4]}))) || ($columns[3] >
$stop{$columns[4]}))  { $stop{$columns[4]} = $columns[3]; }

            }

      }

}

my %midpoints;
open my $mid, '<', "$top/COREFILES/GenePositions.txt";
while (<$mid>) {

      chomp $_;
```

```
      my @columns = split("\t", $_);
      $midpoints{$columns[2]} = ceil($columns[1]) ;

}

close $mid;


my %mappings;
open my $map, '<', "$top/COREFILES/Nearest.txt";
while (<$map>) {

      chomp $_;
      my @columns = split("\t", $_);
      if ($number_of_close_genes > 10) { $number_of_close_genes = 10; }
      $mappings{$columns[0]} =
join("\t",@columns[1..$number_of_close_genes]);

}

close $map;

my %known;
open my $knownfile, '<', "$top/COREFILES/KnownGenes.txt";
my $header = <$knownfile>;
while (<$knownfile>) {

      chomp $_;
      my @columns = split("\t", $_);
      $known{$columns[0]} = join('/',@columns[1..2]);

}

close $knownfile;

my %rice;
open my $ricefile, '<', "$top/COREFILES/Rice.txt";
my $header1 = <$ricefile>;
while (<$ricefile>) {

      chomp $_;
      my @columns = split("\t", $_);
      $rice{$columns[0]} = join("\t",@columns[1..$#columns]);

}

close $ricefile;
```

```
my %arabidopsis;
open my $arabidopsisfile, '<', "$top/COREFILES/Arabidopsis.txt";
my $header2 = <$arabidopsisfile>;
while (<$arabidopsisfile>) {

      chomp $_;
      my @columns = split("\t", $_);
      $arabidopsis{$columns[0]} = join("\t",@columns[1..$#columns]);

}

close $arabidopsisfile;

my @Stats = glob("$top/*Statistics.txt");

for my $file (@Stats) {

$file =~ m/(.*?)_Statistics.txt/;
my $trait = $1;

open my $in, '<', "$top/$trait"."_Statistics.txt";
open my $out, '>', "$top/$trait"."_SignificantHits.txt";
my $head = (<$in>);

while (<$in>) { #Prints Significant Hits to output file

      chomp $_;
      my @names = split("\t", $_);
      my $SNP=$names[0];
      my $Chrom=$names[1];
      my $BP=$names[2];
      my $pval=$names[3];
      my $fdrpval=$names[8];

      #if (($fdrpval < $bjh_alpha) || ($pval < $pcalc[$Chrom-1]) ||
($pval < $p_annotate)) { #must be below BJH, bonferroni, AND user-
specified threshold
      if ($fdrpval < $bjh_alpha) { #must be below only BJH threshold
      #if ($pval < $pcalc[$Chrom-1]) { #must be below only user
threshold

            my $tmpos = join(",",$SNP,$Chrom,$BP,$pval,$fdrpval);
            chomp $tmpos;
            print $out "$tmpos\n";

      }

}
```

```
close $out;
close $in;

#Find maize gene names from significant hit marker positions
open $in, '<', "$top/$trait"."_SignificantHits.txt";
open my $finalarabidopsis, '>', "$top/$trait"."_Arabidopsis.txt";
open my $finalrice, '>', "$top/$trait"."_Rice.txt";

print $finalarabidopsis "SNP\tChromosome\tLocus\tMLM p-value\tBJH p-
value\tGene Midpoint\tGene Distance\tType\tMaize Gene\tArabidopsis
Gene\tDescription\tBLAST Alignent Score\tE-value\n";
print $finalrice "SNP\tChromosome\tLocus\tMLM p-value\tBJH p-
value\tGene Midpoint\tGene Distance\tType\tMaize Gene\tRice
Gene\tDescription\tBLAST Alignent Score\tE-value\n";

while (<$in>) {

	chomp $_;
	my @gwaspos = split(',',$_);
	my $SNP=$gwaspos[0];
	my $chromosome=$gwaspos[1];
	my $posit=$gwaspos[2];
	my $pval=$gwaspos[3];
	my $fdrpval=$gwaspos[4];

	my @genelist = split("\t", $mappings{$SNP});
	for my $gene (@genelist) {

		if ($use_ld == 1) {

			my $startdistance = abs($start{$gene} - $posit);
			my $stopdistance = abs($stop{$gene} - $posit);
			unless (($startdistance <= $ld_window) ||
($stopdistance <= $ld_window)) { next; }

		}

		my $midpoint = $midpoints{$gene};
		my $distance = $midpoint-$posit;
		my $type = $nonprot{$gene} // "gene";
		my $name = $known{$gene} // "none";
		my $rice = "none\tnone\tnone\tnone";
		my $arabidopsis = "none\tnone\tnone\tnone";

		if (exists($arabidopsis{$gene})) {

			$arabidopsis = $arabidopsis{$gene};
```

```
          }

          if (exists($rice{$gene})) {

               $rice = $rice{$gene};

          }

          print $finalarabidopsis
"$SNP\t$chromosome\t$posit\t$pval\t$fdrpval\t$midpoint\t$distance\t$ty
pe\t$gene\t$name\t$arabidopsis\n";
          print $finalrice
"$SNP\t$chromosome\t$posit\t$pval\t$fdrpval\t$midpoint\t$distance\t$ty
pe\t$gene\t$name\t$rice\n";

     }

     print $finalarabidopsis "\n";
     print $finalrice "\n";

}

close $in;
close $finalarabidopsis;
close $finalrice;

open $out, '>', "$top/$trait"."_Significance_Thresholds.txt";
print $out "Bonferroni Alpha: $bonf_alpha\nFDR-corrected p-value
Threshold: $bjh_alpha\nAverage Bonferroni Threshold p-value:
$avg_p\nAverage Bonferroni Threshold Y-value: $avg_Y\n";
close $out;

}
```

Dataset 3, Script 2
#reanalysis and annotation scripts for GWAS results (will accept any properly formatted files, e.g. from GAPIT)
#useful for generating Manhattan plots with precisely placed vertical lines at marker or gene locations

```
#SD3/REANNOTATE/
#├── SD3-S2.pl #script to generate annotated Manhattan plots from GAPIT statistics files and list of genes
#├── input *_Statistics.txt file(s) #file containing GAPIT results
#├── input *_SignificantHits.txt file(s) #file containing COMPILE-annotated significant results
#├── input *_Genes.txt file(s) #file containing list of genes to annotate
#├── output *_Manhattan.pdf file #annotated Manhattan plot
#└── COREFILES/
#     ├── GOODMAN/1.GM.txt - 10.GM.txt #Goodman panel marker data in GAPIT numerical format (SD1-S2, SD1-S3)
#     ├── NCRPIS/1.GM.txt - 10.GM.txt #NCRPIS panel marker data in GAPIT numerical format (SD1-S2, SD1-S3)
#     ├── 3.2.1/1.GM.txt - 10.GM.txt #Goodman panel high-density marker data in GAPIT numerical format (SD1-S2, SD1-S3)
#     ├── Rice.txt, Arabidopsis.txt #sequence similarity databases relating maize to rice and arabidopsis (SD1-S8, SD1-S9)
#     ├── GeneNameList.gff3 #filtered gene info list (SD1-S5)
#     ├── GenePositions.txt #list of gene positions (SD1-S6)
#     ├── KnownGenes.txt #list of known genes in maize
#     ├── Nearest.txt #atlas of nearest ten genes to each marker (SD1-S7)
#     └── Manhattan.r #template for creating manhattan plot using R package manhattanly

use strict;
use warnings;
use Cwd qw(abs_path);
use File::Copy;
use File::Path;
use File::Basename;
use List::Util qw(sum);
use POSIX qw(ceil);

my $focus_mode = 0; #whether to analayze input files as regions of chromosomes, as SD2-S3 produces outputs
my $RPath = 'C:/"Program Files"/R/R-4.1.1/bin/x64'; #path to R installation, directories w/ spaces in double quotes
my $version = 'NCRPIS'; #which genome to analyze; either GOODMAN or NCRPIS
my $bjh_alpha = 0.1; #Significance threshold for BJH FDR p-val
```

```
my $bonf_alpha = 0.1; #Significance threshold for Bonferroni alpha

my $type = fileparse_set_fstype("Unix"); #Allows spaces in file paths
my $dirname = dirname(__FILE__);
my $top = defined($dirname) ? $dirname : '.';

my @num_markers;
for my $i (1..10) { #Get numbers of markers on each chromosome

	open my $file, '<', "$top/COREFILES/$version/$i.GM.txt";
	while (<$file>) {}
	my $line = $.-1; #0-index takes care of -1 for empty line at end;
another -1 for header
	push @num_markers, $line;
	close $file;

}

my %last_coords;
for my $i (1..10) {

	open my $fh, '<', "$top/COREFILES/$version/$i.GM.txt";
	my $lastline;
	$lastline = $_ while <$fh>;
	chomp $lastline;
	$last_coords{$i} = (split("\t",$lastline))[2];

}

my %chrom;
my %pos;

for my $i (1..10) {

	open my $fh, '<', "$top/COREFILES/$version/$i.GM.txt";
	my $header = <$fh>;

	while (<$fh>) {

		chomp $_;
		my @columns = split("\t",$_);
		$chrom{$columns[0]} = $columns[1];
		$pos{$columns[0]} = $columns[2];

	}

}
```

```
my %firsts;
my $counter = 1;

for my $i (1..10) {

$firsts{$i} = $counter;
my $foo = $last_coords{$i};
$counter += $last_coords{$i};

}

my @bonferroni;
my @pcalc;
for my $nm (@num_markers) {

	push @pcalc, $bonf_alpha/$nm; #Y-values for significance
thresholds on plot
	my $x = -(log($bonf_alpha/$nm)/log(10));
	push @bonferroni, $x;

}

my $b1 = $bonferroni[0]; #Because R doesn't accept the array value; I
know it looks bad
my $b2 = $bonferroni[1];
my $b3 = $bonferroni[2];
my $b4 = $bonferroni[3];
my $b5 = $bonferroni[4];
my $b6 = $bonferroni[5];
my $b7 = $bonferroni[6];
my $b8 = $bonferroni[7];
my $b9 = $bonferroni[8];
my $b10 = $bonferroni[9];

my $avg_p = sum(@pcalc)/@pcalc;
my $avg_Y = -(log($avg_p)/log(10)); #Average Bonferroni significance
threshold across all 10 chromosomes

my %midpoints;
my %chromosomes;
open my $mid, '<', "$top/COREFILES/GenePositions.txt";
while (<$mid>) {

	chomp $_;
	my @columns = split("\t", $_);
	$midpoints{$columns[2]} = ceil($columns[1]) ;
	$chromosomes{$columns[2]} = $columns[0];
```

```
}

close $mid;

my @Stats = glob("$top/*Statistics.txt");

for my $file (@Stats) {

$file =~ m/\.\/(.*?)_Statistics.txt/;
my $trait = $1;

open my $in,  '<', "$top/$trait"."_Statistics.txt";
open my $out, '>', "$top/$trait"."_StatisticsTMP.txt";
print $out
"SNP\tChromosome\tPosition\tP.value\tx\ttaxa\tx\ty\tFDR.p.value\n";

my @coords;
my $current_chrom;
if ($focus_mode = 0) {

      while (<$in>) {

            print $out $_;

      }

} else {

      my $i = 0;
      while (<$in>) {

            push @coords, (split("\t", $_))[2];
            print $out $_;

            if ($i == 0) {

                  $i++;
                  $current_chrom = (split("\t", $_))[1]

            }

      }

}

close $in;
close $out;
```

```
my @sorted_coords;
my $pos1 $pos2;
if ($focus_mode = 1) {

      my @sorted_coords = sort { $a <=> $b } @coords; }
      my $pos1 = shift(@sorted_coords)/1000000;
      my $pos2 = pop(@sorted_coords)/1000000;

      open my $manhattan, '<', "$top/COREFILES/focus_manhattan.r";
      my $file_content = do { local $/; <$manhattan> };
      open my $local_manhattan, '>', "$top/COREFILES/TMP_manhattan.r";
      $file_content =~ s/insertxmin/xmin = $pos1/;
      $file_content =~ s/insertxmax/xmax = $pos2/;
      print $local_manhattan $file_content;
      close $manhattan;
      close $local_manhattan;

}

my @hits;
open my $tmp,  '<', "$top/$trait"."_SignificantHits.txt";

while (<$tmp>) {

      push @hits, (split(',', $_))[0];

}

close $tmp;

my @genes;
if (-e "$top/$trait"."_genes.txt") {
      open my $genelist, '<', "$top/$trait"."_genes.txt";

      while (<$genelist>) {

            chomp $_;
            push @genes, $_;

      }

      close $genelist;

}

my @markers;
if (-e "$top/$trait"."_markers.txt") {
      open my $markerlist, '<', "$top/$trait"."_markers.txt";
```

```
	while (<$markerlist>) {

		chomp $_;
		push @markers, $_;

	}

	close $markerlist;

}

my %strings;
my $total_string;

my $color = qq("red");

if ($focus_mode = 0) {

foreach my $gene (@genes) {

	my $rawposition = $midpoints{$gene};
	my $position = $rawposition/1000000;
	my $chromosome = $chromosomes{$gene};
	my $total_position = $rawposition + $firsts{$chromosome};

	if (exists($strings{$chromosome})) { $strings{$chromosome} =
qq($strings{$chromosome},"$position"); }
	else { $strings{$chromosome} = qq("$position"); }

	if (defined($total_string)) { $total_string =
qq($total_string,"$total_position"); }
	else { $total_string = qq("$total_position"); }

}

foreach my $marker (@markers) {

	my $rawposition = $pos{$marker};
	my $position = $rawposition/1000000;
	my $chromosome = $chrom{$marker};
	my $total_position = $rawposition + $firsts{$chromosome};

	if (exists($strings{$chromosome})) { $strings{$chromosome} =
qq($strings{$chromosome},"$position"); }
	else { $strings{$chromosome} = qq("$position"); }
```

```
      if (defined($total_string)) { $total_string =
qq($total_string,"$total_position"); }
      else { $total_string = qq("$total_position"); }

}

} else {

foreach my $gene (@genes) {

      my $rawposition = $midpoints{$gene};
      my $position = $rawposition/1000000;
      my $chromosome = $chromosomes{$gene};

      if (defined($total_string)) { $total_string =
qq($total_string,"$position"); }
      else { $total_string = qq("$position"); }

}

foreach my $marker (@markers) {

      my $rawposition = $midpoints{$marker};
      my $position = $rawposition/1000000;
      my $chromosome = $chromosomes{$marker};

      if (defined($total_string)) { $total_string =
qq($total_string,"$position"); }
      else { $total_string = qq("$position"); }

}

}

my $string1 = '';
my $string2 = '';
my $string3 = '';
my $string4 = '';
my $string5 = '';
my $string6 = '';
my $string7 = '';
my $string8 = '';
my $string9 = '';
my $string10 = '';

if (defined($total_string)) { $total_string = "$total_string"; }
else { $total_string = ''; }
```

```
if (exists($strings{1})) { $string1 = "$strings{1}"; }
else { $string1 = ''; }

if (exists($strings{2})) { $string2 = "$strings{2}"; }
else { $string2 = ''; }

if (exists($strings{3})) { $string3 = "$strings{3}"; }
else { $string3 = ''; }

if (exists($strings{4})) { $string4 = "$strings{4}"; }
else { $string4 = ''; }

if (exists($strings{5})) { $string5 = "$strings{5}"; }
else { $string5 = ''; }

if (exists($strings{6})) { $string6 = "$strings{6}"; }
else { $string6 = ''; }

if (exists($strings{7})) { $string7 = "$strings{7}"; }
else { $string7 = ''; }

if (exists($strings{8})) { $string8 = "$strings{8}"; }
else { $string8 = ''; }

if (exists($strings{9})) { $string9 = "$strings{9}"; }
else { $string9 = ''; }

if (exists($strings{10})) { $string10 = "$strings{10}"; }
else { $string10 = ''; }

my $R_HITS = join('","', @hits);

my $core = dirname(abs_path($0));
open my $rfile, '>', "$top/R.txt";

if ($focus_mode = 0) {

print $rfile qq`capture.output()
setwd("$core")
name <- "$trait"
library(compiler)
library(gplots)
library(genetics)
library(EMMREML)
library("scatterplot3d")
library(MASS)
source("$core/COREFILES/manhattan.r")
library(manhattanly)
```

```
library(plotly)
GWAS.Results <- read.table(paste("$core/$trait", "_StatisticsTMP.txt",
sep = ""), head=TRUE)
SNPsOfInterest <- c("$R_HITS")
manhattan(GWAS.Results, genomewideline=$avg_Y, chr="Chromosome",
bp="Position", p="P.value", snp="SNP", col=c("black", "gold3"), main =
"Manhattan Plot for $trait", highlight = SNPsOfInterest,
ablines=c($total_string))
manhattan(subset(GWAS.Results, Chromosome==1), chr="Chromosome",
bp="Position", p="P.value", snp="SNP", col=c("black"),
genomewideline=$b1, main = "Manhattan Plot for $trait", highlight =
SNPsOfInterest, ablines=c($string1))
manhattan(subset(GWAS.Results, Chromosome==2), chr="Chromosome",
bp="Position", p="P.value", snp="SNP", col=c("black"),
genomewideline=$b2, main = "Manhattan Plot for $trait", highlight =
SNPsOfInterest, ablines=c($string2))
manhattan(subset(GWAS.Results, Chromosome==3), chr="Chromosome",
bp="Position", p="P.value", snp="SNP", col=c("black"),
genomewideline=$b3, main = "Manhattan Plot for $trait", highlight =
SNPsOfInterest, ablines=c($string3))
manhattan(subset(GWAS.Results, Chromosome==4), chr="Chromosome",
bp="Position", p="P.value", snp="SNP", col=c("black"),
genomewideline=$b4, main = "Manhattan Plot for $trait", highlight =
SNPsOfInterest, ablines=c($string4))
manhattan(subset(GWAS.Results, Chromosome==5), chr="Chromosome",
bp="Position", p="P.value", snp="SNP", col=c("black"),
genomewideline=$b5, main = "Manhattan Plot for $trait", highlight =
SNPsOfInterest, ablines=c($string5))
manhattan(subset(GWAS.Results, Chromosome==6), chr="Chromosome",
bp="Position", p="P.value", snp="SNP", col=c("black"),
genomewideline=$b6, main = "Manhattan Plot for $trait", highlight =
SNPsOfInterest, ablines=c($string6))
manhattan(subset(GWAS.Results, Chromosome==7), chr="Chromosome",
bp="Position", p="P.value", snp="SNP", col=c("black"),
genomewideline=$b7, main = "Manhattan Plot for $trait", highlight =
SNPsOfInterest, ablines=c($string7))
manhattan(subset(GWAS.Results, Chromosome==8), chr="Chromosome",
bp="Position", p="P.value", snp="SNP", col=c("black"),
genomewideline=$b8, main = "Manhattan Plot for $trait", highlight =
SNPsOfInterest, ablines=c($string8))
manhattan(subset(GWAS.Results, Chromosome==9), chr="Chromosome",
bp="Position", p="P.value", snp="SNP", col=c("black"),
genomewideline=$b9, main = "Manhattan Plot for $trait", highlight =
SNPsOfInterest, ablines=c($string9))
manhattan(subset(GWAS.Results, Chromosome==10), chr="Chromosome",
bp="Position", p="P.value", snp="SNP", col=c("black"),
genomewideline=$b10, main = "Manhattan Plot for $trait", highlight =
SNPsOfInterest, ablines=c($string10))
```

```
q()
`;

} else {

print $rfile qq`capture.output()
setwd("$core")
name <- "$trait"
library(compiler)
library(gplots)
library(genetics)
library(EMMREML)
library("scatterplot3d")
library(MASS)
source("$top/COREFILES/TMP_manhattan.r")
library(manhattanly)
library(plotly)
GWAS.Results <- read.table(paste("$core/$trait", "_StatisticsTMP.txt",
sep = ""), head=TRUE)
SNPsOfInterest <- c("$R_HITS")
manhattan(subset(GWAS.Results, Chromosome==$current_chrom),
chr="Chromosome", bp="Position", p="P.value", snp="SNP",
col=c("black"), genomewideline=$bon, main = "Manhattan Plot for
$trait", highlight = SNPsOfInterest, ablines=c($total_string))
q()
`;

}

close $rfile;
my $command = "$RPath/Rscript $top/R.txt";
system("$command");

unlink("$top/$trait"."_StatisticsTMP.txt");
unlink("$top/R.txt");
unlink("$top/COREFILES/TMP_manhattan.r");
move("$top/Rplots.pdf", "$top/$trait"."_Manhattan.pdf");

}
```

Dataset 3, Script 3
#script for overlaying FOCUS dual Manhattan plots with maize local genome architecture

#configuration options are:
#   annotation colors: 32-33
#   .gff3 file for target genome: 51
#   feature coloring for the plot: starts line 200
#   types of featuers to plot: 253-255

#STRUCTURE
#SD3/PLOTTER/
#├── SD3-S3.pl #script to re-analyze GAPIT statistics files, generating new lists of significant markers
#├── input *_Input.txt file(s) containing beginning and end genes to include in plot, each on a separate line
#├── input *_Statistics_2.7.txt file(s) containing GAPIT results from low-density marker data
#├── input *_Statistics_3.2.1.txt file(s) containing GAPIT results from high-density marker data
#├── input *_SignificantHits_2.7.txt file(s) containing COMPILE-annotated significant genes from analysis of low-density marker data
#├── input *_SignificantHits_3.2.1.txt file(s) containing COMPILE-annotated significant genes from analysis of high-density marker data
#├── output *_png.txt file(s) #plot
#└── COREFILES/
#   ├── GENOME/2.7/1.GM.txt - 10.GM.txt #Goodman panel marker data in GAPIT numerical format (SD1-S2, SD1-S3)
#   ├── GENOME/3.2.1/1.GM.txt - 10.GM.txt #Goodman high-density panel marker data in GAPIT numerical format (SD1-S2, SD1-S3)
#   ├── GenePositions.txt #list of gene positions (SD1-S6)
#   └── Zea_mays.B73_RefGen_v4.45.gff3 #gene information for target genome

```
use strict;
use warnings;
use Cwd qw(abs_path);
use File::Copy;
use File::Path;
use File::Basename;
use POSIX;

my $basecolor = "black";
my $accentcolor = "red";

my $type = fileparse_set_fstype("Unix"); #Allows spaces in file paths
my $dirname = dirname(__FILE__);
my $top = defined($dirname) ? $dirname : '.';
```

```
my %chrom;
open my $mid, '<', "$top/COREFILES/GenePositions.txt";
while (<$mid>) {

      chomp $_;
      my @columns = split("\t", $_);
      $chrom{$columns[2]} = $columns[0];

}

close $mid;

my $GFF = "$top/COREFILES/Zea_mays.B73_RefGen_v4.45.gff3";
my @Input = glob("$top/*_Input.txt");

open my $in, '<', $GFF;
$/ = '###';

my %start;
my %stop;
while (<$in>) {

      if ($_ =~ m/ID=gene:(.*?);/) {

            my $gene = $1;
            my @columns = split("\t", $_);

            $start{$gene} = $columns[3];
            $stop{$gene} = $columns[4];

      }

}

close $in;
$/ = "\n";

my %bonferroni27;
my %bonferroni321;
my $bonf_alpha= 0.1;

for my $version ('2.7', '3.2.1') {

      my %bonferroni;
      my @num_markers;
      for my $i (1..10) { #Get numbers of markers on each chromosome
```

```
            open my $file, '<', 
"$top/COREFILES/GENOME/$version/$i.GM.txt";
            while (<$file>) {}
            my $line = $.-1; #0-index takes care of -1 for empty line 
at end; another -1 for header
            push @num_markers, $line;
            close $file;

      }

      my $i = 1;

      for my $nm (@num_markers) {

            my $x = -(log($bonf_alpha/$nm)/log(10));
            $bonferroni{$i} = $x;
            $i++;

      }

      if ($version eq '2.7') { %bonferroni27 = %bonferroni; }
      else { %bonferroni321 = %bonferroni; }

}

for my $file (@Input) {

$file =~ m/\.\/(.*?)_Input.txt/;
my $trait = $1;

open my $in,  '<', $file;

my @one;
my @two;
$/ = "\n\n";
while (<$in>) {

      chomp $_;
      my $first = (split("\n", $_))[0];
      $first =~ s/\R//g;
      push @one, $first;
      my $second = (split("\n", $_))[1];
      $second =~ s/\R//g;
      push @two, $second;

}

close $in;
```

```
$/ = "\n";

for my $version ('2.7', '3.2.1') {

      $/ = undef;
      my $stats_string = "SNP    Chromosome Position   P.value    maf
      nobs  Rsquare.of.Model.without.SNP    Rsquare.of.Model.with.SNP
      FDR_Adjusted_P-values\n";
      my $stats_replacement =
"SNP\tChromosome\tposition\tpvalue\tx\ttaxa\tx\ty\tFDR.p.value\n";

      open $in,  '<', "$top/$trait"."_Statistics_$version.txt";
      my $file = <$in>;
      close $in;

      $file =~ s/$stats_string/$stats_replacement/;

      open my $out, '>', "$top/$trait"."_Statistics_$version.txt";
      print $out $file;
      close $out;

      my $sig_string = "SNP,Chromosome,position,pvalue,FDR.p.value\n";

      open $in,  '<', "$top/$trait"."_SignificantHits_$version.txt";
      $file = <$in>;
      close $in;

      unless ($file =~ m/$sig_string/) {

            open my $out, '>',
"$top/$trait"."_SignificantHits_$version.txt";
            print $out $sig_string;
            print $out $file;
            close $out;

      }

}

for my $i (0..$#one) {

my $first = $one[$i];
my $second = $two[$i];

my @coordset =
($start{$first},$stop{$first},$start{$second},$stop{$second});
my @sorted_coords = sort { $a <=> $b } @coordset;
```

```
my $Start = shift(@sorted_coords);
my $Stop = pop(@sorted_coords);
my $Length = $Stop - $Start;
my $Chrom = $chrom{$first};

open $in, '<', $GFF;
$/ = '###';

my $color = '"#ffd700"';

my $pythonname = "python_$trait"."_$i.py";
open my $py, '>', $pythonname;
print $py "import numpy as np\nimport pandas as pd\nfrom pandas import
DataFrame\nimport matplotlib.pyplot as plt\nimport matplotlib.ticker
as tkr\nfrom dna_features_viewer import
(\n\tGraphicFeature,\n\tGraphicRecord,\n)\n\n";
print $py "def numfmt(x,pos):\n\ts = '{}'.format(x /
1000000)\n\treturn s\n\nxfmt = tkr.FuncFormatter(numfmt)\n\n";
print $py "font12 = {'family' : 'Arial',\n\t'color':
'black',\n\t'weight': 'bold',\n\t'size': 12,\n\t}\n\nfont14 =
{'family' : 'Arial',\n\t'color': 'black',\n\t'weight':
'bold',\n\t'size': 14,\n\t}\n\n";
print $py "plt.rcParams[\"axes.linewidth\"] = 1.5\nfig, (ax1, ax2,
ax3) = plt.subplots(\n\t3, figsize = (12, 9), sharex=True,
gridspec_kw={\"height_ratios\": [3, 1, 1], 'hspace': 0}\n)\n\nfeatures
= [\n";

my $sandwichtop = "\tGraphicFeature(\n\t\t";
my $sandwichbottom = "\n\t),\n";
my @annotations;
my %unique;

my %color = (

      #unusual features
      RNase_MRP_RNA => '"#FFFFFF"',
      SRP_RNA => '"#FFFFFF"',
      pseudogene => '"#FFFFFF"',
      ncRNA => '"#FFFFFF"',
      ncRNA_gene => '"#FFFFFF"',

      #RNA features
      rRNA => '"#FF0000"',
      tRNA => '"#FF0000"',
      lnc_RNA => '"#FF0000"',
      pre_miRNA => '"#FF0000"',
      miRNA => '"#FF0000"',
      snRNA => '"#FF0000"',
```

```
	snoRNA => '"#FF0000"',

	#highly relevant features
	five_prime_UTR => '"#00FF00"',
	gene => '"#FFFFFF"',
	exon =>'"#0000FF"',
	CDS => '"#007FFF"', #not plotted
	three_prime_UTR =>  '"#7F00FF"',

	mRNA => '"#000000"', #not plotted

);

sub annotate {

	my %localunique = %unique;
	my @tannotations;
	my $entry = $_[0];
	my @lines = split("\n", $entry);
	my @real_lines;

	for my $line (@lines) {

		unless ($line !~ m/^\d/) { push @real_lines, $line; }

	}

	for my $line (@real_lines) {

		my @columns = split("\t", $line);
		my $type = $columns[2];
		my $beginning = $columns[3];
		my $end = $columns[4];
		my $strand = $columns[6];
		my $annot = $columns[8];
		my $name;

		$annot =~ m/^.*?:(.*?);/;
		$name = $1;

		#if ($type =~ /gene|UTR|exon|CDS|[^m]RNA/) {
		#if ($type =~ /gene|UTR|exon|[^m]RNA/) {
		if ($type =~ /gene|exon|[^m]RNA/) {

			my $ID = "$beginning"."$end"."$type";

			unless((exists($unique{$ID}) ||
(exists($localunique{$ID})))) {
```

```
                    $localunique{$ID} = 1;

                    if ($type =~ /gene/) {

                         unless($type =~ /ncRNA_gene/) {

                              my $label = "$type: "."$name";
                              my $string =
"$sandwichtop"."start=$beginning, end = $end, strand=$strand"."1,
color=$color{$type}, label=\"$label\", linewidth = 1.5, fontdict =
font12"."$sandwichbottom";
                              push @tannotations, $string;

                         }

                    } elsif ($type =~ /[^m]RNA/) {

                         my $label = "$type: "."$name";
                         my $string =
"$sandwichtop"."start=$beginning, end = $end, strand=$strand"."1,
color=$color{$type}, label=\"$label\", linewidth = 1.5, fontdict =
font12"."$sandwichbottom";
                         push @tannotations, $string;

                    } else {

                         my $string =
"$sandwichtop"."start=$beginning, end = $end, strand=$strand"."1,
color=$color{$type}, linewidth = 1.5"."$sandwichbottom";
                         push @tannotations, $string;

                    }

               }

          }

     }

     return \@tannotations, \%localunique;

}

my $flag = 0;
while (<$in>) {

     if ($_ =~ m/ID=gene:(.*?);/) {
```

```
            chomp $_;
            my $gene = $1;

            if ($gene eq $second) {

                  my ($array, $hash) = annotate($_);
                  push @annotations, @$array;
                  %unique = (%unique, %$hash);
                  last;

            }

            if ($flag == 1) {

                  my ($array, $hash) = annotate($_);
                  push @annotations, @$array;
                  %unique = (%unique, %$hash);

            }

            if ($gene eq $first) {

                  $flag = 1;
                  my ($array, $hash) = annotate($_);
                  push @annotations, @$array;
                  %unique = (%unique, %$hash);

            }

      }

}

close $in;

for my $entry (@annotations) {

      print $py $entry;

}

my $buffer_amt = $Length/100;
my $buffernear = $Start-$buffer_amt;
my $bufferfar = $Length+(2*$buffer_amt);

sub magic_number {
   my $n = shift;
```

```
   return (log($n)/log(10))-floor(log($n)/log(10));
}

my $number = magic_number($Length);

my $inc = 1;
if ($number >= magic_number(500)) { $inc = 10 }
elsif ($number >= magic_number(250)) { $inc = 5 }
elsif ($number >= magic_number(125)) { $inc = 2.5 }
my $multiple = (10 ** ((floor(log($Length)/log(10))) -1 )) * $inc;

print $py "]\n\nrecord =
GraphicRecord(sequence_length=$bufferfar,first_index=$buffernear,
features=features)\nrecord.plot(ax=ax1, with_ruler=False,
strand_in_label_threshold=4)\n\n";
print $py "df =
pd.read_csv(\"$trait"."_Statistics_2.7.txt\",sep='\\t',skiprows=(0),us
ecols=[2,3],header=(0))\ndf['minuslog10pval'] = -
np.log10(df.pvalue)\ndf.plot.scatter(x='position', y='minuslog10pval',
color='$basecolor', ax=ax2)\n\n";
print $py "df2 =
pd.read_csv(\"$trait"."_SignificantHits_2.7.txt\",sep=',',skiprows=(0)
,usecols=[2,3],header=(0))\ndf2['minuslog10pval'] = -
np.log10(df2.pvalue)\ndf2.plot.scatter(x='position',
y='minuslog10pval', color='$accentcolor', ax=ax2)\n\n";
print $py "df3 =
pd.read_csv(\"$trait"."_Statistics_3.2.1.txt\",sep='\\t',skiprows=(0),
usecols=[2,3],header=(0))\ndf3['minuslog10pval'] = -
np.log10(df3.pvalue)\ndf3.plot.scatter(x='position',
y='minuslog10pval', color='$basecolor', ax=ax3)\n\n";
print $py "df4 =
pd.read_csv(\"$trait"."_SignificantHits_3.2.1.txt\",sep=',',skiprows=(
0),usecols=[2,3],header=(0))\ndf4['minuslog10pval'] = -
np.log10(df4.pvalue)\ndf4.plot.scatter(x='position',
y='minuslog10pval', color='$accentcolor', ax=ax3)\n\n";
print $py
"ax3.xaxis.set_major_formatter(xfmt)\nax3.xaxis.set_major_locator(plt.
MultipleLocator($multiple))\n\nax2.set_ylim(bottom=0)\nax3.set_ylim(bo
ttom=0)\nax2.set_ylabel(\"-log10(p)\",
fontdict=font14)\nax3.set_ylabel(\"-log10(p)\",
fontdict=font14)\nax3.set_xlabel(\"Chromosome $Chrom Position, Mbp\",
fontdict=font14)\nax2.axhline($bonferroni27{$Chrom},
color=\"$accentcolor\")\nax3.axhline($bonferroni321{$Chrom},
color=\"$accentcolor\")\n\n";
print $py
"ax2.tick_params(width='2')\nax3.tick_params(width='2')\nax3.minortick
s_off()\n\nplt.xticks(fontname='arial',fontweight='bold',fontsize=12)\
nplt.yticks(fontname='arial',fontweight='bold',fontsize=12)\n\n";
```

```
print $py "axes5 = ax2.twinx()\naxes5.set_ylabel('Goodman 2.7',
fontdict=font14,rotation=270,
labelpad=15)\naxes5.yaxis.set_ticks([])\n\naxes6 =
ax3.twinx()\naxes6.set_ylabel('Goodman 3.2.1',
fontdict=font14,rotation=270,
labelpad=15)\naxes6.yaxis.set_ticks([])\n\n";
print $py
"plt.sca(ax2)\nplt.yticks(fontname='arial',fontweight='bold',fontsize=
12)\nfig.savefig(\"$trait"."_$i.pdf\", dpi=400, bbox_inches='tight')";
close $py;

system("$pythonname");
unlink $pythonname;

}

}
```

Dataset 3, Script 4
#script for producing generic plots of genome architecture from .gff3 file and .gff3 file-derived gene position list

#configuration options are:
#   .gff3 file for target genome: 40
#   feature coloring for the plot: starts line 99
#   types of featuers to plot: 152-154

#STRUCTURE
#SD3/PLOTTER/
#├── SD3-S4.pl #script to produce genome architecture plots for areas of DNA between given genes based on .gff3 file
#├── input *Input.txt file(s) containing beginning and end genes to include in plot, each on a separate line
#├── output *png.txt file(s) #plot
#└── COREFILES/
#   ├── GenePositions.txt #list of gene positions (SD1-S6)
#   └── Zea_mays.B73_RefGen_v4.45.gff3 #gene information for target genome

```
use strict;
use warnings;
use Cwd qw(abs_path);
use File::Copy;
use File::Path;
use File::Basename;

my $type = fileparse_set_fstype("Unix"); #Allows spaces in file paths
my $dirname = dirname(__FILE__);
my $top = defined($dirname) ? $dirname : '.';

my %chrom;
open my $mid, '<', "$top/COREFILES/GenePositions.txt";
while (<$mid>) {

      chomp $_;
      my @columns = split("\t", $_);
      $chrom{$columns[2]} = $columns[0];

}

close $mid;

my $GFF = "$top/COREFILES/Zea_mays.B73_RefGen_v4.45.gff3";
my @Input = glob("$top/*Input.txt");

open my $in, '<', $GFF;
```

```
$/ = '###';

my %start;
my %stop;
while (<$in>) {

	if ($_ =~ m/ID=gene:(.*?);/) {

		my $gene = $1;
		my @columns = split("\t", $_);

		$start{$gene} = $columns[3];
		$stop{$gene} = $columns[4];

	}

}

close $in;
$/ = "\n";

for my $file (@Input) {

$file =~ m/(.*?)_Input.txt/;
my $trait = $1;

open my $in, '<', $file;

my $first = <$in>;
chomp $first;
my $second = <$in>;
chomp $second;
close $in;

my @coordset =
($start{$first},$stop{$first},$start{$second},$stop{$second});
my @sorted_coords = sort { $a <=> $b } @coordset;

my $Start = shift(@sorted_coords);
my $Stop = pop(@sorted_coords);
my $Length = $Stop - $Start;
my $Chrom = $chrom{$first};

my $color = '"#ffd700"';
open my $py, '>', "python.py";
print $py "import numpy as np\nimport pandas as pd\nfrom pandas import
DataFrame\nimport matplotlib.pyplot as plt\nfrom dna_features_viewer
import (\n\tGraphicFeature,\n\tGraphicRecord,\n)\n\n";
```

```
print $py "font12 = {'family' : 'Arial',\n\t'color':
'black',\n\t'weight': 'bold',\n\t'size': 12,\n\t}\n\nfont14 =
{'family' : 'Arial',\n\t'color': 'black',\n\t'weight':
'bold',\n\t'size': 14,\n\t}\n\n";
print $py "plt.rcParams[\"axes.linewidth\"] = 1.5\nfig, ax1 =
plt.subplots(\n\t1, figsize = (12, 9)\n)\n\nfeatures = [\n";

my $sandwichtop = "\tGraphicFeature(\n\t\t";
my $sandwichbottom = "\n\t),\n";
my @annotations;
my %unique;

my %color = (

      #unusual features
      RNase_MRP_RNA => '"#FFFFFF"',
      SRP_RNA => '"#FFFFFF"',
      pseudogene => '"#FFFFFF"',
      ncRNA => '"#FFFFFF"',
      ncRNA_gene => '"#FFFFFF"',

      #RNA features
      rRNA => '"#FF0000"',
      tRNA => '"#FF0000"',
      lnc_RNA => '"#FF0000"',
      pre_miRNA => '"#FF0000"',
      miRNA => '"#FF0000"',
      snRNA => '"#FF0000"',
      snoRNA => '"#FF0000"',

      #highly relevant features
      five_prime_UTR => '"#00FF00"',
      gene => '"#FFFFFF"',
      exon =>'"#0000FF"',
      CDS => '"#007FFF"', #not plotted
      three_prime_UTR =>  '"#7F00FF"',

      mRNA => '"#000000"', #not plotted

);

sub annotate {

      my @tannotations;
      my $entry = $_[0];
      my @lines = split("\n", $entry);
      my @real_lines;
```

```
	for my $line (@lines) {

		unless ($line !~ m/^\d/) { push @real_lines, $line; }

	}

	for my $line (@real_lines) {

		my @columns = split("\t", $line);
		my $type = $columns[2];
		my $beginning = $columns[3];
		my $end = $columns[4];
		my $strand = $columns[6];
		my $annot = $columns[8];
		my $name;

		$annot =~ m/^.*?:(.*?);/;
		$name = $1;

		#if ($type =~ /gene|UTR|exon|CDS|[^m]RNA/) {
		#if ($type =~ /gene|UTR|exon|[^m]RNA/) {
		if ($type =~ /gene|exon|[^m]RNA/) {

			my $ID = "$beginning"."$end"."$type";

			unless(exists($unique{$ID})) {

				$unique{$ID} = 1;

				if ($type =~ /gene/) {

					my $label = "$type: "."$name";
					my $string =
"$sandwichtop"."start=$beginning, end = $end, strand=$strand"."1,
color=$color{$type}, label=\"$label\", linewidth = 1.5, fontdict =
font12"."$sandwichbottom";
					push @tannotations, $string;

				} elsif ($type =~ /[^m]RNA/) {

					my $label = "$type: "."$name";
					my $string =
"$sandwichtop"."start=$beginning, end = $end, strand=$strand"."1,
color=$color{$type}, label=\"$label\", linewidth = 1.5, fontdict =
font12"."$sandwichbottom";
					push @tannotations, $string;

				} else {
```

```
                          my $string =
"$sandwichtop"."start=$beginning, end = $end, strand=$strand"."1,
color=$color{$type}, linewidth = 1.5"."$sandwichbottom";
                          push @tannotations, $string;

                    }

                }

            }

        }

        return @tannotations;

}

open $in, '<', $GFF;
$/ = '###';

my $flag = 0;
while (<$in>) {

        if ($_ =~ m/ID=gene:(.*?);/) {

            chomp $_;
            my $gene = $1;

            if ($flag == 1) {

                push @annotations, annotate($_);

            }

            if ($gene eq $second) {

                push @annotations, annotate($_);
                last;

            }


            if ($gene eq $first) {

                $flag = 1;
                push @annotations, annotate($_);
```

```
            }

        }

    }

    close $in;
    $/ = "\n";

    for my $entry (@annotations) {

        print $py $entry;

    }

    my $buffer_amt = $Length/100;
    my $buffernear = $Start-$buffer_amt;
    my $bufferfar = $Length+(2*$buffer_amt);

    print $py "]\n\nrecord =
    GraphicRecord(sequence_length=$bufferfar,first_index=$buffernear,
    features=features)\nrecord.plot(ax=ax1, with_ruler=False,
    strand_in_label_threshold=4)\n\n";
    print $py "ax1.set_xlabel(\"Chromosome $Chrom Position, bp\",
    fontdict=font14)\nax1.tick_params(width='2')\nax1.minorticks_off()\npl
    t.xticks(fontname='arial',fontweight='bold',fontsize=12)\n\n";
    print $py "fig.savefig(\"$trait.pdf\", dpi=400, bbox_inches='tight')";
    close $py;

    system("python.py");
    unlink "python.py";

}
```
